# Supplementary material for: Programmable nano-reactors for stochastic sensing
Source: Nat Commun. 2021 Oct 4;12:5811. doi: 10.1038/s41467-021-26054-9 (PMC8490433; doi:10.1038/s41467-021-26054-9)
Supplement: Supplementary file 1 — Supplementary Information [file 41467_2021_26054_MOESM1_ESM.docx]

Supplementary Information for

Programmable Nano-Reactors for Stochastic Sensing

Wendong Jia,^1,2^ Chengzhen Hu,^1,2^ Yuqin Wang, ^1,2^ Yuming Gu,^1,2^ Guangrui Qian,^3^ Xiaoyu Du, ^1,2^ Liying Wang, ^1,2^ Yao Liu, ^1,2^ Jiao Cao, ^1,2^ Shanyu Zhang, ^1,2^ Shuanghong Yan, ^1,2^ Panke Zhang,^1^ Jing Ma,^1^ Hong-Yuan Chen^1^ and Shuo Huang^*1,2^

^1.^ State Key Laboratory of Analytical Chemistry for Life Sciences, School of Chemistry and Chemical Engineering, Nanjing University, 210023, Nanjing, China.

^2.^ Chemistry and Biomedicine Innovation Center (ChemBIC), Nanjing University, 210023, Nanjing, China.

^3.^ Intelligence Qubic Technology Co. Ltd, Beijing, China.

* Corresponding author. Email: shuo.huang@nju.edu.cn (S.H.);

## Materials

Pentane, hexadecane, ethylenediaminetetraacetic acid (EDTA), Genapol X-80 were obtained from Sigma-Aldrich. 1,2-diphytanoyl-sn-glycero-3-phosphocholine (DPhPC) was supplied by Avanti Polar Lipids. Potassium chloride (KCl, 99.9%), sodium hydroxide (NaOH, 99.9%), cobalt sulfate heptahydrate (CoSO_4_·7H_2_O, 99.99%), nickel sulfate hexahydrate (NiSO_4_·6H_2_O, 99.9%), copper sulfate pentahydrate (CuSO_4_·5H_2_O, 99.9%), zinc sulfate heptahydrate (ZnSO_4_·7H_2_O, 99.995%), cadmium sulfate, 8/3-hydrate (CdSO_4_·8/3H_2_O, 99.99%), ethylene glycol (99.9%), glycerol (99.7%), L-lactic Acid (98%), pyridoxine (vitamin B6) (98%), 30% hydrogen peroxide solution (H_2_O_2_, GR), DL-norepinephrine hydrochloride (97%), DL-epinephrine hydrochloride (98%), DL-isoproterenol hydrochloride (99%), 3-azidopropylamine (95%), sodium sulfate anhydrous (Na_2_SO_4_, 99%), dimethyl sulfoxide (DMSO, 99.9%) and dimethyl sulfoxide-d6 (DMSO-d6, D.99.9% +0.03%TMS) were from Aladdin (China). Methylboronic acid (97%), catechol (99.5%), resorcinol (AR) and acetonitrile (MeCN, 99.9%) were from Macklin (China). Hydrochloric acid (HCl), acetone (Me_2_CO, 99.5%) and dichloromethane (DCM, 99.5%) were from Sinopharm (China). Sodium ascorbate (vitamin C) (99%), 4-(2-hydroxyethyl)-1-piperazine ethanesulfonic acid (HEPES, 99%) were purchased from Shanghai Yuanye Bio-Technology (China). 4-(Azidomethyl)benzeneboronic acid pinacol ester (95%) was from Alfa Aesar (U.S.). Remdesivir (99.74%) and remdesivir metabolite (99.87%) were purchased from MedChemExpress (Monmouth Junction, NJ, USA).

*E. coli* strain BL21 (DE3) was from Biomed (China). Streptavidin was from New England Biolabs. Dioxane-free isopropyl-β-D-thiogalactopyranoside (IPTG), kanamycin sulfate and tris-(Hydroxy-methyl) aminomethane (Tris) were from Solarbio Biotechnology (China). Luria-Bertani broth and Luria-Bertani agar were from Hopebio (China). Precision Plus ProteinTM Dual Color Standards and TGXTM FastCastTM Acrylamide Kit (12%) were purchased from Bio-Rad.

The monomeric DNA phosphoramidite, 5-ethynyl-dU-CE phosphoramidite was purchased from Glen Research (U.S.), and the alkyne-containing oligonucleotide 14TAK **(Supplementary Table 1)** was synthesized by Shanghai Generay Biotech Co., Ltd. All other DNA oligonucleotides were synthesized by Genscript (New Jersey, U.S.). Full sequences are listed in **Supplementary Table 1**.

## Supplementary Table 1| Sequence context of all PNRSS strands in this study.

| **PNRSS Strand** | **Sequence** |
| --- | --- |
| **14TAK-NTS** | 5’-biotin TEG- TTTTTTTTTTT**XX(TAK)XX**-3’ |
| **13G/14G** | 5’-biotin TEG- TTTTTTTTTT**XXGGXX**TTTTTTTTTTTTTTTTTTTTTTTTTTTTTTTTTTTTTTTTTTTT-3’ |
| **14A** | 5’-biotin TEG- TTTTTTTTTTT**XXAXX**TTTTTTTTTTTTTTTTTTTTTTTTTTTTTTTTTTTTTTTTTTTT-3’ |
| **14G** | 5’-biotin TEG- TTTTTTTTTTT**XXGXX**TTTTTTTTTTTTTTTTTTTTTTTTTTTTTTTTTTTTTTTTTTTT-3’ |
| **14X** | 5’-biotin TEG- TTTTTTTTTTT**XXXXX**TTTTTTTTTTTTTTTTTTTTTTTTTTTTTTTTTTTTTTTTTTTT-3’ |
| **14TAK** | 5’-biotin TEG- TTTTTTTTTTT**XX(TAK)XX**TTTTTTTTTTTTTTTTTTTTTTTTTTTTTTTTTTTTTTTTTTTT-3’ |
| **14TAZ** | 5’-biotin TEG- TTTTTTTTTTT**XX(TAZ)XX**TTTTTTTTTTTTTTTTTTTTTTTTTTTTTTTTTTTTTTTTTTTT-3’ |
| **14PBA** | 5’-biotin TEG- TTTTTTTTTTT**XX(PBA)XX**TTTTTTTTTTTTTTTTTTTTTTTTTTTTTTTTTTTTTTTTTTTT-3’ |

**Footnotes:**

1. The reaction sections are marked with bold fonts in each sequence.
2. Natural DNA bases or their combinations such as **A**, **G** or **GG** can serve as fixed reactants.
3. **X** represents an abasic site, which is incapable of binding any mobile reactant tested in this study.
4. The 5' Biotin TEG serves as the tether spot, forming tight binding with a streptavidin stopper.


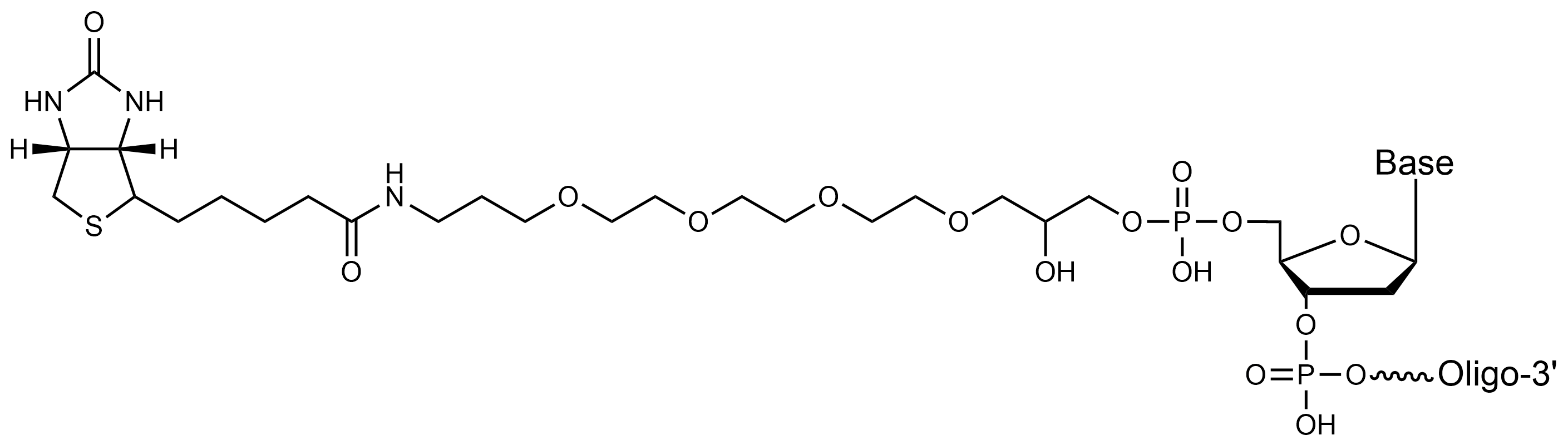


1. **(TAK)** is an alkyne-modified thymine analogue (5-ethynyl deoxyUridine, Glen Research, U.S.), serving as a universal connector to introduce functional azides. The chemical structure of **(TAK)** is provided below:


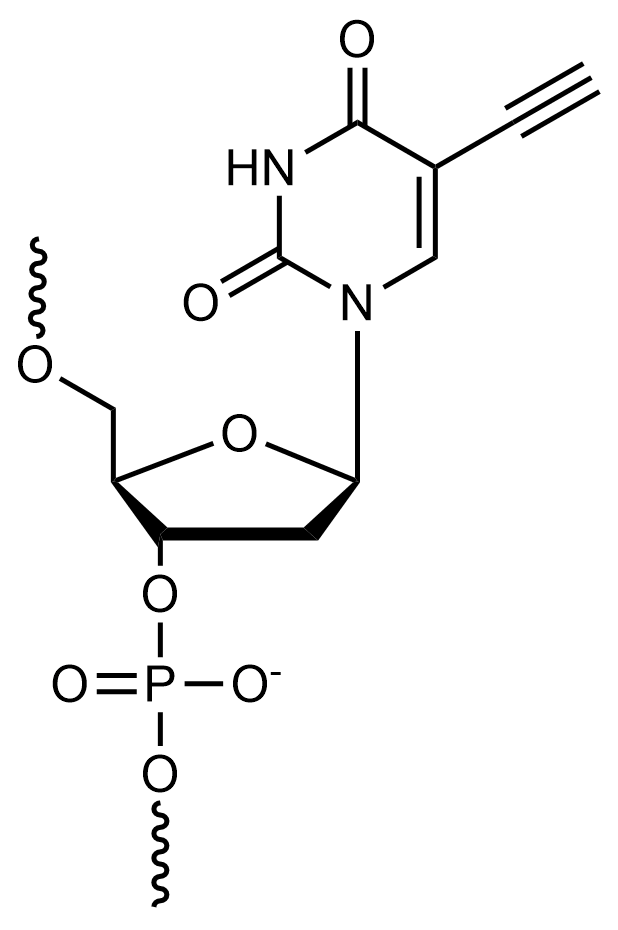


1. **(TAZ)** stands for 1,2,3-triazole. The chemical structure of the nucleotide containing a **TAZ** is provided below. Details of its chemical synthesis and characterizations are provided in **Methods** and **Supplementary fig. 11-12.**


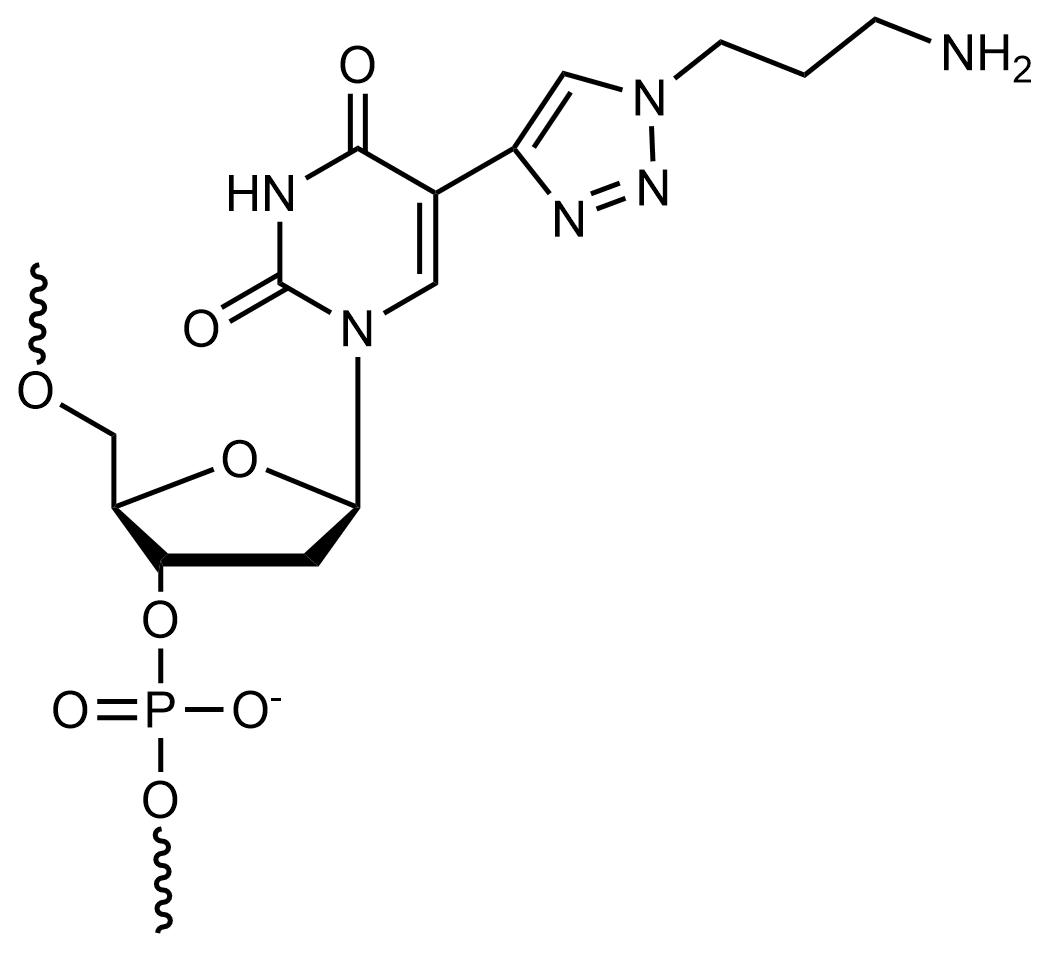


1. **(PBA)** stands for phenylboronic acid. The chemical structure of the nucleotide containing a **PBA** is provided below. Details of its chemical synthesis and characterizations are provided in **Methods** and **Supplementary fig. 19-20.**


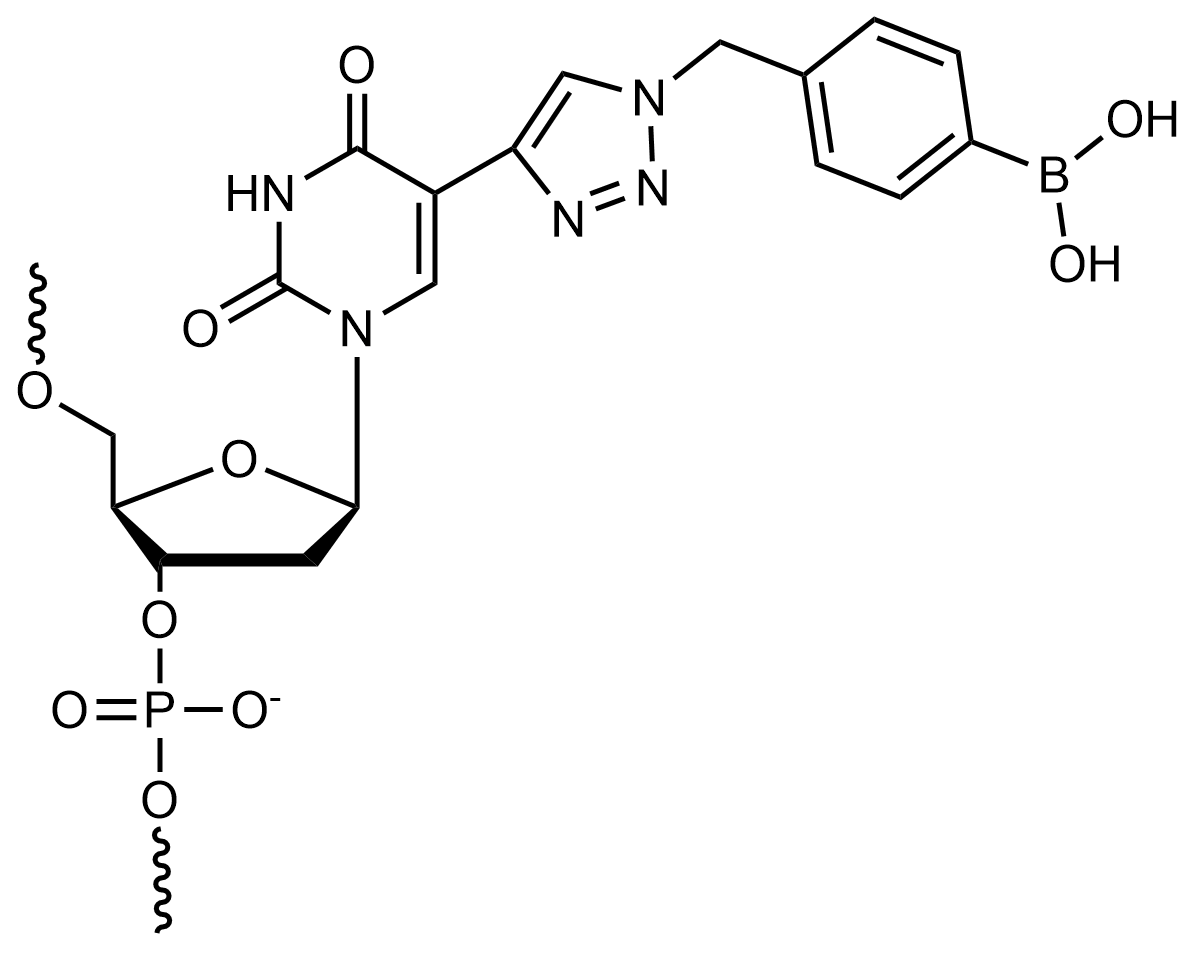


**Supplementary Table** 2| Statistics of $\bar{I_{p}}$ of 14X, 14A, 14G or 13/14G blockages. The static pore blockage measurements were performed as described in **Methods**. A buffer of 1.5 M KCl, 10 mM HEPES, pH 7.0 was applied. Each PNRSS strand was added to *cis* with a final concentration of 20 nM. $I_{p}$ was measured when a +180 mV bias was applied **(Supplementary fig. 4)**. 500 events were acquired during each measurement. Three independent measurements (N=3) were performed for each condition to produce the statistics.

| **PNRSS strand** | $\bar{\boldsymbol{I}_{\boldsymbol{p}}}$ **(pA)** |
| --- | --- |
| 14X | 143.2±1.2 |
| 14A | 144.5±1.3 |
| 14G | 145.9±0.4 |
| 13G/14G | 160.3±0.3 |

## Supplementary Table **3|** The relative energy of (dGMP)_2_-Ni-4wt and (dGMP)_2_-Ni-5wt.

| **Systems** | **E_LS_ (a.u.)** | **E_HS_ (a.u.)** | **ΔE (kcal/mol)** |
| --- | --- | --- | --- |
| (dGMP)_2_-Ni-4wt | -2890.99584 | -2891.06616 | -44.13 |
| (dGMP)_2_-Ni-5wt | -2967.38078 | -2967.43951 | -36.85 |

**Supplementary Table** 4| Statistics of $\bar{\Delta I}$and $\tau_{off}$ of Ni^2+^ binding to 14A, 14G or 13G/14G. The PNRSS measurements were performed as described in **Supplementary fig. 7** (14A), **Supplementary fig. 9** (14G) and **Fig. 1** (13G/14G). A buffer of 1.5 M KCl, 10 mM HEPES, pH 7.0 was used. A +180 mV potential was continuously applied. Ni^2+^ binding to 14A results in two event populations **(Supplementary fig. 7-8)** so that the $\bar{\Delta I}$ and the $\tau_{off}$ values were derived separately. Ni^2+^ binding to 14G **(Supplementary fig. 9-10)** or 13G/14G **(Fig. 1)** respectively, reports a single event population. A minimum of 1000 events were included for each measurement. Three independent measurements (N=3) were performed for each condition to generate the statistics.

| **PNRSS strand** | $\bar{\Delta I}$ **(pA)** | $\boldsymbol{\tau}_{\boldsymbol{off}}$ **(ms)** |
| --- | --- | --- |
| 14A | -42.4±1.7 / -57±2 | 5.68±0.09 / 3.1±0.4 |
| 14G | -28.1±1.3 | 9.4±0.3 |
| 13G/14G | -60±3 | 11.0±0.2 |

**Supplementary Table** 5| Statistics of $\bar{\boldsymbol{I}_{\boldsymbol{p}}}$ for 14TAK and 14TAZ. The static pore blockage measurements were performed as described in **Methods**. A buffer of 1.5 M KCl, 10 mM HEPES, pH 7.0 was applied. Each PNRSS strand was added to *cis* with a final concentration of 20 nM. $I_{p}$ was measured when a +180 mV bias was applied **(Supplementary fig. 4)**. 500 events were acquired from each measurement. Three independent measurements (N=3) were performed for each condition to generate the statistics.

| **PNRSS strand** | $\bar{\boldsymbol{I}_{\boldsymbol{p}}}$ **(pA)** |
| --- | --- |
| 14TAK | 137.8±0.6 |
| 14TAZ | 92.5±1.6 |

**Supplementary Table** 6| Statistics of $\bar{\Delta I}$, $\tau_{off}$ and $\boldsymbol{K}_{\boldsymbol{b}}$ of Ni^2+^ or Co^2+^ binding to a TAZ. The PNRSS measurements were performed as described in **Supplementary fig. 13** (Ni^2+^) and **Supplementary fig. 15** (Co^2+^). A buffer of 1.5 M KCl, 10 mM HEPES, pH 7.0 was used. A +180 mV potential was continuously applied. A minimum of 1000 events were included for each measurement. Three independent measurements (N=3) were performed for each condition to produce the statistics.

| **Mobile Reactants** | $\bar{\Delta I}$ **(pA)** | $\boldsymbol{\tau}_{\boldsymbol{off}}$ **(ms)** | $\boldsymbol{K}_{\boldsymbol{b}}$ **(M^-1^)** |
| --- | --- | --- | --- |
| Ni^2+^ | -19.1±1.8 | 220±50 | 390±90 |
| Co^2+^ | -22±3 | 1.32±0.11 | 48±14 |

**Supplementary Table** 7| Statistics of $\bar{I_{p}}$ for PNRSS strand 14TAK and 14PBA. The static pore blockage measurements were performed as described in **Methods**. A buffer of 1.5 M KCl, 10 mM HEPES, pH 8.0 was applied. $I_{p}$ was measured when a +160 mV bias was applied. 500 events were acquired from each measurement. Three independent measurements (N=3) were performed for each condition to produce the statistics.

| **PNRSS strand** | $\bar{\boldsymbol{I}_{\boldsymbol{p}}}$ **(pA)** |
| --- | --- |
| 14TAK | 115.6±0.4 |
| 14PBA | 99.8±0.2 |

**Supplementary Table** 8| Statistics of $\bar{\Delta I}$ and $\tau_{off}$ of phenylboronic acid binding with diols. The PNRSS measurements were performed as described in **Fig. 3**. A buffer of 1.5 M KCl, 10 mM HEPES, pH 8.0 was used. Each type of mobile reactant was separately added to *trans* with a final concentration of 400 μM (catechol), 14 mM (ethylene glycol), 10 mM (glycerol), 4 mM (L-lactic acid), 1.6 mM (vitamin C), 40 μM (vitamin B6). A +160 mV bias was continuously applied during the measurement. The definition and derivation of $\bar{\Delta I}$ and $\tau_{off}$ are described in **Supplementary fig. 4**. For each measurement, $\bar{\Delta I}$ and $\tau_{off}$ values were derived from events within a 15 min continuously recorded trace. Three independent measurements (N=3) were performed for each condition to produce the statistics.

| **Mobile Reactants** | $\bar{\Delta I}$ **(pA)** | $\boldsymbol{\tau}_{\boldsymbol{off}}$ **(ms)** |
| --- | --- | --- |
| Catechol | 24.9±0.9 | 1030±80 |
| Ethylene glycol | 23.4±1.5 | 2.6±0.2 |
| Glycerol | 22.2±0.9 | 13.1±0.3 |
| L-Lactic acid | 25.2±1.2 | 15.6±1.0 |
| Vitamin C | 26±3 | 1.9±0.2 |
| Vitamin B6 | 8.1±0.5 | 41.9±1.4 |

**Supplementary Table** 9| Kinetic constants for the formation of complexes between PBA and diols. The PNRSS measurements were performed as described in **Supplementary fig. 21-32**. A buffer of 1.5 M KCl, 10 mM HEPES, pH 8.0 was used. A +160 mV potential was continuously applied. For each measurement, kinetic constant values were derived from events within a 15 min continuously recorded trace. Three independent measurements (N=3) were performed for each condition to produce the statistics.

| **Mobile Reactants** | $\boldsymbol{k}_{\boldsymbol{on}}$ **(M^-1^s^-1^)** | $\boldsymbol{k}_{\boldsymbol{off}}$ **(s^-1^)** | $\boldsymbol{K}_{\boldsymbol{b}}$ **(M^-1^)** |
| --- | --- | --- | --- |
| Catechol | 1000±300 | 0.98±0.08 | 1100±300 |
| Ethylene glycol | 43±6 | 390±40 | 0.11±0.01 |
| Glycerol | 99±8 | 76.2±1.8 | 1.30±0.08 |
| L-Lactic acid | 260±70 | 64±4 | 4.1±1.3 |
| Vitamin C | 320±70 | 530±50 | 0.60±0.12 |
| Vitamin B6 | 57580±9160 | 23.9±0.8 | 2410±400 |

**Supplementary Table** 10| Kinetic constants for catecholamine interacting with a PBA. The PNRSS measurements were performed as described in **Supplementary fig. 37-42**. A buffer of 1.5 M KCl, 10 mM HEPES, pH 8.0 was used. A +160 mV potential was continuously applied. For each measurement, kinetic constant values were derived from events within a 15 min continuously recorded trace. Three independent measurements (N=3) were performed for each condition to produce the statistics.

| **Mobile Reactants** | $\boldsymbol{k}_{\boldsymbol{on}}$ **(M^-1^s^-1^)** | $\boldsymbol{k}_{\boldsymbol{off}}$ **(s^-1^)** | $\boldsymbol{K}_{\boldsymbol{b}}$ **(M^-1^)** |
| --- | --- | --- | --- |
| Epinephrine | 9200±900 | 1.09±0.13 | 8300±300 |
| Norepinephrine | 6000±1000 | 0.98±0.11 | 6200±1500 |
| Isoprenaline | 4600±1100 | 0.63±0.02 | 7000±2000 |

**Supplementary Table** 11| Statistics of $\Delta I$ and $\tau_{off}$ of catecholamine events. The PNRSS measurements were performed as described in **Supplementary fig. 37**, **Supplementary fig. 39** and **Supplementary fig. 41**. A buffer of 1.5 M KCl, 10 mM HEPES, pH 8.0 was applied. Each mobile reactant was added to *trans* with a final concentration of 140 μM. A +160 mV bias was continuously applied during the measurement. For each measurement, $\bar{\Delta I}$ and $\tau_{off}$ values were derived from events generated from a 5 min continuously recorded trace. Three independent measurements (N=3) were performed for each condition to produce the statistics.

| **Mobile Reactants** | $\bar{\Delta I}$ **(pA)** | $\boldsymbol{\tau}_{\boldsymbol{off}}$ **(ms)** |
| --- | --- | --- |
| Norepinephrine | -21±2 | 1020±110 |
| Epinephrine | -25.9±0.5 | 930±110 |
| Isoprenaline | -32.2±0.8 | 1580±50 |

**Supplementary Table** 12| Statistics of $\bar{\Delta I}$ and $\tau_{off}$ of remdesivir and remdesivir metabolite events. The PNRSS measurements were performed as described in **Supplementary fig. 47** and **Supplementary fig. 49.** Remdesivir or remdesivir metabolite were added to *trans* with a final concentration of 80 μM or 500 μM in separate, independent measurements. A +160 mV bias was continuously applied during the measurement. For each measurement, $\bar{\Delta I}$ and $\tau_{off}$ values were derived from events generated from a 5 min continuously recorded trace. Three independent measurements (N=3) were performed for each condition to form the statistics.

| **Mobile Reactants** | $\Delta I$ **(pA)** | $\boldsymbol{\tau}_{\boldsymbol{off}}$ **(ms)** |
| --- | --- | --- |
| Remdesivir | 26.3±0.6 | 100±50 |
| Remdesivir Metabolite | 26.6±1.5 | 22±7 |

**Supplementary Table** 13| Kinetic constants of remdesivir and remdesivir metabolite binding to a PBA. The PNRSS measurements were performed as described in **Supplementary fig. 47** and **Supplementary fig. 49**. A buffer of 1.5 M KCl, 10 mM HEPES, pH 8.0 was used. A +160 mV potential was continuously applied. For each measurement, kinetic constant values were derived from events within a 15 min continuously recorded trace. Three independent measurements (N=3) were performed for each condition to produce the statistics.

| **Mobile Reactants** | $\boldsymbol{k}_{\boldsymbol{on}}$ **(M^-1^s^-1^)** | $\boldsymbol{k}_{\boldsymbol{off}}$ **(s^-1^)** | $\boldsymbol{K}_{\boldsymbol{b}}$ **(M^-1^)** |
| --- | --- | --- | --- |
| Remdesivir | 3760±1270 | 1.03±0.05 | 3610±1070 |
| Remdesivir metabolite | 440±30 | 47±13 | 10±2 |

**Supplementary Table** **14| limit of detection.** In this paper, the limit of detection is defined as the minimum concentration of the analyte required so that at least 5 events were detected within 10 minutes of measurement. All PNRSS measurements were performed as described in **Methods**. A buffer of 1.5 M KCl, 10 mM HEPES, pH 7.0 was used and a +180 mV potential was continuously applied for all measurements with 14A, 14G, 13G/14G or 14TAZ. A buffer of 1.5 M KCl, 10 mM HEPES, pH 8.0 was used and a +160 mV potential was continuously applied for all measurements with 14PBA.

| **PNRSS strand** | **Mobile Reactants** | **limit of detection (μM)** |
| --- | --- | --- |
| 14A | Ni^2+^ | 0.2 |
| 14G | Ni^2+^ | 0.2 |
| 13G/14G | Ni^2+^ | 0.2 |
| 14TAZ | Ni^2+^ | 1 |
|  | Co^2+^ | 1 |
| 14PBA | Catechol | 2 |
|  | Ethylene glycol | 200 |
|  | Glycerol | 150 |
|  | L-Lactic acid | 60 |
|  | Vitamin C | 40 |
|  | Vitamin B6 | 0.4 |
|  | Tris | 20 |
|  | Epinephrine | 1 |
|  | Norepinephrine | 1 |
|  | Isoprenaline | 1 |
|  | Remdesivir | 2 |
|  | Remdesivir metabolite | 20 |

**Supplementary Table** **15| Summary of binding constants** $\boldsymbol{K}_{\boldsymbol{b}}$ **(M^-1^) between previous reports and PNRSS.**

| **Fixed**  **Reactant** | **Mobile**  **Reactant** | **Previous Reports On Binding Constants** | **Binding Constants**  $\boldsymbol{K}_{\boldsymbol{b}}$ **(M^-1^)**  **In Previous Reports** | **Binding Constants**  $\boldsymbol{K}_{\boldsymbol{b}}$ **(M^-1^)**  **In PNRSS Measurements** | **Origin of Discussions** |
| --- | --- | --- | --- | --- | --- |
| PBA | 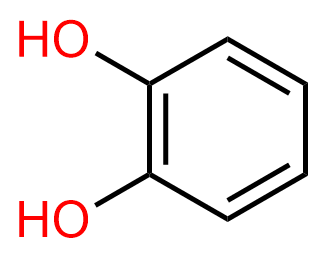 | Axthelm J, Askes SHC, Elstner M, G UR, Görls H, Bellstedt P, J. Am. Chem. Soc. 139, 11413-11420 (2017). | 3980 ± 2370 | 1100 ± 300 | **Fig. 3b** |
|  | 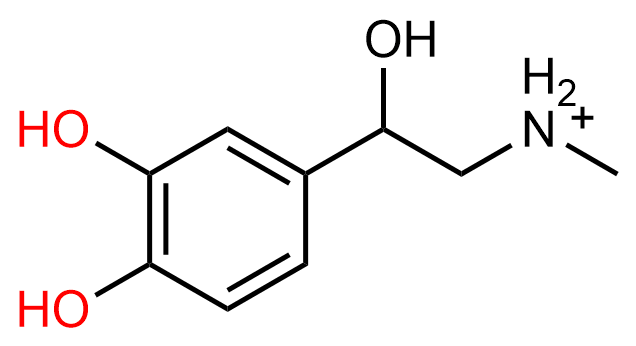 | Secor KE, Glass TE, Org. Lett. 6, 3727-3730 (2004). | 5000 | 8300 ± 300 | **Fig. 5b** |
|  | 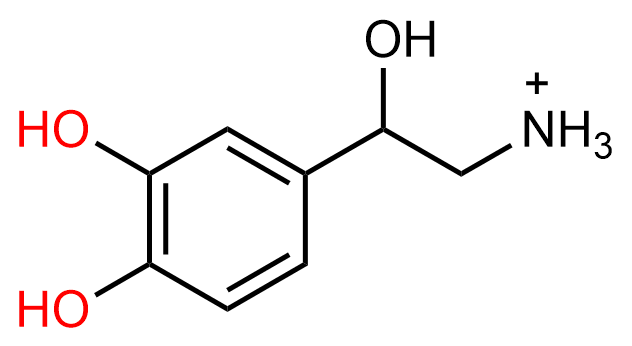 | Secor KE, Glass TE, Org. Lett. 6, 3727-3730 (2004). | 6500 | 6200 ± 1500 | **Fig. 5b** |
|  | 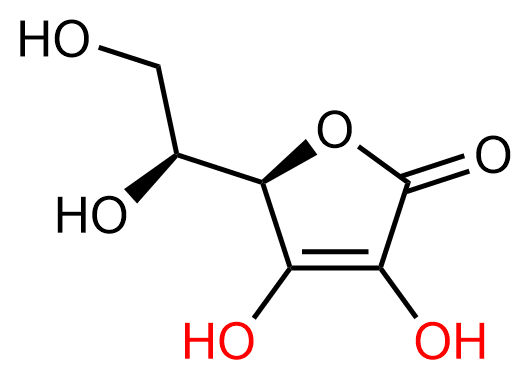 | Ali SR, Parajuli RR, Ma Y, Balogun Y, He H, J. Phys. Chem. B. 111, 12275-12281 (2007). | 21 ± 1.8 | 0.60 ± 0.12 | **Fig. 3f** |

## Supplementary Table 16| Summary of PNRSS measurements in this manuscript.

| **Index** | **Fixed**  **Reactant** | **Mobile**  **Reactant** | **Previous Reports On Chemical Reaction** | **Previous Reports In**  **Single Molecule** | **Origin of Discussions** |
| --- | --- | --- | --- | --- | --- |
| 1 | 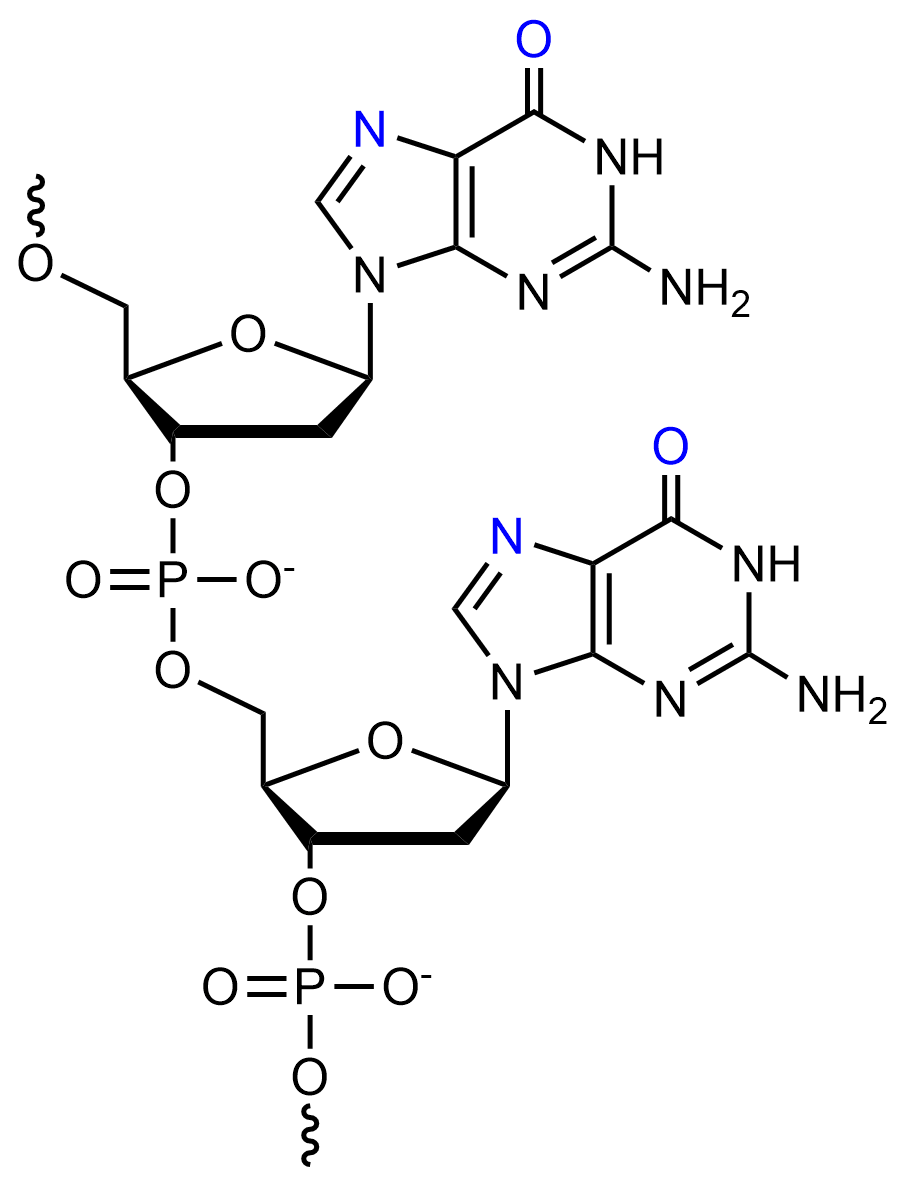 | Ni^2+^ | C. M. Mikulski, L. Mattucci, Y. Smith, T. B. Tran, N. M. Karayannis, Inorganica Chimica Acta 80, 127-133 (1983). | N/A | **Fig. 1d** |
| 2 | 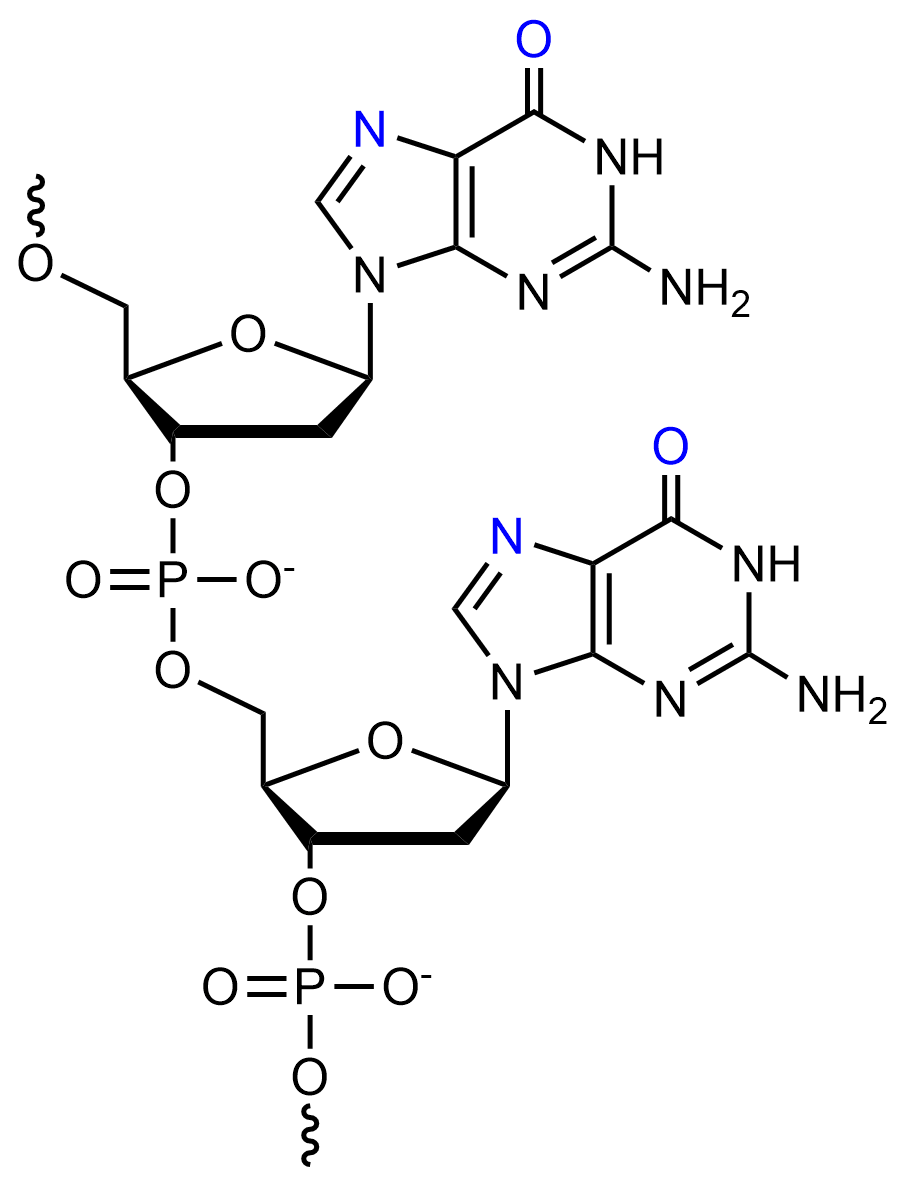 | Co^2+^ | M. S. Masoud, A. A. Soayed, A. E. Ali, Spectrochim. Acta, Part A, 60, 1907-1915 (2004). | N/A | **Supplementary fig. 6d** |
| 3 | 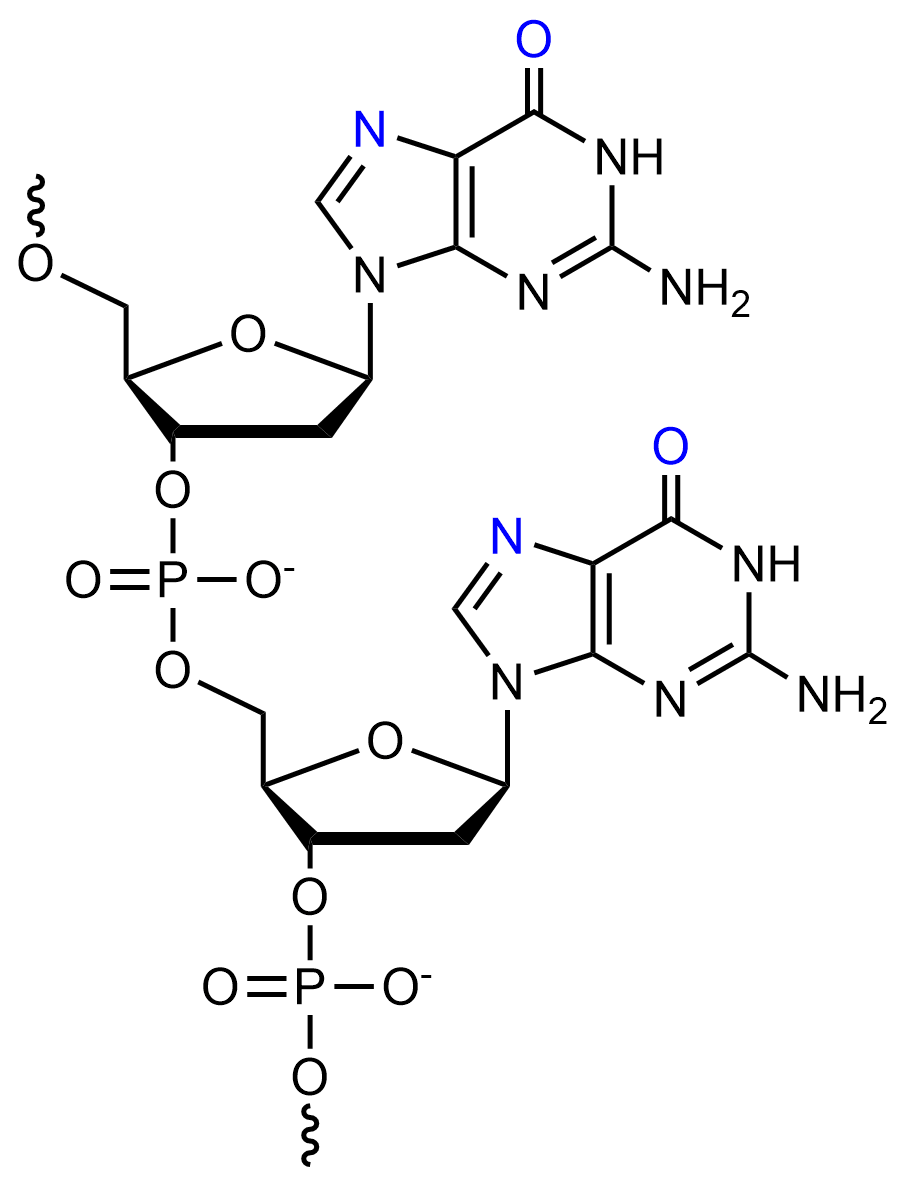 | Cu^2+^ | M. S. Masoud, A. A. Soayed, A. E. Ali, Spectrochim. Acta, Part A, 60, 1907-1915 (2004). | N/A | **Supplementary fig. 6e** |
| 4 | 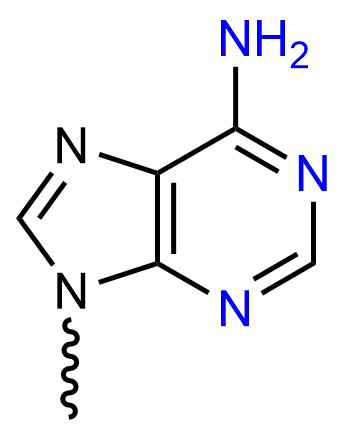 | Ni^2+^ | F. Huq, M. C. R. Peter, J. Inorg. Biochem. 78, 217-226 (2000). | N/A | **Supplementary fig. 7** |
| 5 | 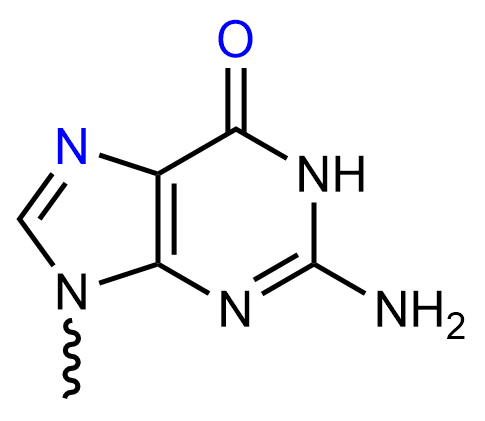 | Ni^2+^ | F. Huq, M. C. R. Peter, J. Inorg. Biochem. 78, 217-226 (2000). | N/A | **Supplementary fig. 9** |
| 6 | 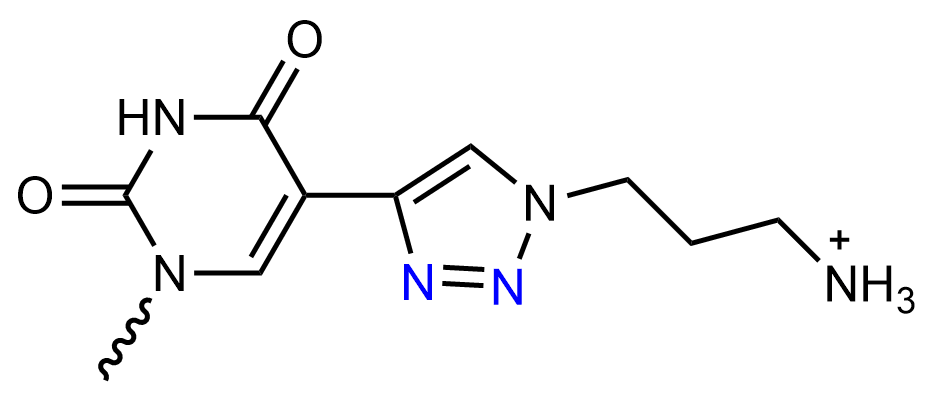 | Ni^2+^ | B. Schulze, U. S. Schubert, Chem. Soc. Rev.43, 2522-2571 (2014). | N/A | **Fig. 2b** |
| 7 | 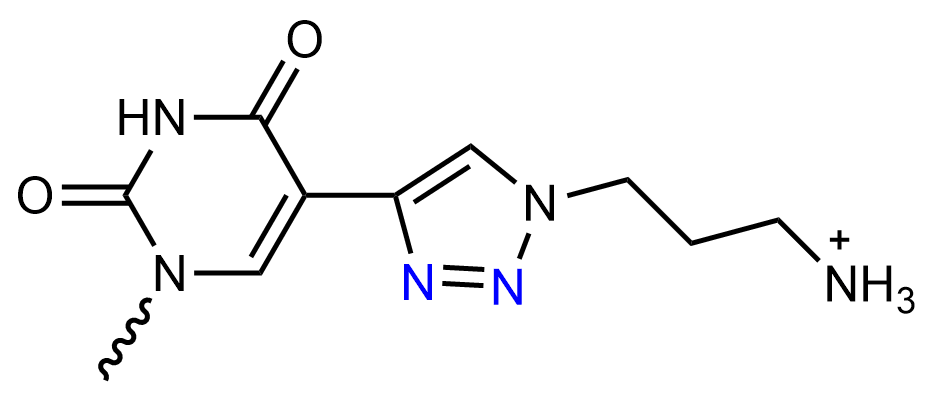 | Co^2+^ | Y. Fu et al., Chin. J. Chem. 28, 2226-2232 (2010). | N/A | **Supplementary fig. 15** |
| 8 | 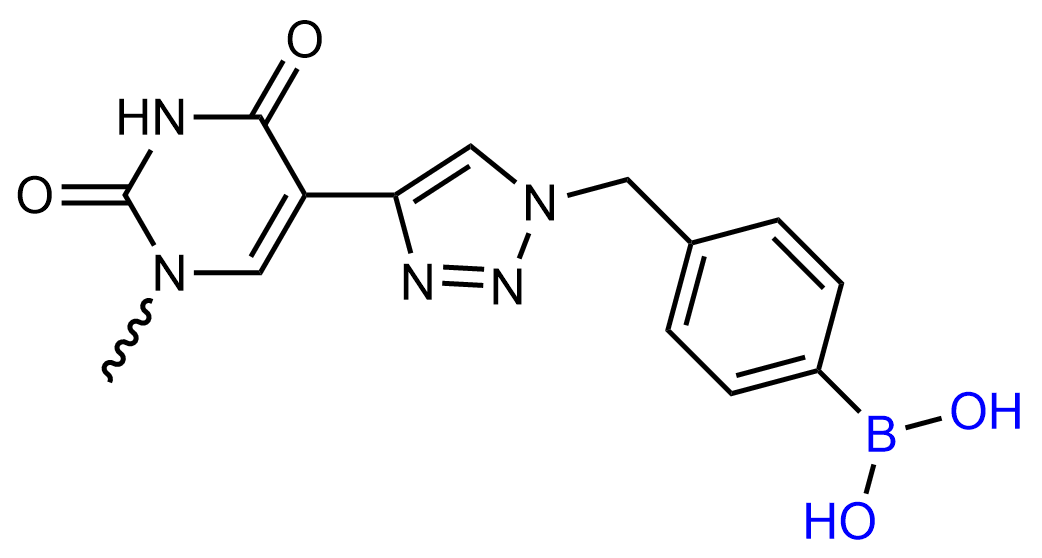 | 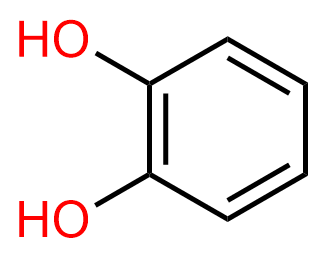 | J. P. Lorand, J. O. Edwards,  J. Org. Chem.24, 769-774 (1959). | W. J. Ramsay, H. Bayley, Angew.Chem. Int. Ed.57, 2841-2845 (2018). | **Fig. 3b** |
| 9 | 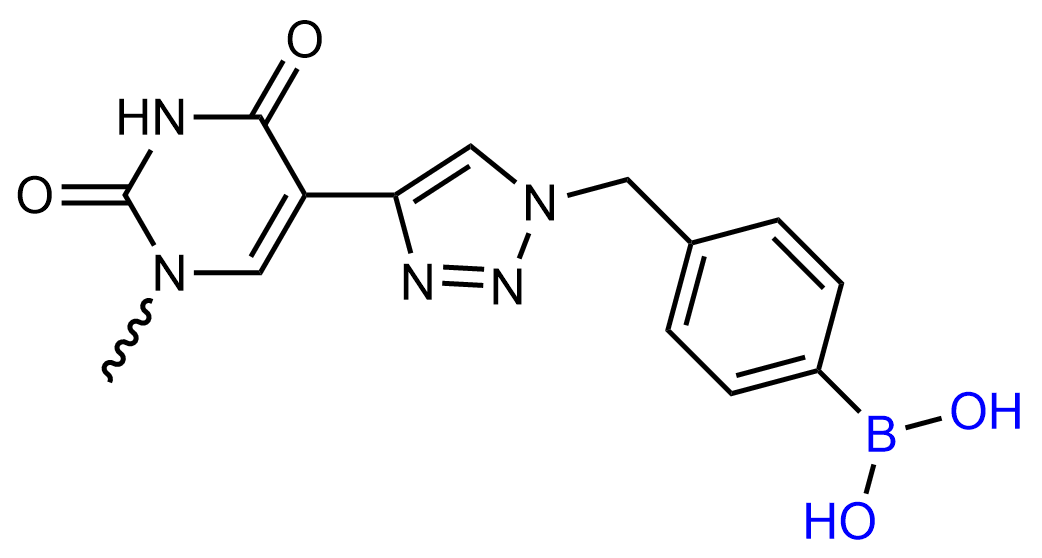 | 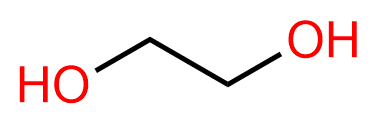 | J. P. Lorand, J. O. Edwards,  J. Org. Chem.24, 769-774 (1959). | N/A | **Fig. 3c** |
| 10 | 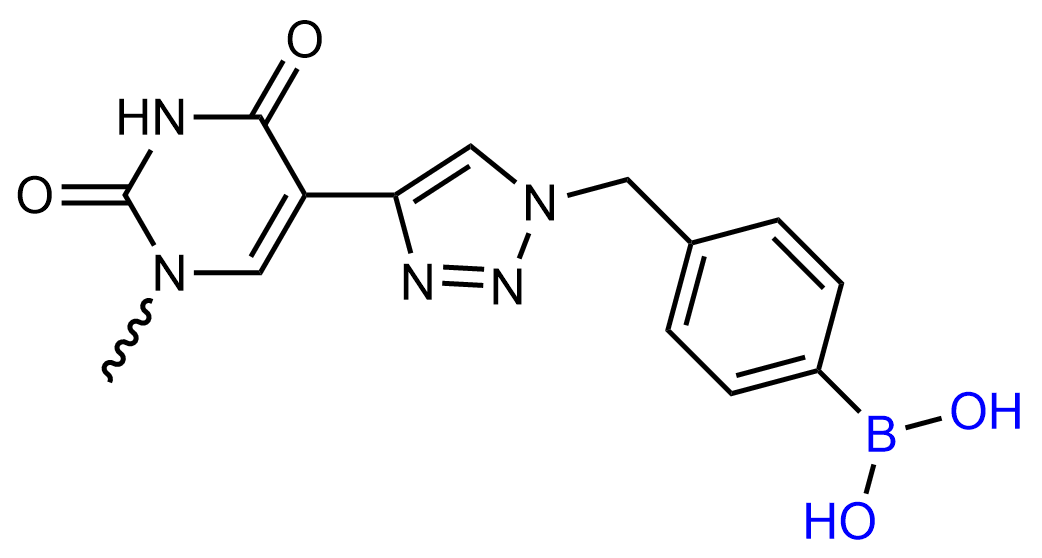 | 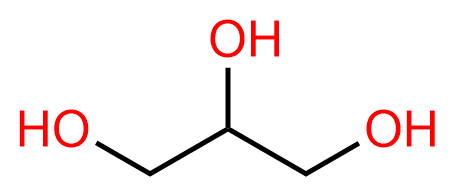 | J. P. Lorand, J. O. Edwards,  J. Org. Chem.24, 769-774 (1959). | N/A | **Fig. 3d** |
| 11 | 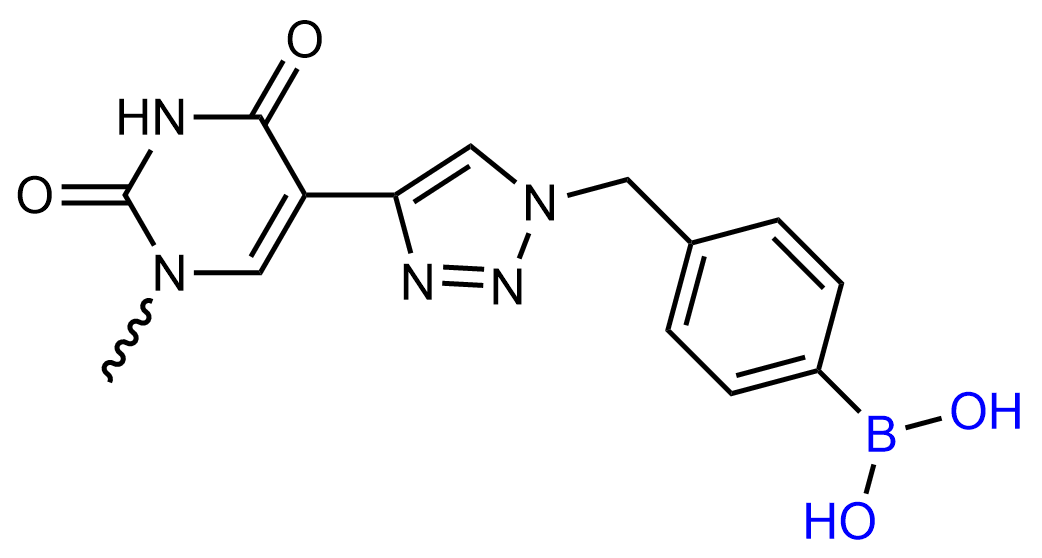 | 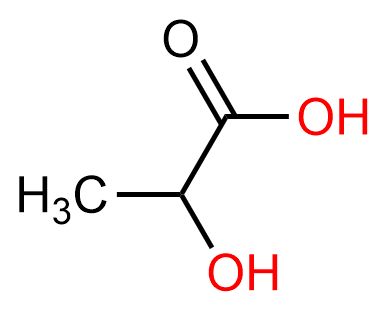 | E. Watanabe et al.,  Inorg. Chem. Commun.  13, 1406-1409 (2010). | N/A | **Fig. 3e** |
| 12 | 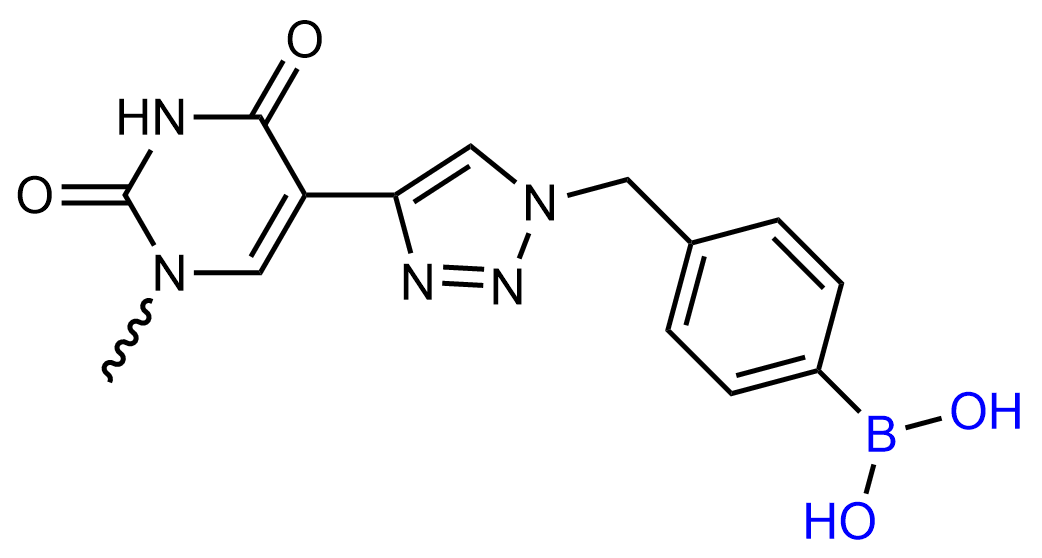 | 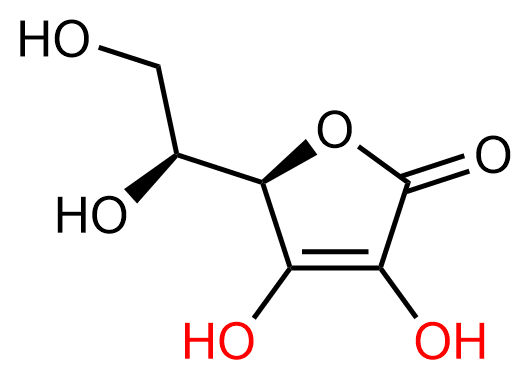 | D. A. Koese, B. Zuemreoglu-Karan, New J. Chem.33, 1874-1881 (2009). | N/A | **Fig. 3f** |
| 13 | 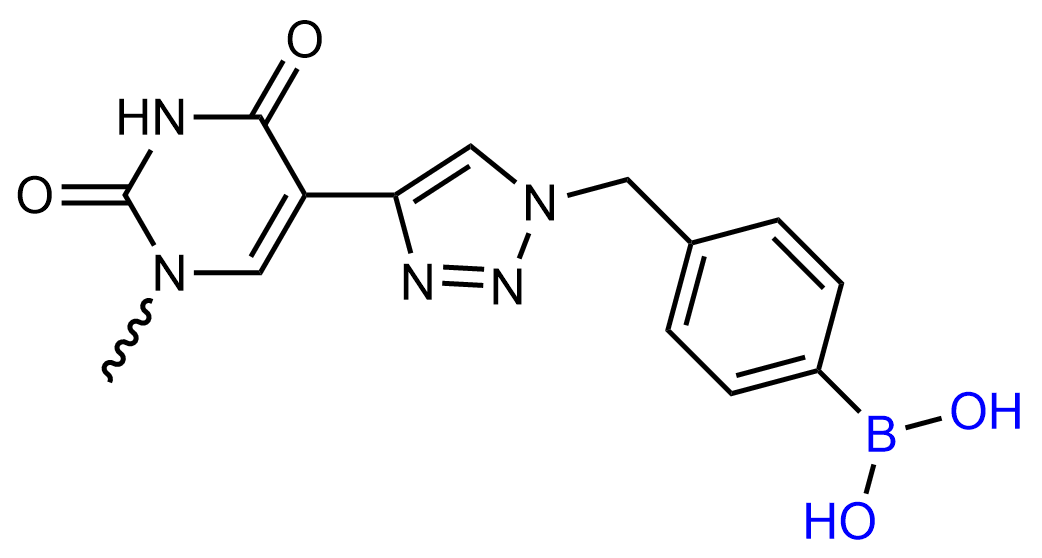 | 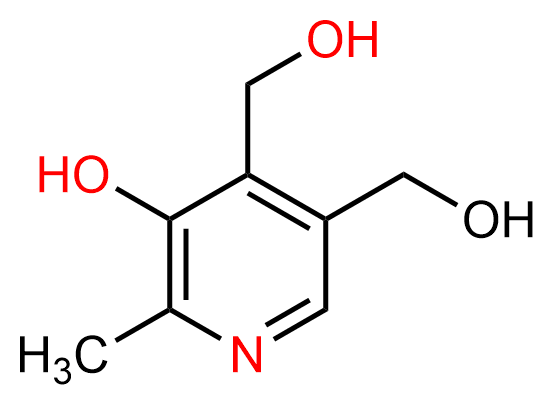 | D. A. Kose, B. Zumreoglu-Karan, O. Sahin, O. Buyukgungor, Inorg. Chim. Acta 413, 77-83 (2014). | N/A | **Fig. 3g** |
| 14 | 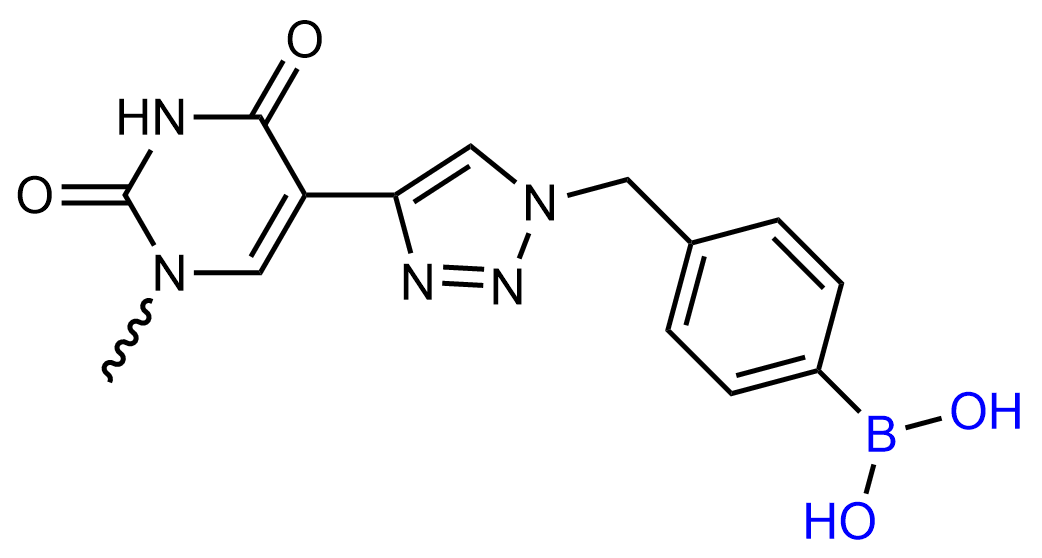 | 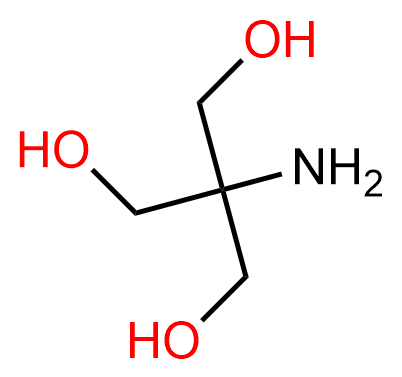 | N/A | N/A | **Supplementary fig. 33-35** |
| 15 | 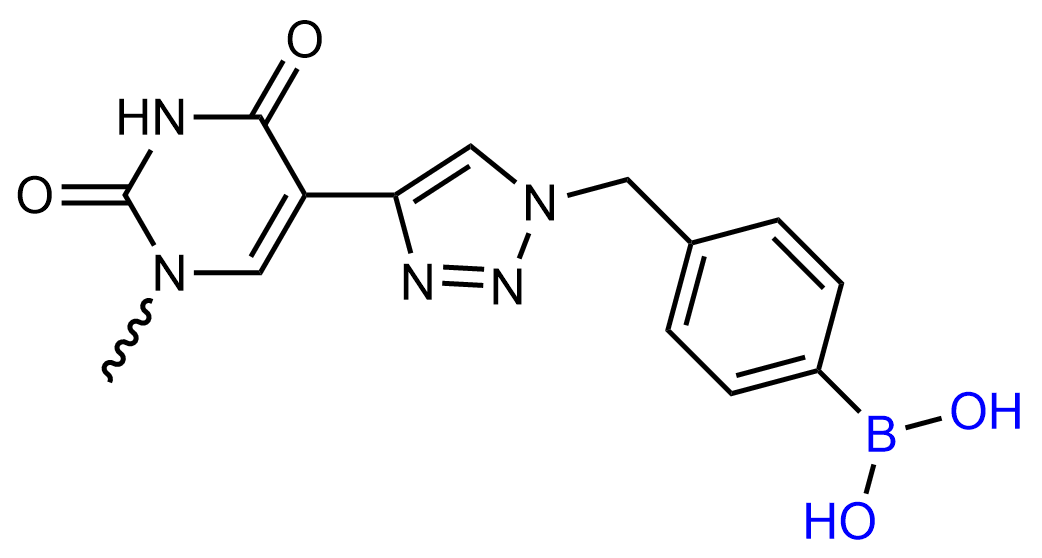 | H_2_O_2_ | A. Dutta, A. A. Ali, D. Sarma, J. Iran Chem. Soc. 16, 2379-2388 (2019). | N/A | **Fig. 4b** |
| 16 | 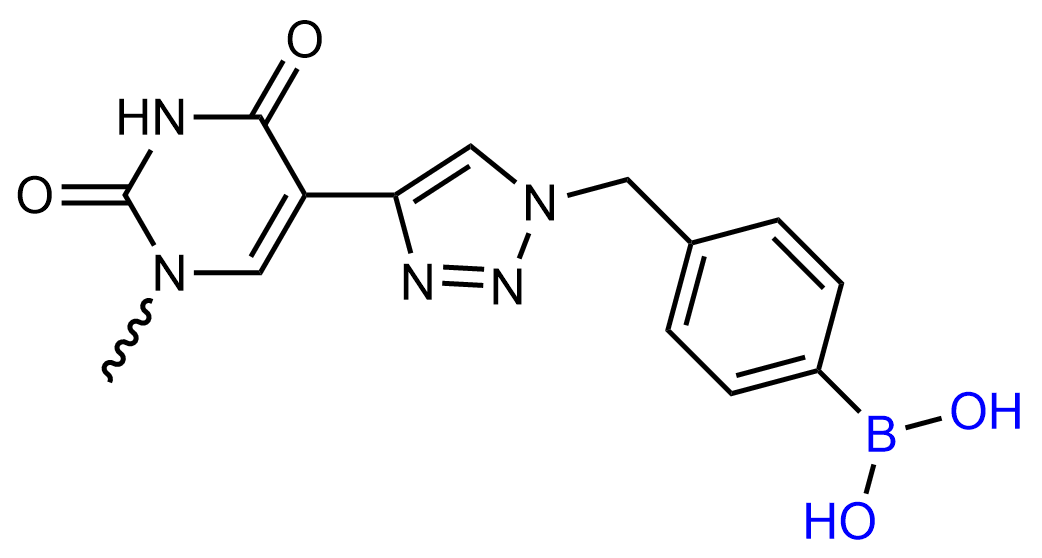 | 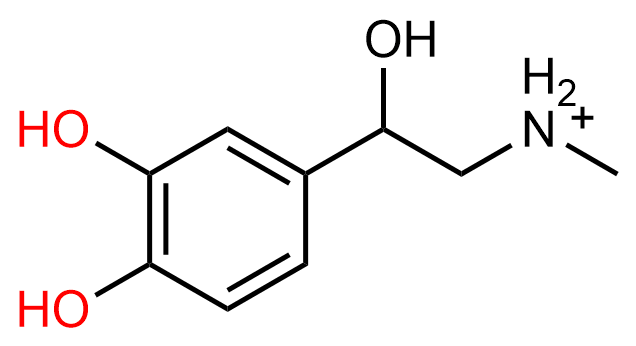 | S. Zhang, Y. Tang, Y. Chen, J. Zhang, Y. Wei, Microchimica Acta 187, (2020). | N/A | **Fig. 5b** |
| 17 | 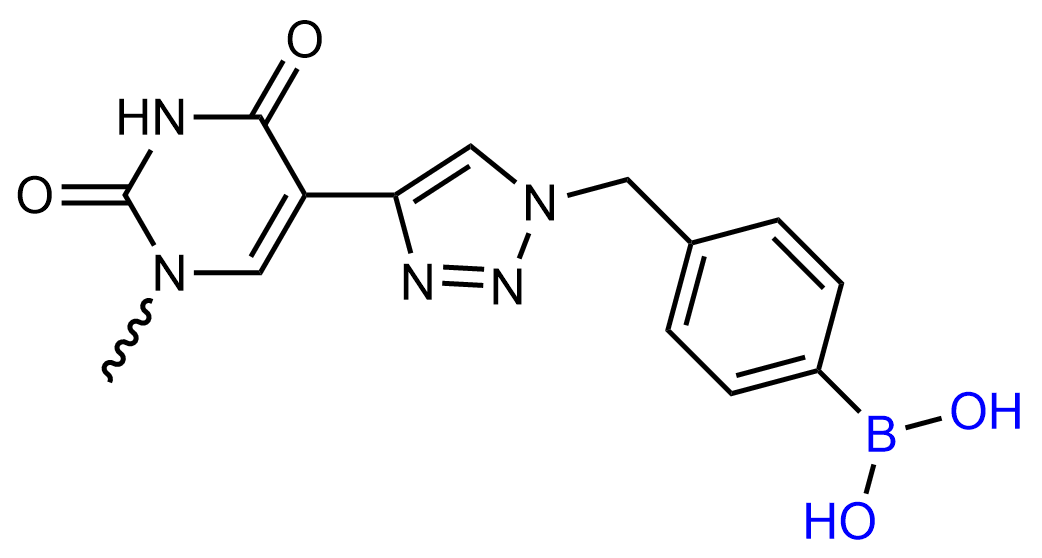 | 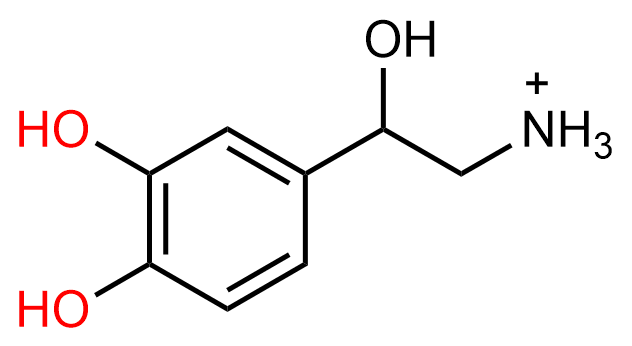 | T. Ptak, P. Mlynarz, A. Dobosz, A. Rydzewska, M. Prokopowicz, J. Mol. Struct.1040, 59-64 (2013). | N/A | **Fig. 5b** |
| 18 | 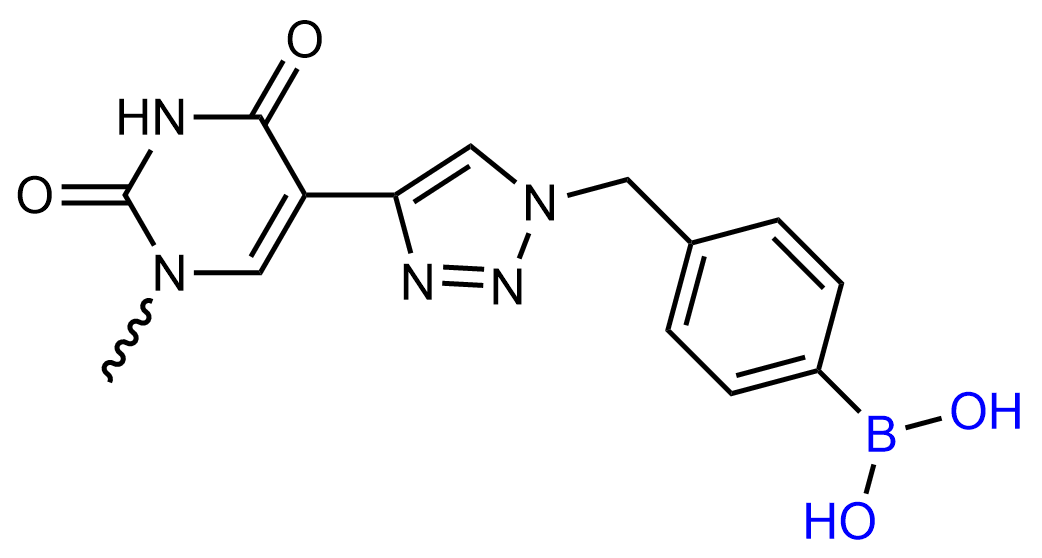 | 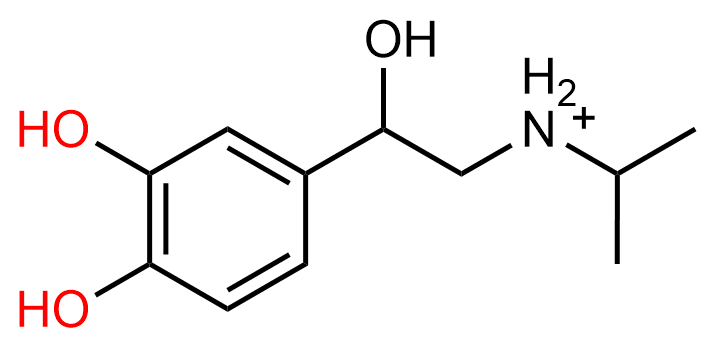 | S. Zhang, Y. Tang, Y. Chen, J. Zhang, Y. Wei, Microchimica Acta 187, (2020). | N/A | **Fig. 5b** |
| 19 | 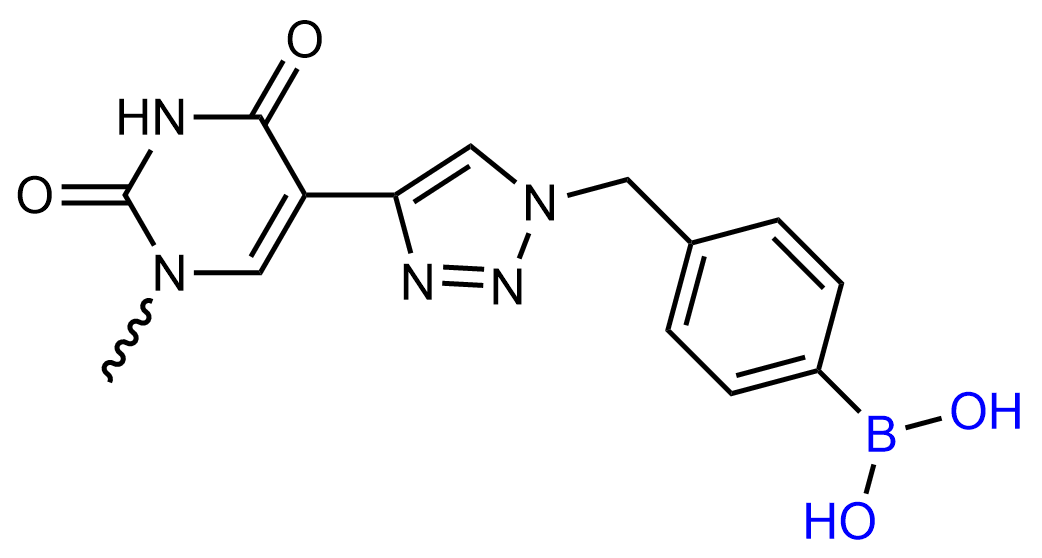 | 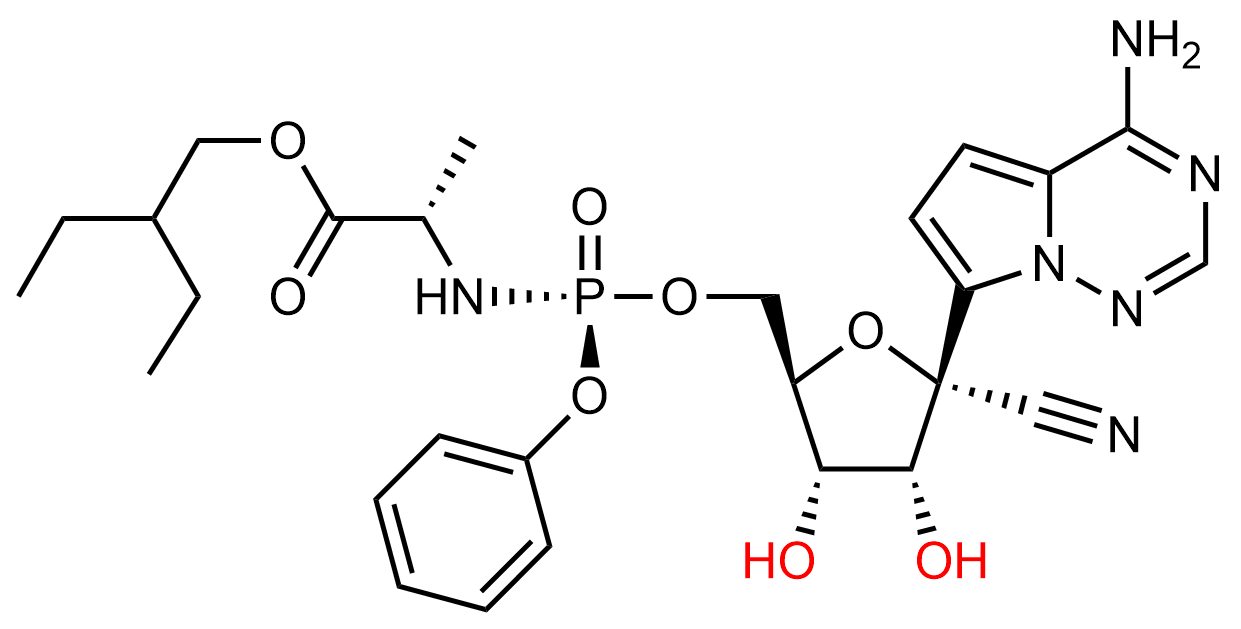 | N/A | N/A | **Fig. 6b** |
| 20 | 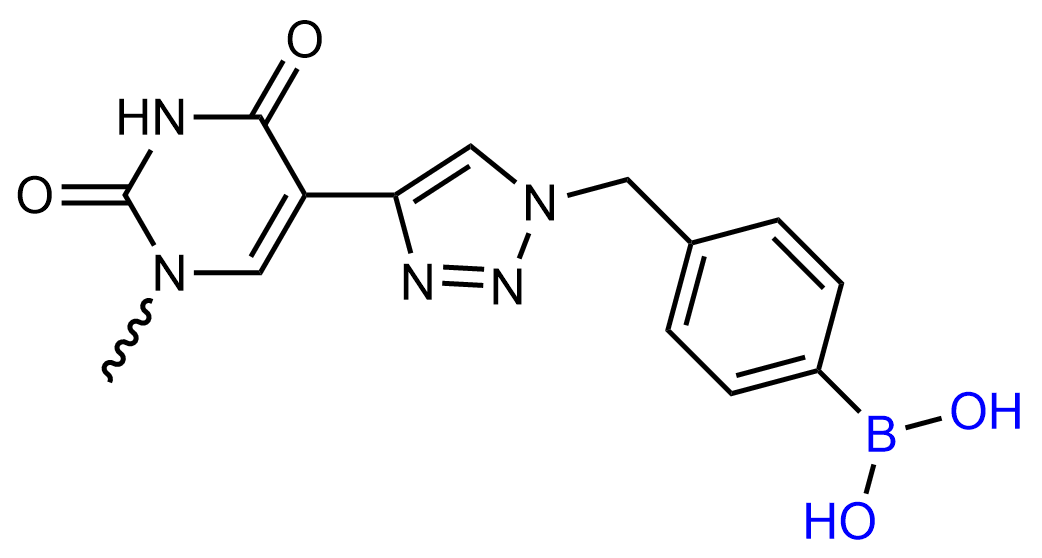 | 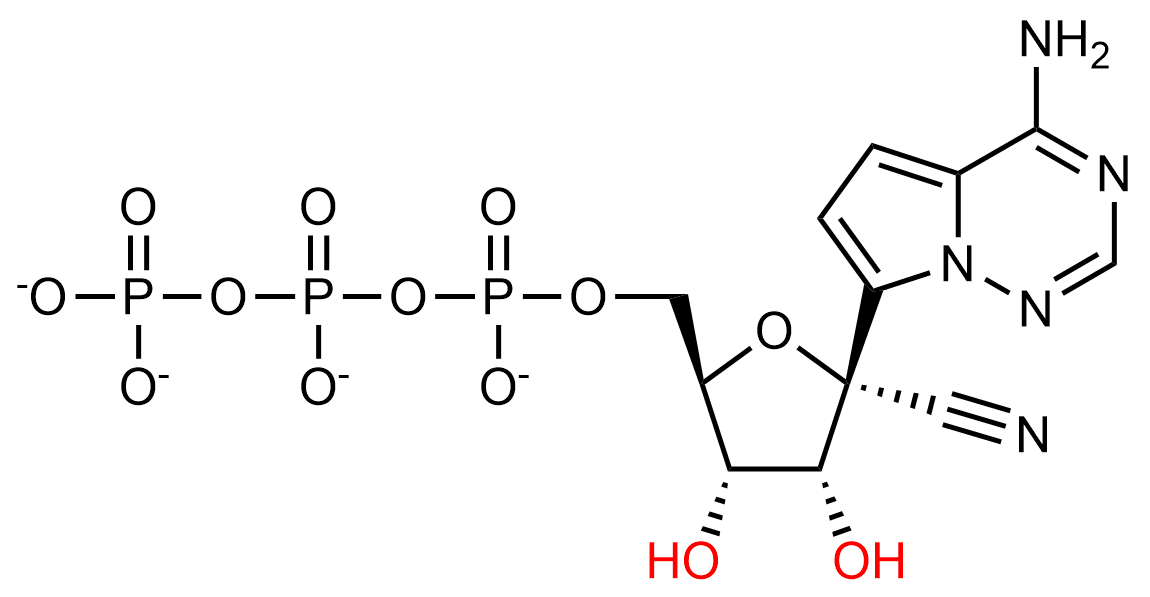 | N/A | N/A | **Fig. 6b** |

**
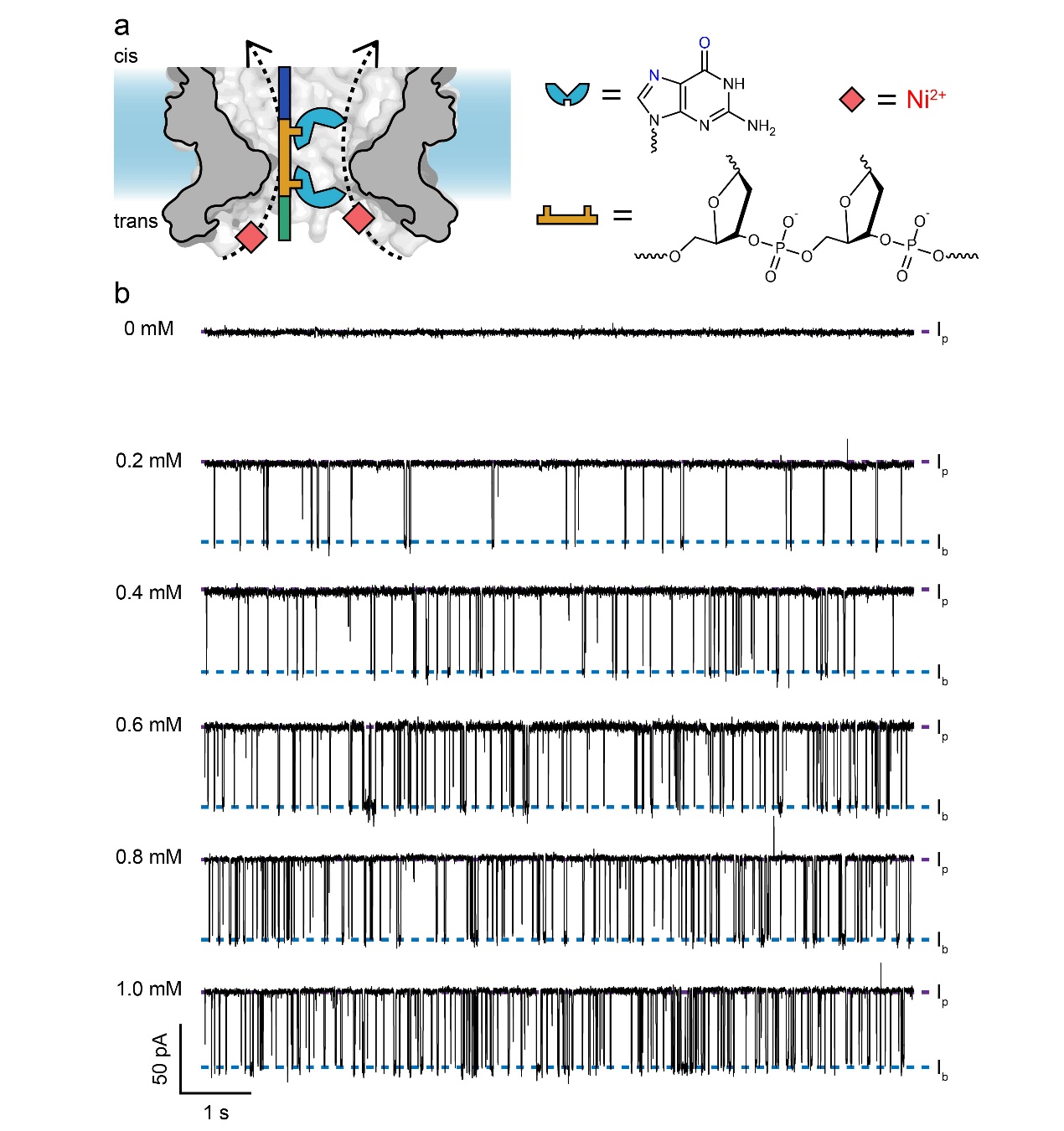
**

Supplementary fig. 1| Ni^2+^ binding to a dual guanine reactant. **a.** The schematic diagram. The PNRSS measurement was carried out as described in **Fig. 1c**. A buffer of 1.5 M KCl, 10 mM HEPES, pH 7.0. was used. A +180 mV potential was continuously applied. The PNRSS strand 13G/14G **(Supplementary Table 1)** contains two neighbouring guanines, cooperatively serving as a ligand to bind Ni^2+^. Ni^2+^, which acts as a mobile reactant, was added to *trans* reaching a desired final concentration*.* **b.** Representative traces acquired with varying Ni^2+^ concentrations. The Ni^2+^ concentrations were adjusted between 0 and 1 mM and are marked on the left of each corresponding trace, which shows that the rate of event appearance increases when the Ni^2+^ concentration is raised.

**
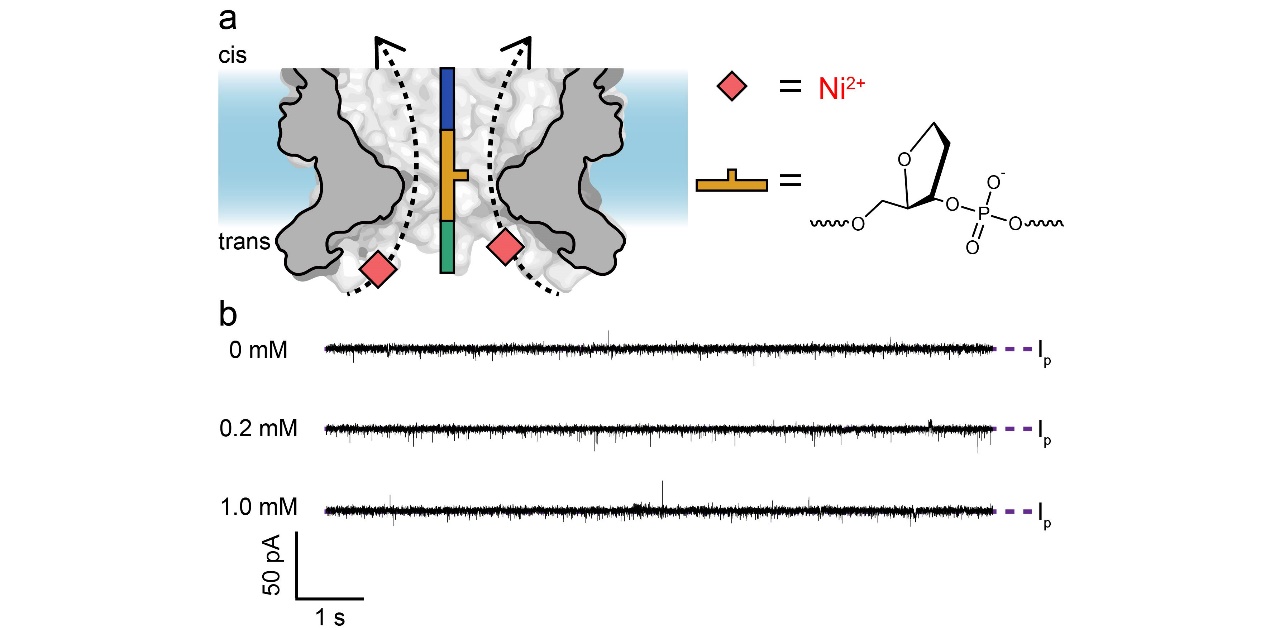
**

Supplementary fig. 2| PNRSS with no fixed reactant. **a.** The schematic diagram. The measurements were carried out as described in **Methods**. A buffer of 1.5 M KCl, 10 mM HEPES, pH 7.0 was used. A +180 mV potential was continuously applied. The PNRSS strand 14X **(Supplementary Table 1)**, in which the reaction section is composed of five consecutive abasic sites, was applied. Ni^2+^ serves as the mobile reactant. In principle, the abasic sites are incapable of binding Ni^2+^. **b.** Representative traces from corresponding PNRSS measurements. Ni^2+^ was added to *trans* with a final concentration of 0-1 mM. The final concentrations of Ni^2+^ are marked on the left of each corresponding trace. No events of Ni^2+^ binding were observed, concluding that the PNRSS strand 14X fails to report any Ni^2+^ binding events.

**
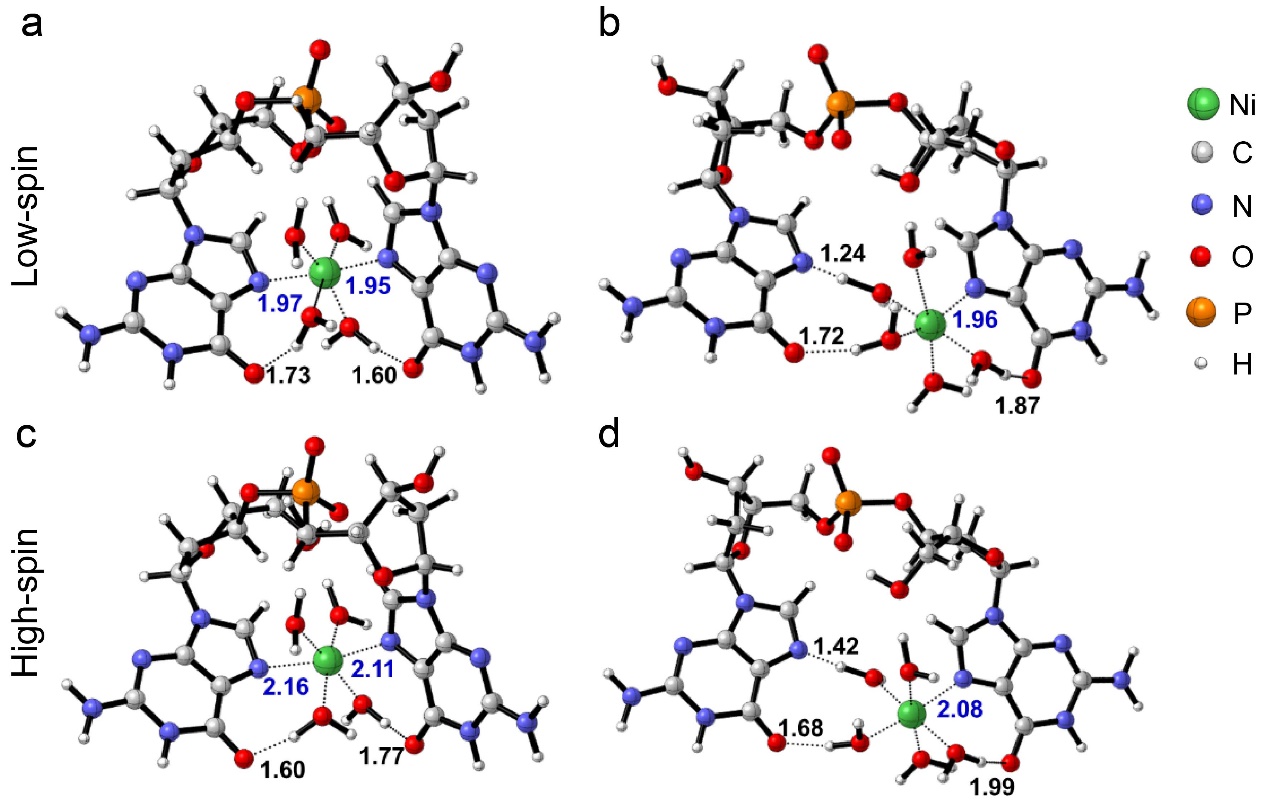
**

Supplementary fig. 3| Ni^2+^ binding with a dual guanine reactant (simulation). The optimized structures of Ni^2+^ binding with a dual guanine ligand. (dGMP)_2_-Ni-4wt with **(a)** the low-spin state, **(c)** the high-spin state, and (dGMP)_2_-Ni-5wt with **(b)** the low-spin state, **(d)** the high-spin state are shown. Water molecules which participate in the binding are also shown. Green, grey, blue, red, orange and white balls represent Ni, C, N, O, P, and H atoms respectively.

**
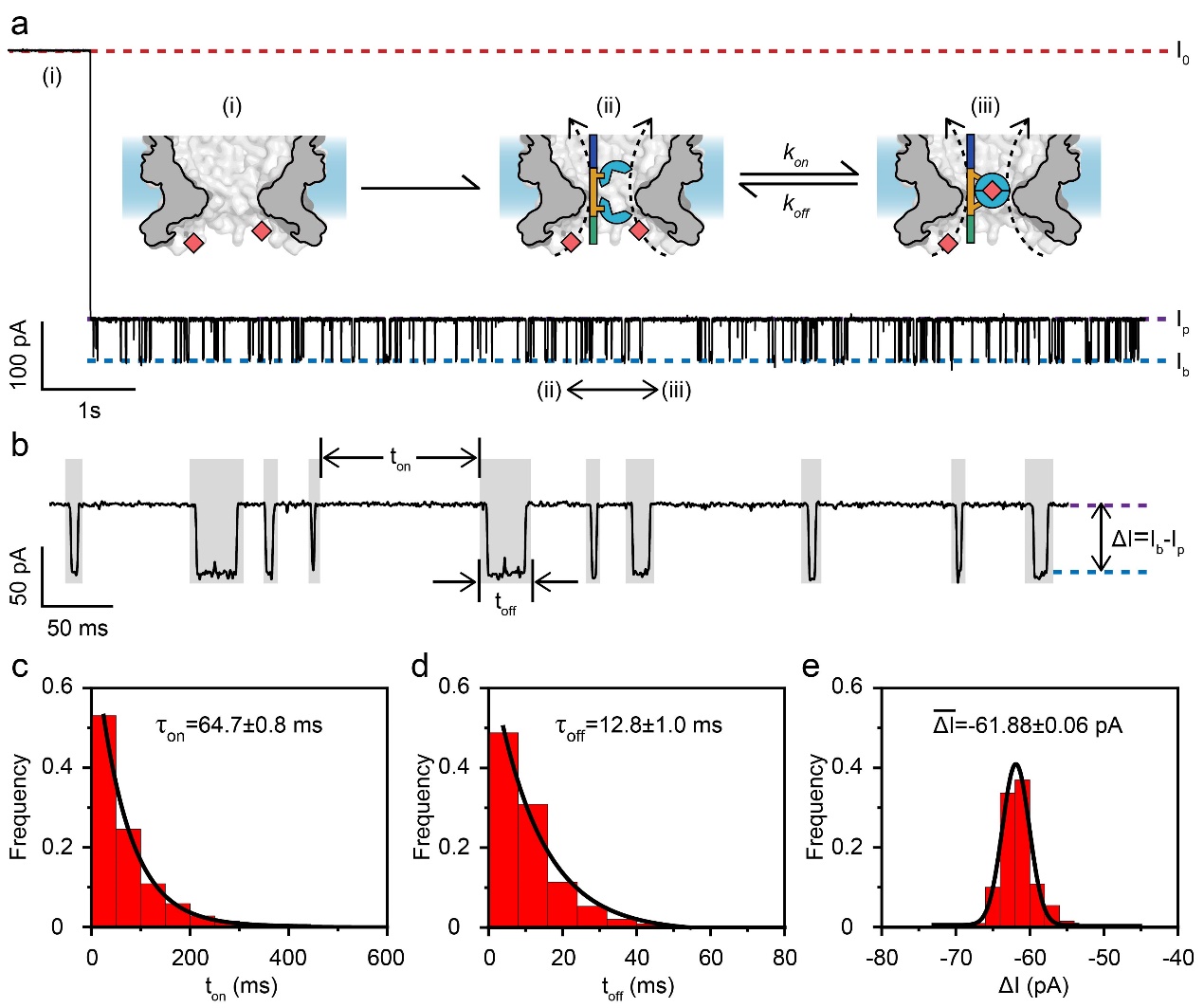
**

Supplementary fig. 4| PNRSS measurement and data analysis. **a.** A representative trace of PNRSS measurement. The cartoon describes different states of the pore during the measurement. State (i) represents an unoccupied pore, at which the measured current is the open pore current ($I_{0}$). State (ii) and (iii) represents a pore occupied with a PNRSS strand, in which the fixed reactant of the PNRSS strand is either not bound (ii) or bound (iii) with a mobile reactant. The measured current at state (ii) or (iii) is respectively defined as $I_{p}$ or $I_{b}$. **b.** A zoomed-in view of the trace containing binding events. Consecutive binding events which appear as resistive pulses (grey) are clearly observed. The event amplitude ($\Delta I$) is defined as $\Delta I$=$I_{b}-I_{p}$. Binding of a mobile reactant may generate either positive ($I_{b}{>I}_{p}$) or negative going ($I_{b}{<I}_{p}$) events, resulting in positive or negative $\Delta I$ values. The inter-event interval ($t_{on}$) and the event dwell time ($t_{off}$) are defined as described on the trace. **c-d.** The derivation of mean inter-event interval ($\tau_{on}$) **(c)** and the mean event dwell time ($\tau_{off}$) **(d)**. $t_{on}$ and $t_{off}$are fit respectively with a single exponential function $y=a*exp(-x/\tau)$, from which the mean inter-event interval ($\tau_{on}$) and the mean event dwell time ($\tau_{off}$) are derived. **e.** The derivation of mean event amplitude ($\bar{\Delta I}$). $\bar{\Delta I}$ is derived from the central position of the Gaussian fitting. The demonstrative results are from measurements as described in **Fig. 1**, carried out with the PNRSS strand 13G/14G **(Supplementary Table 1)** and Ni^2+^. Unless otherwise stated, analysis of all PNRSS measurements in this paper was carried out following the described definition.


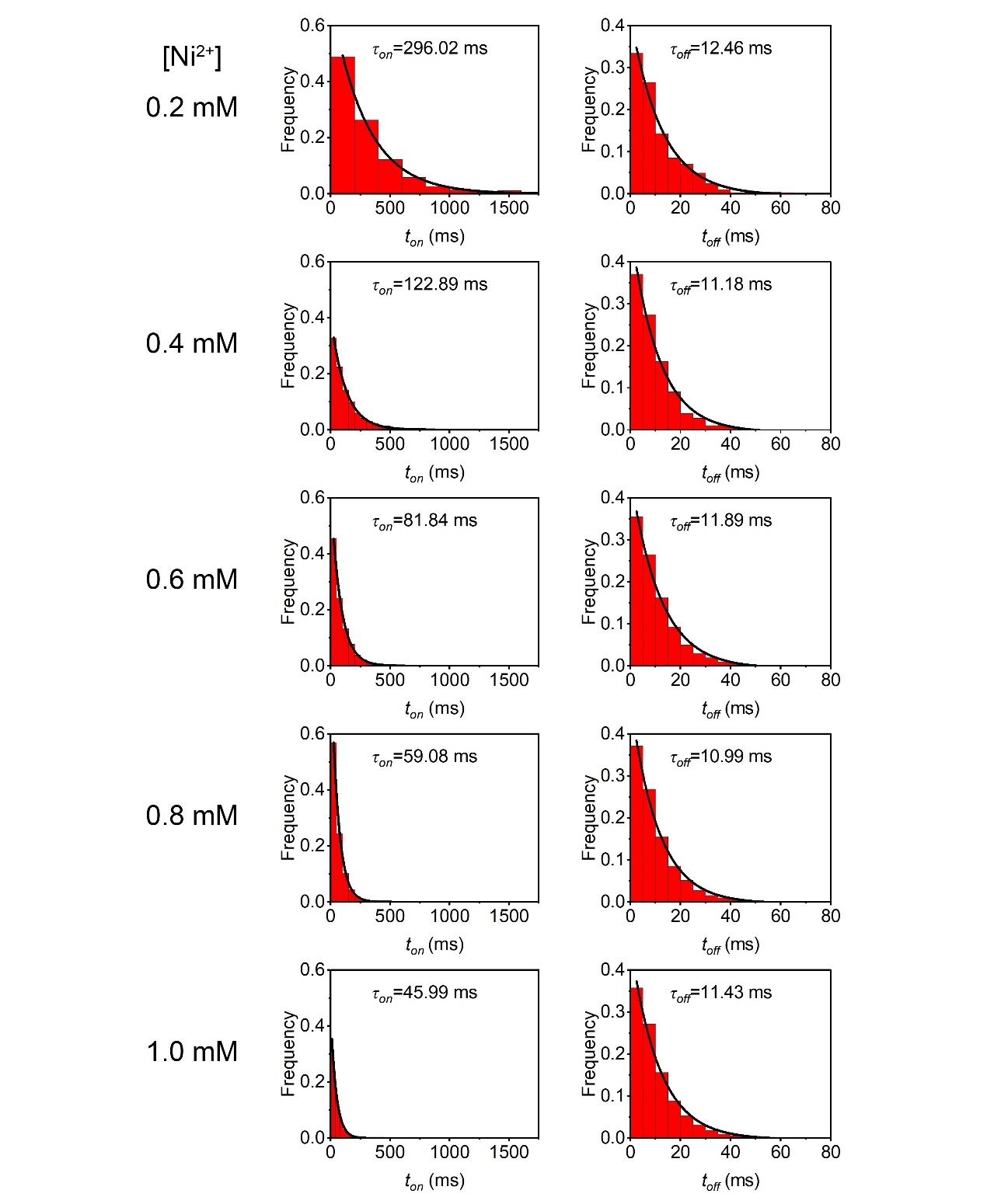


Supplementary fig. 5**|** $\boldsymbol{\tau}_{\boldsymbol{on}}$ **and** $\boldsymbol{\tau}_{\boldsymbol{off}}$ **of Ni^2+^ binding to a dual guanine reactant.** Histograms of the inter-event interval ($t_{on}$) and the event dwell time ($t_{off}$) acquired with different Ni^2+^ concentrations were presented. All histograms were respectively fit with a single exponential function $y=a*exp(-x/\tau)$, from which the mean inter-event interval ($\tau_{on}$) and the mean event dwell time ($\tau_{off}$) were derived and marked on each corresponding histogram plot. The PNRSS measurements were performed as described in **Methods**. The PNRSS strand 13G/14G **(Supplementary Table 1)** was applied. A buffer of 1.5 M KCl, 10 mM HEPES, pH 7.0 was used. A +180 mV potential was continuously applied.

**
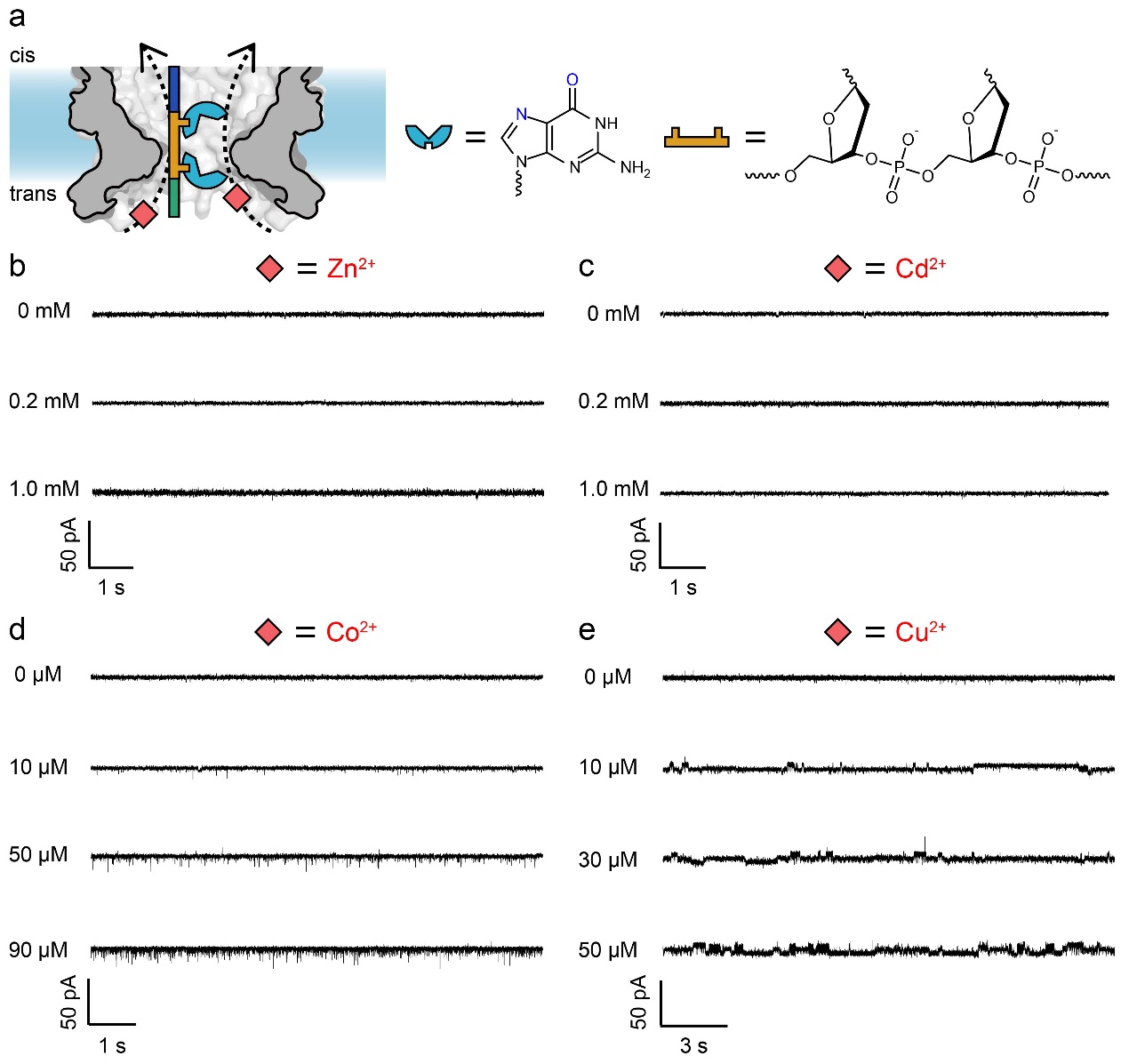
**

Supplementary fig. 6| Binding of other metal ions to a dual guanine reactant. **a.** The schematic diagram. The PNRSS measurements were similar to that described in **Fig. 1c**. A buffer of 1.5 M KCl, 10 mM HEPES, pH 7.0. was used. A +180 mV potential was continuously applied. Two neighbouring guanines on the PNRSS strand (13G/14G) serve as the fixed reactant **(Supplementary Table 1)**. Divalent ions such as Zn^2+^, Cd^2+^, Co^2+^ or Cu^2+^ serve as the mobile reactant. **b-e.** Representative traces of PNRSS measurements when Zn^2+^ **(b)**, Cd^2+^ **(c)**, Co^2+^ **(d)** or Cu^2+^ **(e)** was placed in *trans*. The final concentration of the added divalent ions is noted on the left of each corresponding trace. According to the results, Zn^2+^ **(b)** or Cd^2+^ **(c)** reports no binding to the PNRSS strand. However, Co^2+^ **(d)** or Cu^2+^ **(e)** show noticeable binding events. The above results demonstrate that binding of Co^2+^ or Cu^2+^ to a dual guanine ligand is also observable by PNRSS[^1^](#_ENREF_1). Further investigations will be carried out in a separate, follow up study.

**
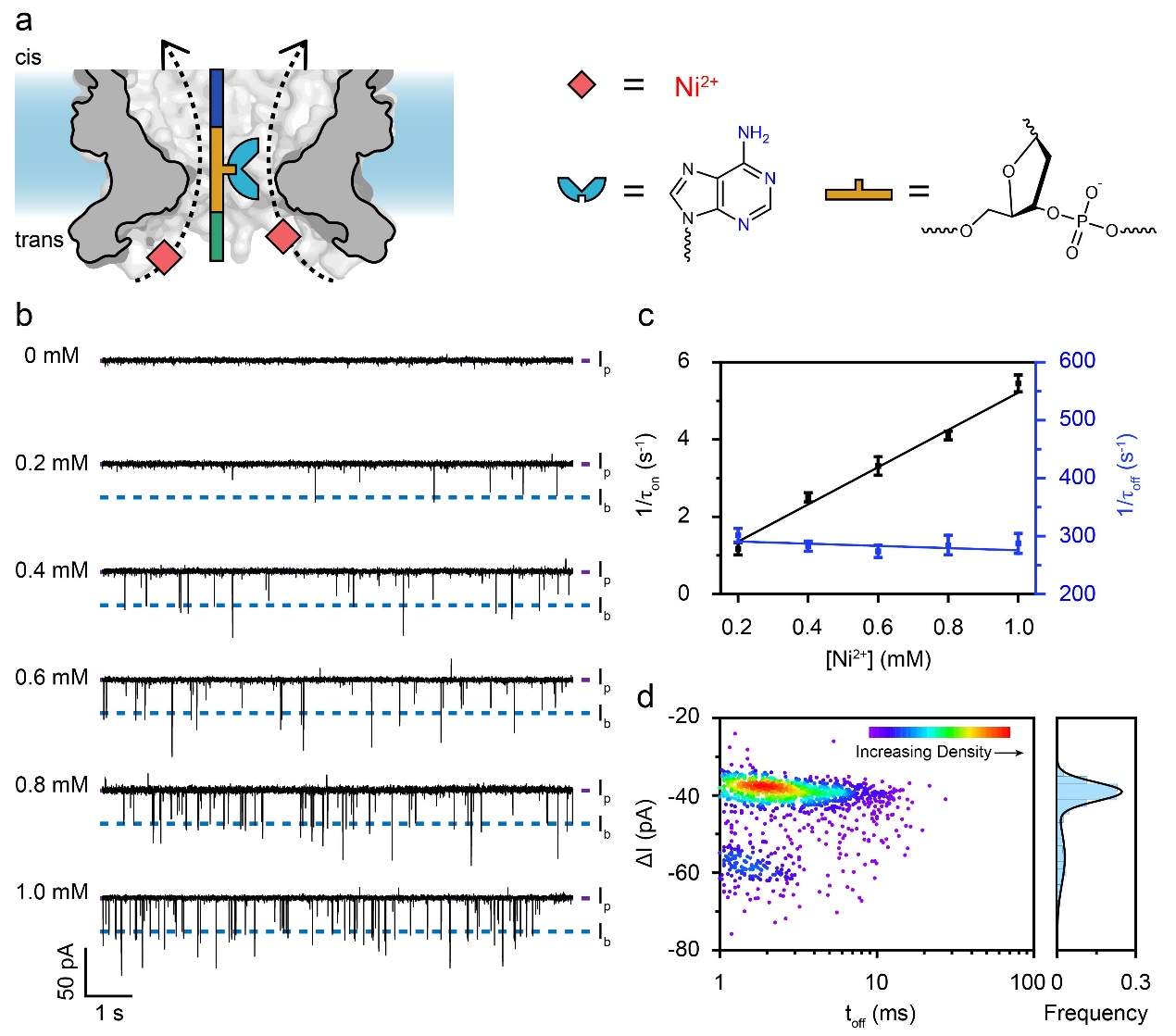
**

Supplementary fig. 7| PNRSS measurement with a sole adenine reactant. **a.** The schematic diagram. The measurements were carried out as described in **Methods**. A buffer of 1.5 M KCl, 10 mM HEPES, pH 7.0. was used. A +180 mV potential was continuously applied. The PNRSS strand 14A **(Supplementary Table 1)** was applied. 14A contains a sole adenine, serving as the fixed reactant. Ni^2+^ serves as the mobile reactant. **b.** Representative traces when Ni^2+^ was added to *trans* with varying final concentrations. The concentration of Ni^2+^ is noted on the left of each trace. Binding of Ni^2+^ to a sole adenine results in spiky, negative going events. **c.** A plot of $1/{\tau_{on}}$ or $1/{\tau_{off}}$ vs. the Ni^2+^ concentration. $1/{\tau_{on}}$ is linearly correlated to the final concentration of Ni^2+^. However, $1/{\tau_{off}}$ stays constant. Error bars=Standard Deviations (N=3). **d.** An event scatter plot of $\Delta I$ vs. $t_{off}$. The Ni^2+^ concentration was 0.8 mM. All events were extracted from a 15 min continuously recorded trace. 1137 events are included in the scatter plot. The colour scale represents the local density around each point. The density scatter plot was generated using the ggplot2 package of R. A major population measuring ~ 39 pA in $\Delta I$and a minor population measuring ~56 pA in $\Delta I$ were respectively identified. The event histogram of $\Delta I$ is attached to the right margin of the scatter plot. The two populations of $\Delta I$ are respectively Gaussian fitted and superimposed on the histogram. The above observation demonstrates that coordination interaction between Ni^2+^ and a sole adenine is observable by PNRSS. The different choice of the fixed reactant results in different binding kinetics. From a previous study, an adenine has two possible binding sites to bind a Ni^2+^, which may serve to explain the two populations of events that were observed[^2^](#_ENREF_2).


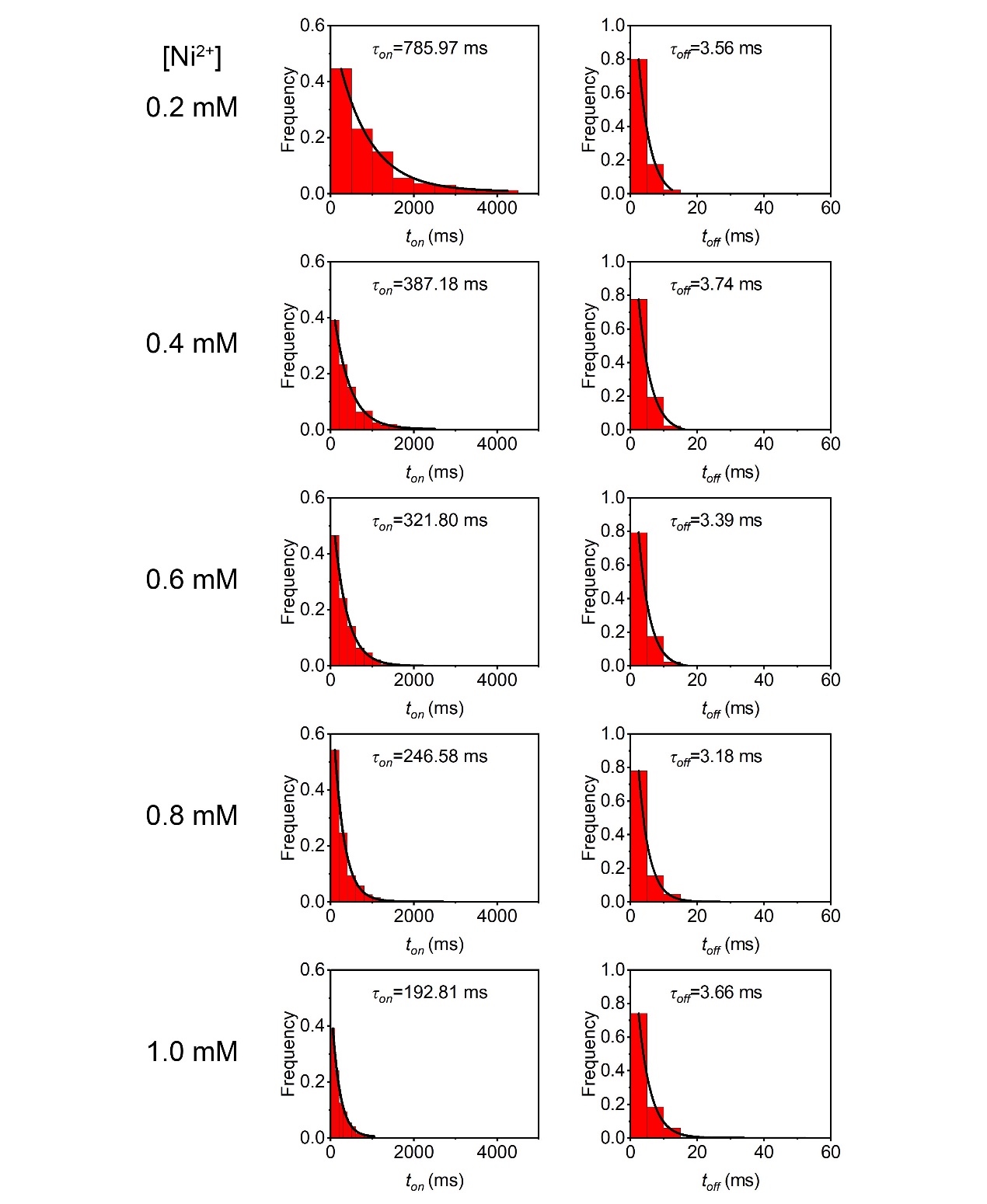


Supplementary fig. 8**|** $\boldsymbol{\tau}_{\boldsymbol{on}}$ **and** $\boldsymbol{\tau}_{\boldsymbol{off}}$ **of Ni^2+^ binding to a sole adenine reactant.** Histograms of the inter-event interval ($t_{on}$) and the event dwell time ($t_{off}$) with different Ni^2+^ concentrations are presented. All histograms were respectively fit with a single exponential function $y=a*exp(-x/\tau)$, from which the mean inter-event interval ($\tau_{on}$) and the mean event dwell time ($\tau_{off}$) were derived and marked on each corresponding histogram plot. The PNRSS measurements were performed as described in **Methods**. The PNRSS strand 14A **(Supplementary Table 1)** was applied. A buffer of 1.5 M KCl, 10 mM HEPES, pH 7.0 was used. A +180 mV potential was continuously applied.

**
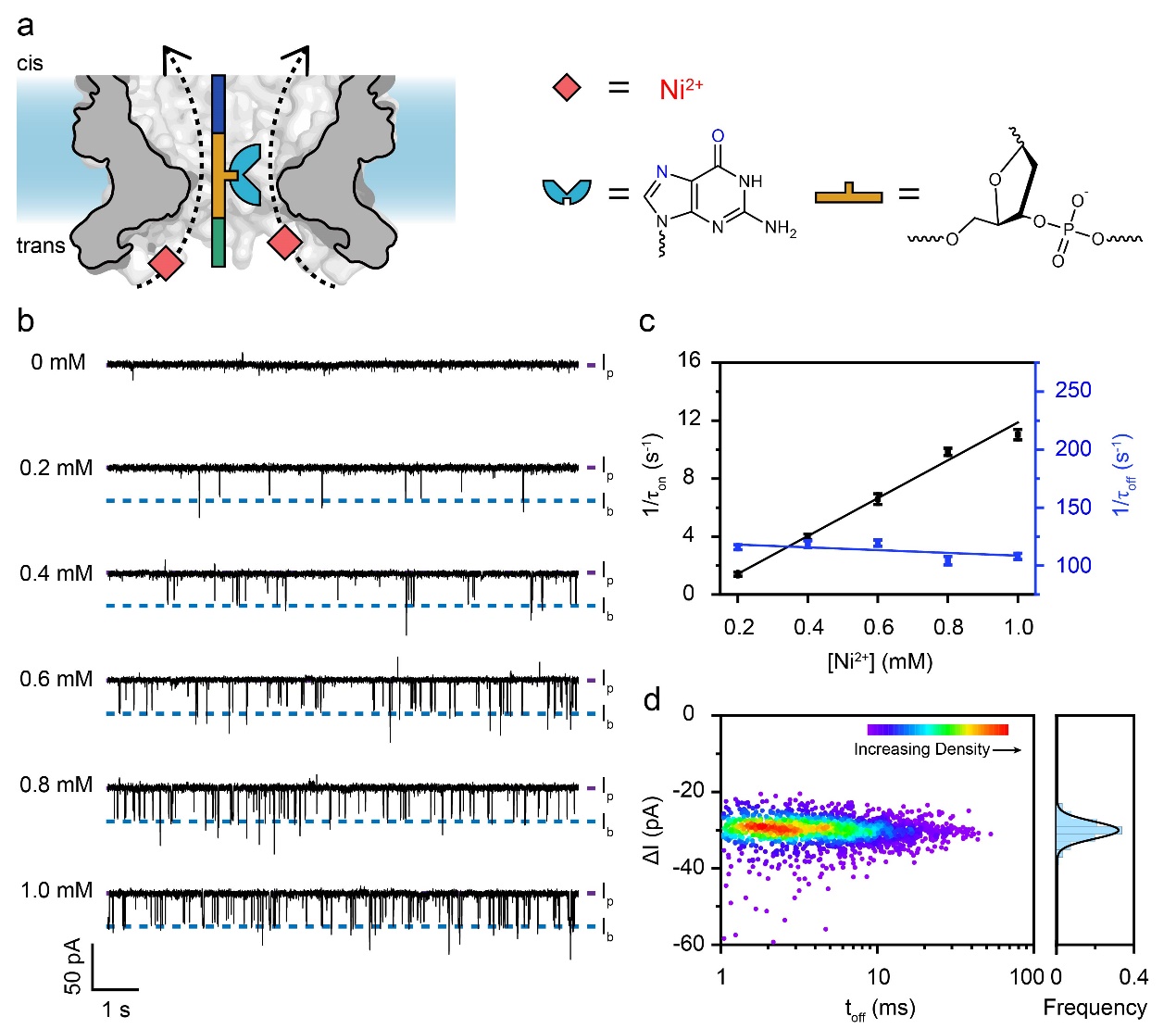
**

Supplementary fig. 9| PNRSS measurement with a sole guanine reactant. **a.** The schematic diagram. The measurements were carried out as described in **Methods**. A buffer of 1.5 M KCl, 10 mM HEPES, pH 7.0. was used. A +180 mV potential was continuously applied. The PNRSS strand 14G **(Supplementary Table 1)** was applied. 14G contains a sole guanine, serving as the fixed reactant. Ni^2+^ serves as the mobile reactant. **b.** Representative traces when Ni^2+^ was added to *trans* with varying final concentrations. The concentrations of Ni^2+^ are noted on the left of each trace. Binding events of Ni^2+^ were observed as spiky, negative going events. **c.** A plot of $1/{\tau_{on}}$ or $1/{\tau_{off}}$ vs. the Ni^2+^ concentration. $1/{\tau_{on}}$ is linearly correlated to the final concentration of Ni^2+^. However, $1/{\tau_{off}}$ stays constant. Error bars=Standard Deviations (N=3). **d.** An event scatter plot of $\Delta I$ vs. $t_{off}$. The Ni^2+^ concentration was 0.8 mM. All events were extracted from a 15 min continuously recorded trace. 1773 events are included in the scatter plot. The colour scale represents the local density around each point. The density scatter plot was generated using the ggplot2 package of R. A single population of events, measuring ~30 pA in $\Delta I$ was identified. The event histogram of $\Delta I$, which is superimposed with its Gaussian fitting result, is attached to the right margin of the scatter plot. The above observation demonstrates that coordination interaction between Ni^2+^ and a sole guanine is observable by PNRSS. The different choice of the fixed reactant results in different binding kinetics[^2^](#_ENREF_2).


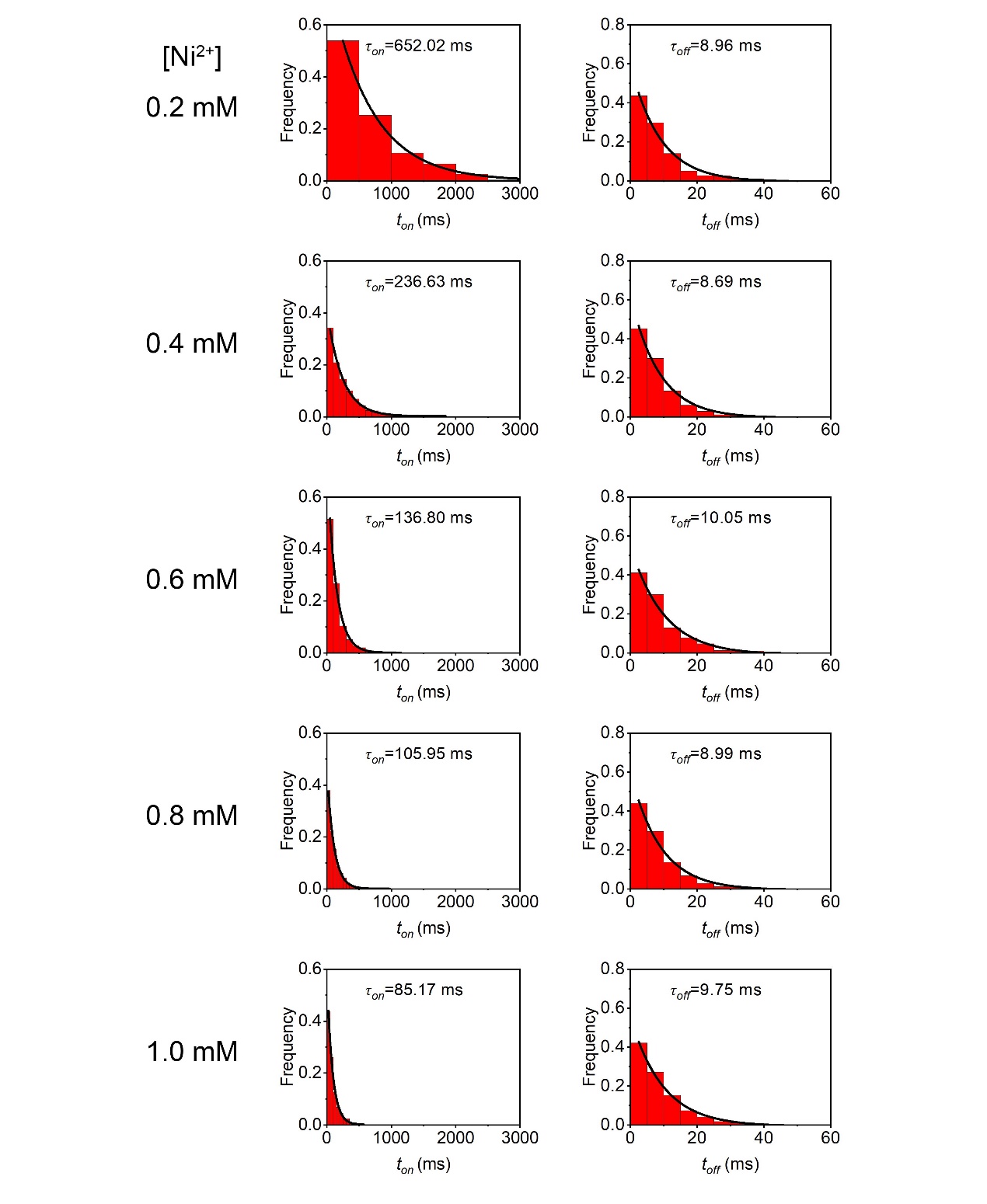


Supplementary fig. 10**|** $\boldsymbol{\tau}_{\boldsymbol{on}}$ **and** $\boldsymbol{\tau}_{\boldsymbol{off}}$ **of Ni^2+^ binding to a sole guanine reactant.** Histograms of the inter-event interval ($t_{on}$) and the event dwell time ($t_{off}$) with different Ni^2+^ concentrations are presented. All histograms were respectively fit with a single exponential function $y=a*exp(-x/\tau)$, from which the mean inter-event interval ($\tau_{on}$) and the mean event dwell time ($\tau_{off}$) were derived and marked on each corresponding histogram plot. The PNRSS measurements were performed as described in **Methods**. The PNRSS strand 14G **(Supplementary Table 1)** was applied. A buffer of 1.5 M KCl, 10 mM HEPES, pH 7.0 was used. A +180 mV potential was continuously applied.

**
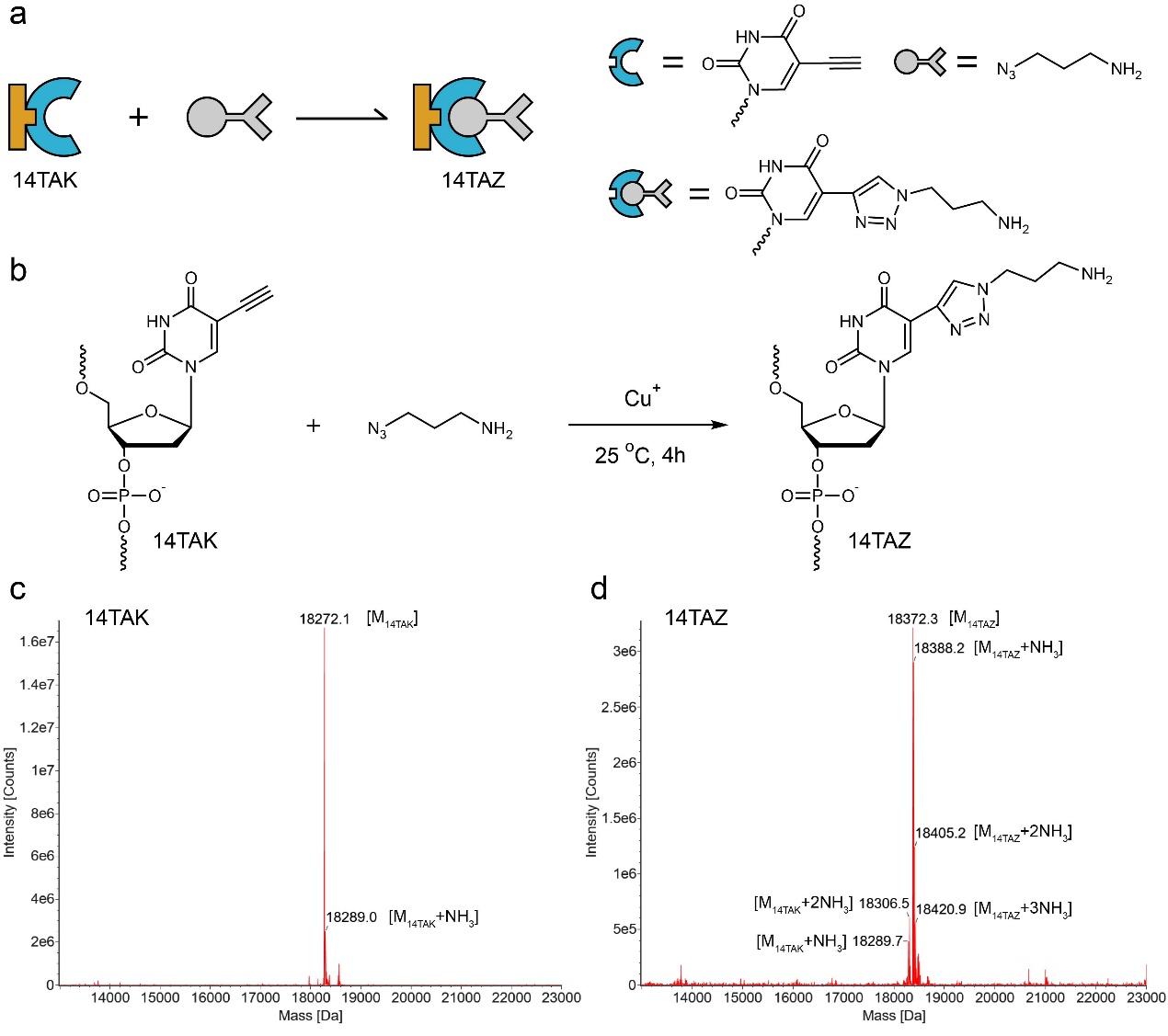
**

Supplementary fig. 11| Chemical synthesis of the PNRSS strand 14TAZ. a. The cartoon diagram of TAZ introduction to a PNRSS strand. b. The CuAAC reaction. To produce a triazole on a PNRSS strand, 3-azidopropylamine was reacted with 14TAK **(Supplementary Table 1)** by Huisgen copper (I)-catalyzed azide-alkyne 1,3-dipolarcycloaddition (CuAAC)[^3^](#_ENREF_3). Briefly, 10 μL solution of DNA 14TAK (100 μM), 6 μL acetonitrile solution of 3-azidopropylamine (330 mM), 1.5 μL copper sulfate (20 mM), 3 μL sodium ascorbate (20 mM) and 3.5 μL Milli-Q water were added to a 6 μL HEPES buffer (100 mM HEPES, pH 7.4) and shaken at 600 rpm at 25 °C for 4 h. Afterwards, 6 μL EDTA solution (100 mM) was added to the mixture to terminate the reaction. The product DNA was purified using Micro Bio-Spin 6 Columns (Bio-Rad). To confirm the success of conjugation, the purified product was analysed by liquid chromatography-mass spectrometry (Xevo G2-XS QTOF MS+Acuqity UPLC I-Class plus, Waters Corporation) equipped with an electrospray ionization (ESI) source. The product DNA is referred to as 14TAZ **(Supplementary Table 1)** and used directly in downstream PNRSS measurements. c. Mass spectrometry results of 14TAK. Calculated: 18271.0, found: 18272.1. d. Mass spectrometry results of 14TAZ. Calculated: 18371.1, found: 18372.3.

##
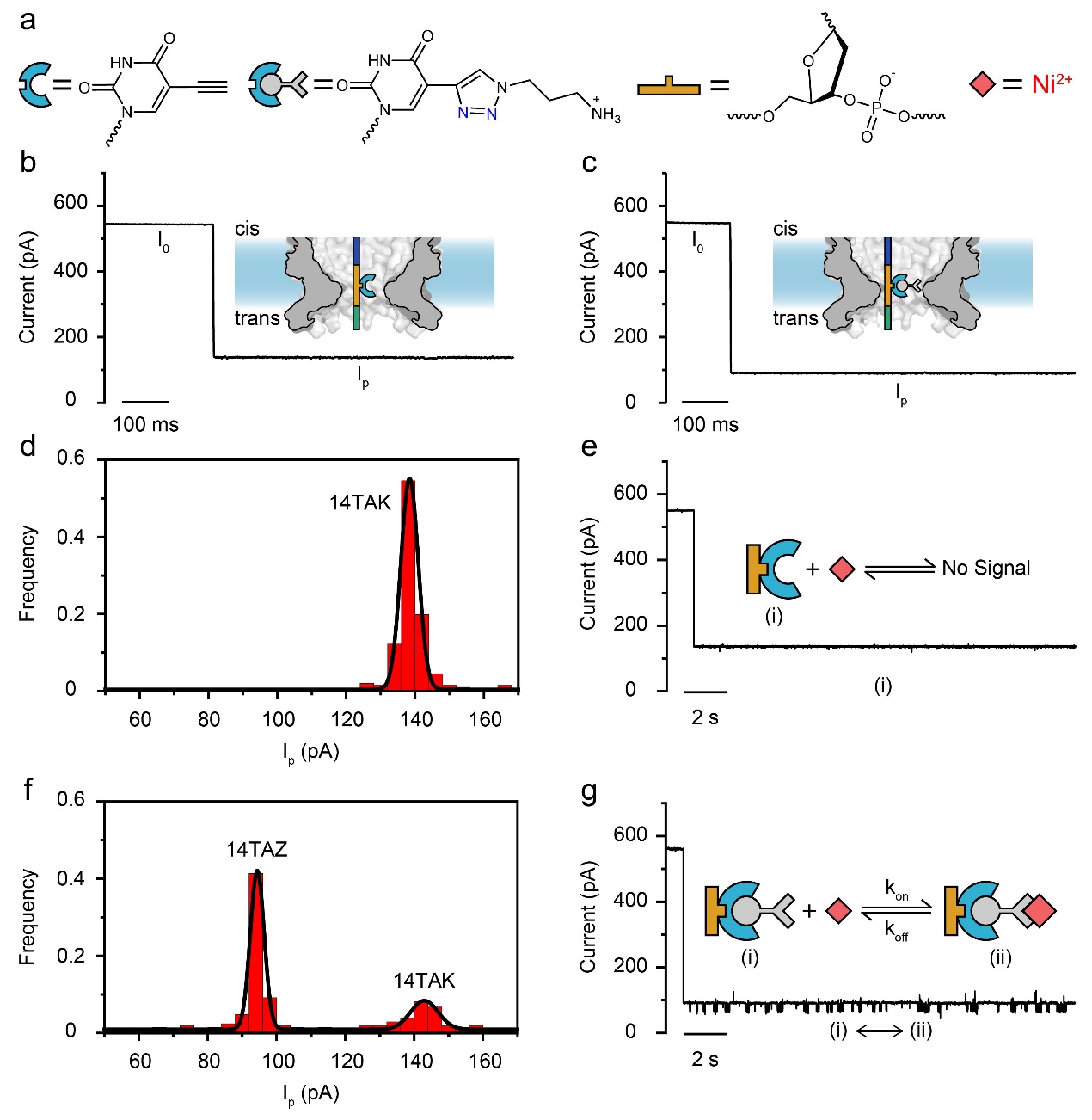


Supplementary fig. 12| Single molecule characterization of the PNRSS strand 14TAZ. **a.** The generation of a triazole and its Ni^2+^ sensing mechanism. The PNRSS strand 14TAK **(Supplementary Table 1)** contains a sole alkyne (blue arc). 3-azidopropylamine (grey symbol) was reacted with 14TAK by CuAAC **(Supplementary fig. 11)**, resulting in the production of 14TAZ, which contains a sole triazole as the fixed reactant (blue+grey). Ni^2+^, serving as the mobile reactant, is reversibly coordinated with a triazole[^4^](#_ENREF_4). **b-c.** $I_{p}$ measurement with 14TAK **(b)** or 14TAZ **(c)**. A +180 mV potential was applied. A clear difference of $I_{p}$ was observed when a 14TAK **(b)** or a 14TAZ **(c)** was captured by the pore, providing a single molecule evidence for the success of TAZ generation. This difference is also summarized in the event histogram of 14TAK **(d)** or a 14TAZ **(f)**, when static pore blockage measurements were performed. The mean blockage amplitude ($\bar{I_{p}}$) values were summarized in **Supplementary Table 5**. **e, g.** PNRSS was carried out with either 14TAK **(e)** or 14TAZ **(g)**. Ni^2+^ was applied as the mobile reactant and added to *trans* with a 1 mM final concentration. No Ni^2+^ binding events were observed with 14TAK **(e)**. However, characteristic bindings were observed with 14TAZ **(g)**, again confirming that a TAZ has been generated on the strand.

**
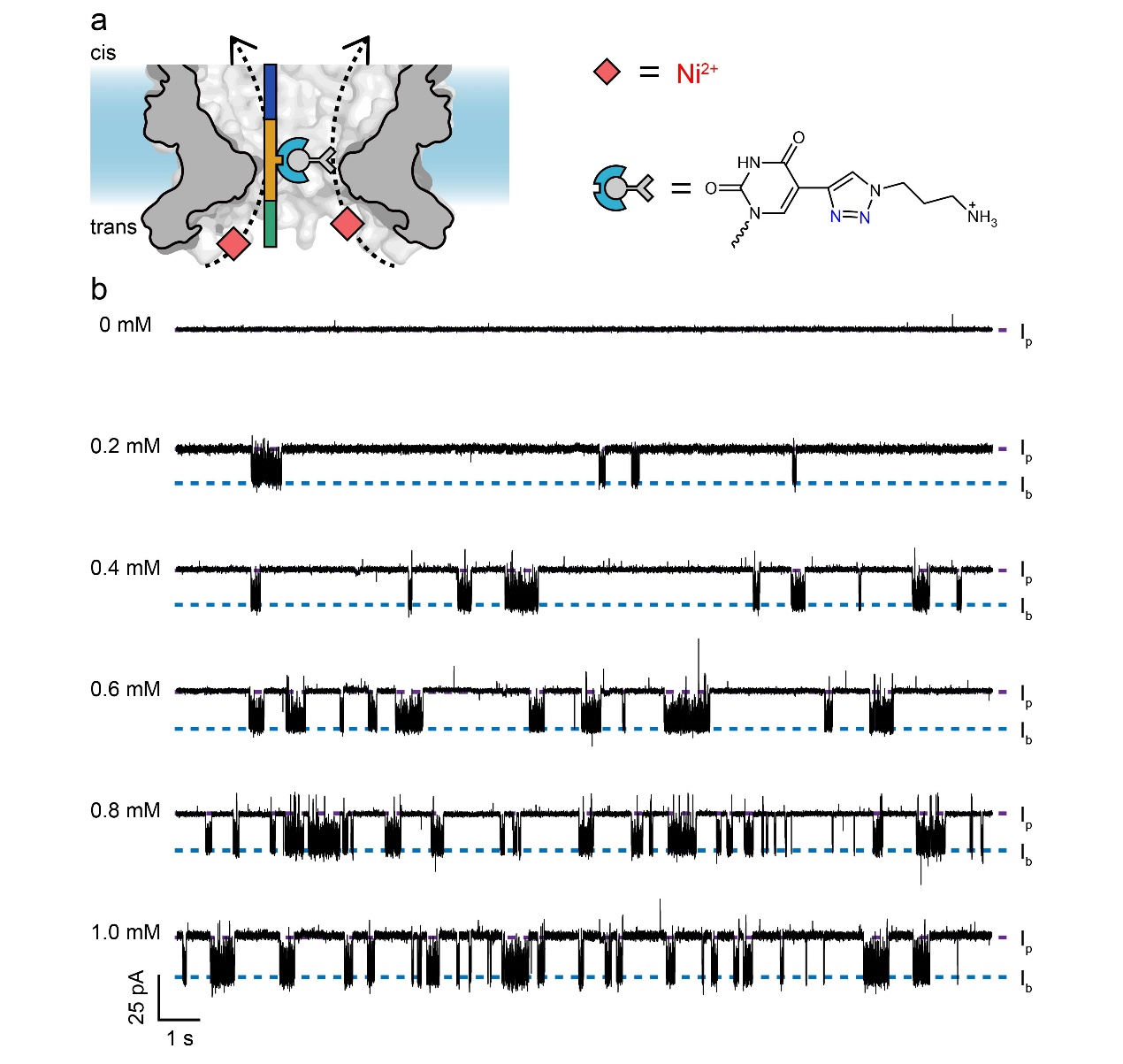
**

Supplementary fig. 13| Ni^2+^ binding to a triazole. **a.** The schematic diagram. The PNRSS measurement was carried out as described in **Fig. 2**. The PNRSS strand 14TAZ **(Supplementary Table 1)**, which contains a sole triazole, serves as the fixed reactant. Ni^2+^, which forms reversible coordination with the triazole, serves as the mobile reactant. **b.** Representative traces acquired with varying Ni^2+^ concentrations. The Ni^2+^ concentrations were adjusted between 0 and 1 mM and respectively noted on the left of each corresponding trace. Ni^2+^ binding results in negative going events ($I_{b}<I_{p}$). Characteristic noises were produced when Ni^2+^ was bound to the triazole, reporting the occurrence of this specific reaction unambiguously. The rate of event appearance increases when the Ni^2+^ concentration is raised, providing concrete evidence that the observed events result from Ni^2+^ binding.


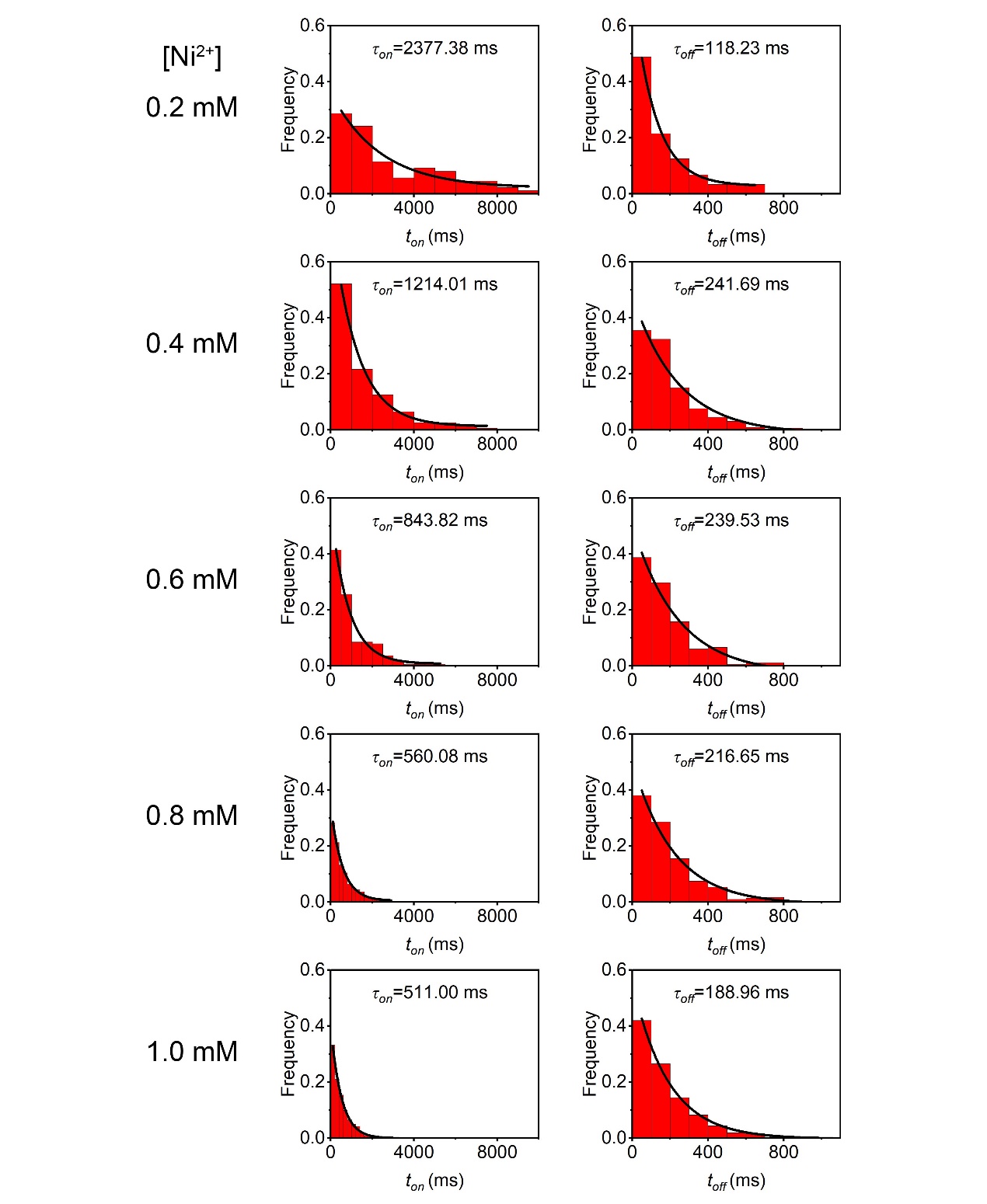


Supplementary fig. 14**|** $\boldsymbol{\tau}_{\boldsymbol{on}}$ **and** $\boldsymbol{\tau}_{\boldsymbol{off}}$ **of Ni^2+^ binding to a triazole.** Histograms of the inter-event interval ($t_{on}$) and the event dwell time ($t_{off}$) with different Ni^2+^ concentrations are presented. All histograms were respectively fit with a single exponential function $y=a*exp(-x/\tau)$, from which the mean inter-event interval ($\tau_{on}$) and the mean event dwell time ($\tau_{off}$) were derived and marked on each corresponding histogram plot. The PNRSS measurements were performed as described in **Methods**. The PNRSS strand 14TAZ **(Supplementary Table 1)** was applied. A buffer of 1.5 M KCl, 10 mM HEPES, pH 7.0 was used. A +180 mV potential was continuously applied.

**
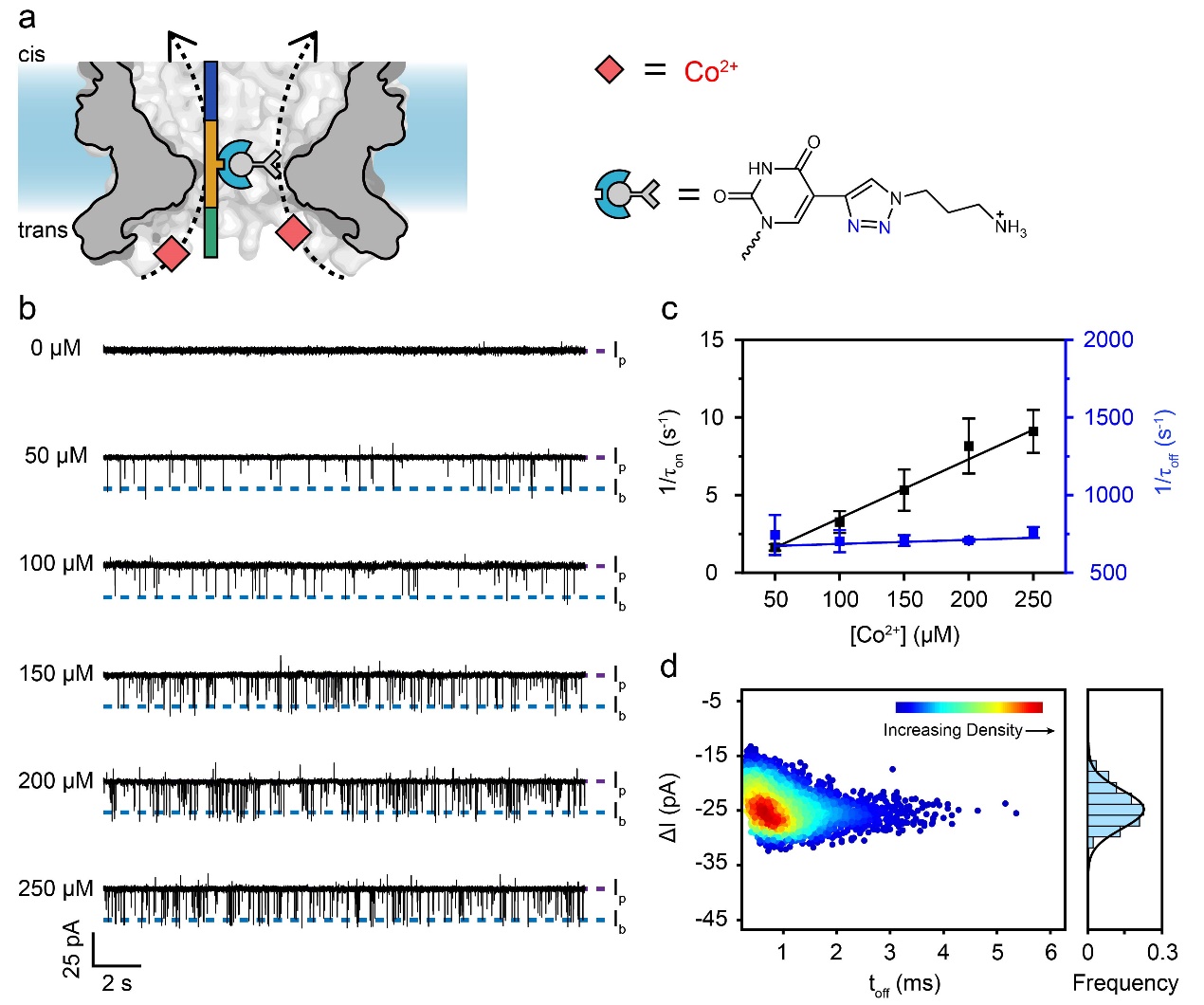
**

Supplementary fig. 15| Co^2+^ binding to a triazole. **a.** The schematic diagram. The measurements were carried out as described in **Methods**. A buffer of 1.5 M KCl, 10 mM HEPES, pH 7.0. was used. A +180 mV potential was continuously applied. The PNRSS strand 14TAZ **(Supplementary Table 1)** was applied. 14TAZ contains a sole triazole, serving as the fixed reactant. Co^2+^ serves as the mobile reactant. **b.** Representative traces when Co^2+^ was added to *trans*. The concentrations of Co^2+^ were adjusted between 0-250 µM and respectively marked on the left of each corresponding trace. Binding events of Co^2+^ were observed as spiky, negative going events. **c.** A plot of $1/{\tau_{on}}$ or $1/{\tau_{off}}$ vs. the Co^2+^ concentration. $1/{\tau_{on}}$ is linearly correlated to the final concentration of Co^2+^. However, $1/{\tau_{off}}$ stays constant. Error bars=Standard Deviations (N=3). **d.** An event scatter plot of $\Delta I$ vs. $t_{off}$. The Co^2+^ concentration was 0.2 mM. All events were extracted from a 15 min continuously recorded trace. 3632 events are included in the scatter plot. The colour scale represents the local density around each point. The density scatter plot was generated using the ggplot2 package of R. A single population of events, measuring ~-25 pA in $\Delta I$ was identified. The event histogram of $\Delta I$ is attached to the right margin of the scatter plot. The event histogram of $\Delta I$, which is superimposed with its Gaussian fitting result, is attached to the right margin of the scatter plot. The above observation demonstrates that coordination interaction between Co^2+^ and a sole triazole is observable by PNRSS. The binding characteristics is however different from that of Ni^2+^.


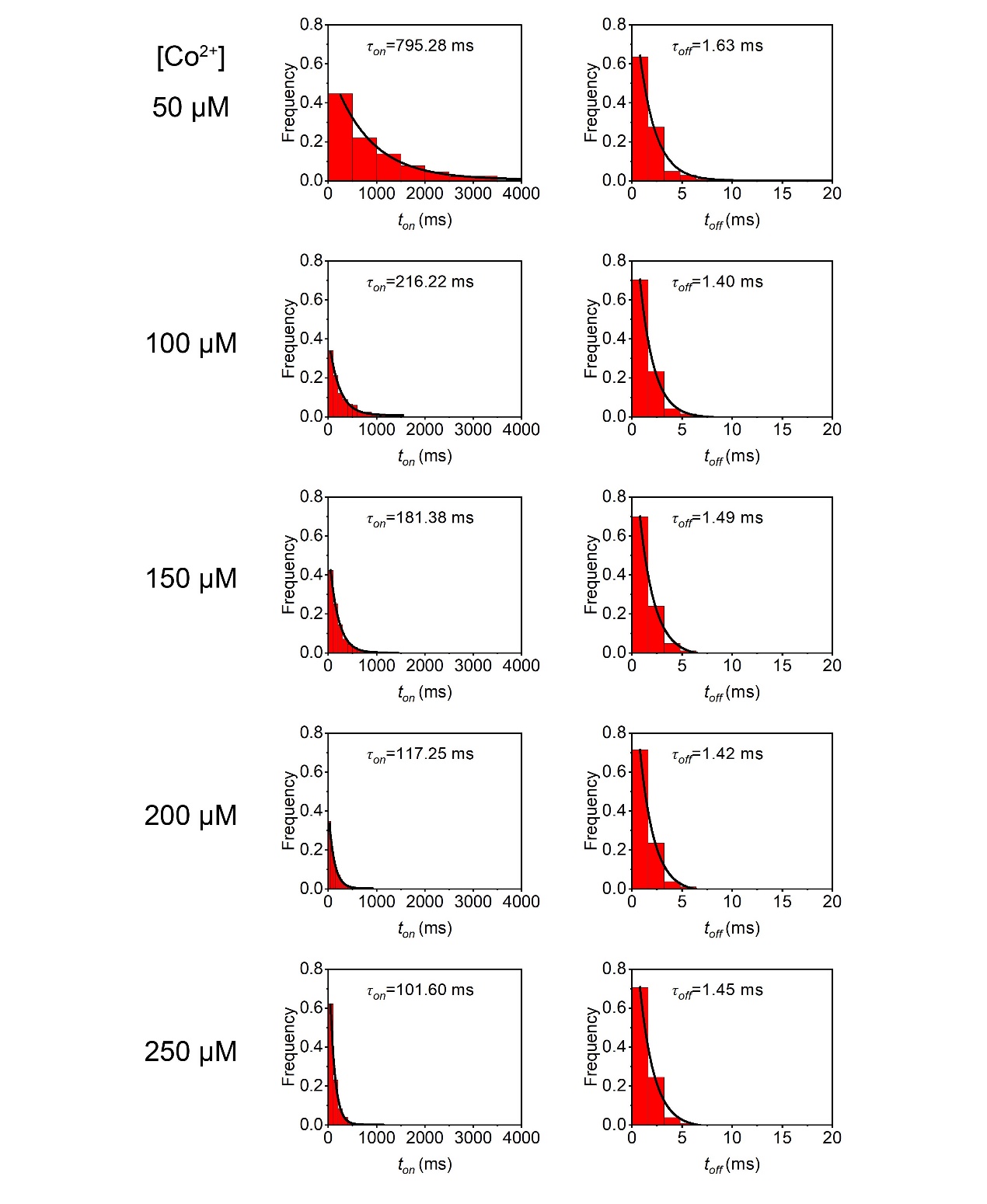


Supplementary fig. 16**|** $\boldsymbol{\tau}_{\boldsymbol{on}}$ **and** $\boldsymbol{\tau}_{\boldsymbol{off}}$ **of Co^2+^ binding to a triazole.** Histograms of the inter-event interval ($t_{on}$) and the event dwell time ($t_{off}$) with different Co^2+^ concentrations are presented. All histograms were respectively fit with a single exponential function $y=a*exp(-x/\tau)$, from which the mean inter-event interval ($\tau_{on}$) and the mean event dwell time ($\tau_{off}$) were derived and marked on each corresponding histogram plot. The PNRSS measurements were performed as described in **Methods**. The PNRSS strand 14TAZ **(Supplementary Table 1)** was applied. A buffer of 1.5 M KCl, 10 mM HEPES, pH 7.0 was used. A +180 mV potential was continuously applied.

**
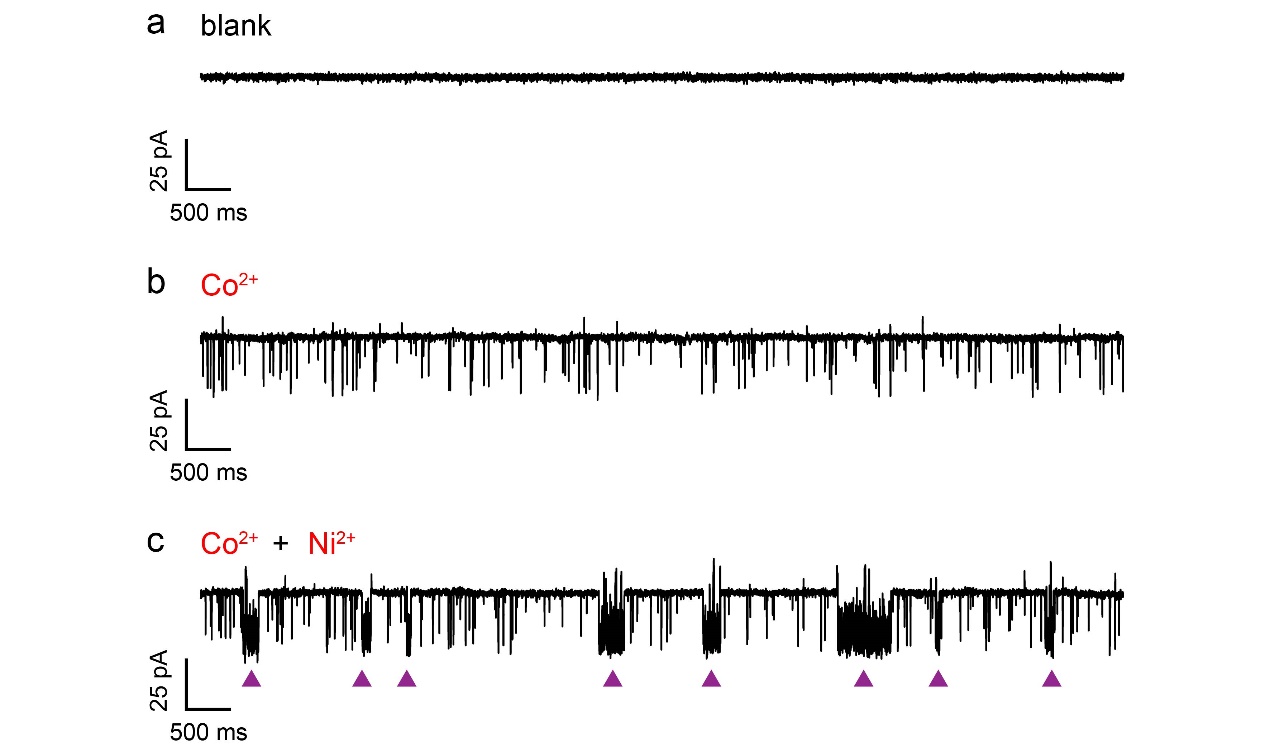
**

Supplementary fig. 17| Sequential addition of Co^2+^ and Ni^2+^ when measured by 14TAZ. The measurements were carried out as described in **Methods**. A buffer of 1.5 M KCl, 10 mM HEPES, pH 7.0. was used. A +180 mV potential was continuously applied. The PNRSS strand 14TAZ **(Supplementary Table 1)** was applied. **a.** A representative PNRSS trace when no mobile reactant was added. **b.** A representative PNRSS trace when Co^2+^ was added at a 250 µM final concentration. Only Co^2+^ binding events were observed. **c.** A representative PNRSS trace when Ni^2+^ was further added with a 400 µM final concentration. Both Co^2+^ and Ni^2+^ binding events were observed. The unique event noises generated by Ni^2+^ provide unambiguous evidence for event recognition.

**
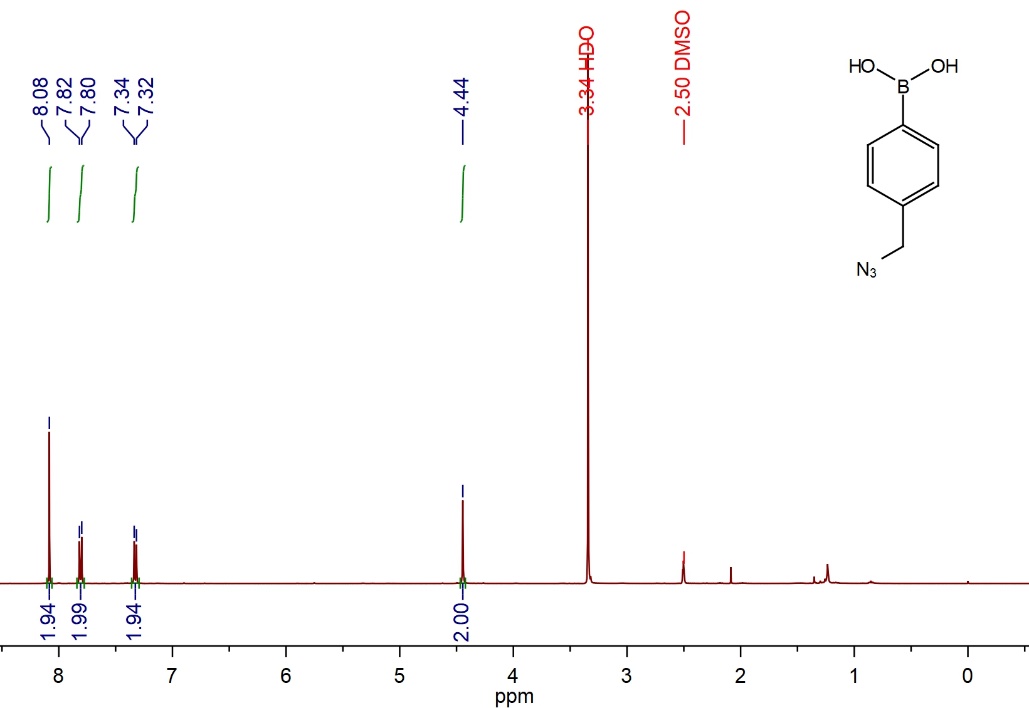
**

Supplementary fig. **18| ^1^H NMR spectrum of 4-(Azidomethyl) benzeneboronic acid.** ^1^H NMR (BRUKER AVANCE III, 400 MHz, 298K, DMSO-d6) δ 8.08 (s, 2H), 7.81 (d, *J* = 8.0 Hz, 2H), 7.33 (d, *J* = 8.0 Hz, 2H), 4.44 (s, 2H). [^5^](#_ENREF_5)


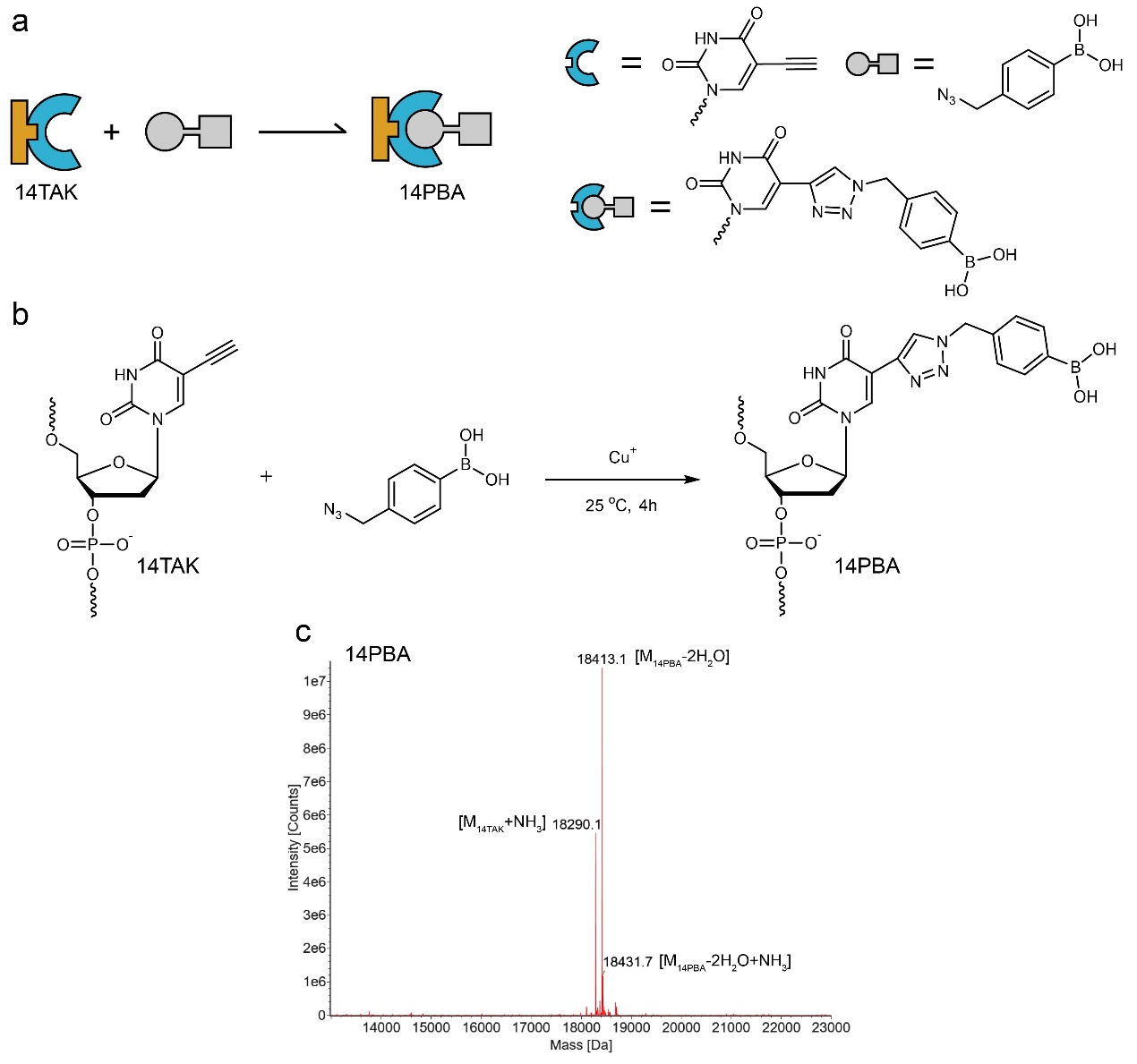


Supplementary fig. 19| Chemical synthesis of the PNRSS strand 14PBA. a. The cartoon diagram of PBA introduction. b. The reaction. To produce a PBA on a PNRSS strand, 4-(azidomethyl) benzeneboronic acid was reacted with 14TAK **(Supplementary Table 1)** by Huisgen copper (I)-catalyzed azide-alkyne 1,3-dipolarcycloaddition (CuAAC)[^6^](#_ENREF_6). Briefly, 10 μL solution of DNA 14TAK (100 μM), 6 μL 4-(azidomethyl) benzeneboronic acid (dissolved in MeCN, 200 mM), 1.5 μL copper sulfate (20 mM), 3 μL sodium ascorbate (20 mM) and 3.5 μL Milli-Q water were added to a 6 μL HEPES buffer (100 mM HEPES, pH 7.4) and shaken at 600 rpm at 25 °C for 4 h. Subsequently, 6 μL EDTA solution (100 mM) was added to the mixture to terminate the reaction. The product DNA was purified using Micro Bio-Spin 6 Columns (Bio-Rad). To confirm the success of conjugation, the purified product was analysed by liquid chromatography-mass spectrometry (Xevo G2-XS QTOF MS+Acuqity UPLC I-Class plus, Waters Corporation) equipped with an electrospray ionization (ESI) source. This functionalized DNA is referred to as 14PBA **(Supplementary Table 1)** and used directly in downstream PNRSS measurements. c. Mass spectrometry results of 14PBA. For [14PBA], the calculated mass: 18448.0. For [14PBA-2H_2_O], the calculated mass: 18412.0, found: 18413.1. During mass spectrometry measurement, every boronic acid loses 2 H_2_O, likely resulted from a strong intramolecular interaction of boronic acid with the DNA phosphate backbone, a phenomenon also reported in literature[^7^](#_ENREF_7).

**
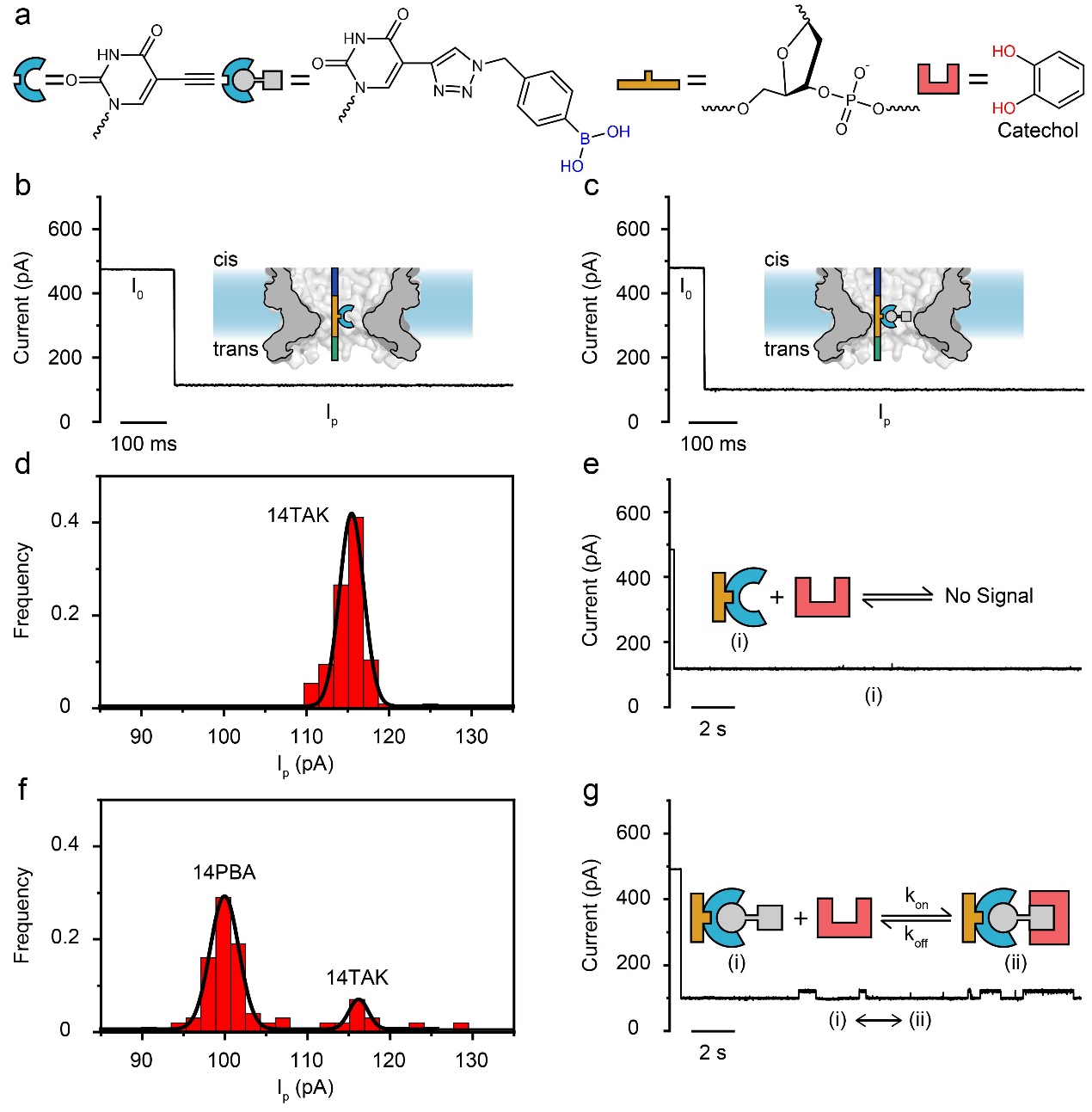
**

Supplementary fig. 20| Single molecule characterization of the PNRSS strand 14PBA. **a.** Introduction of phenylboronic acid (PBA) and its catechol sensing mechanism. The PNRSS strand 14TAK **(Supplementary Table 1)** contains a sole alkyne (blue arc). 4-(azidomethyl) benzeneboronic acid (grey symbol) was reacted with 14TAK by CuAAC, resulting in the production of 14PBA, which contains a sole PBA as the fixed reactant. Details of synthesis and characterization of 14PBA are provided in **Supplementary figs. 18-19**. Catechol, which forms reversible interactions with a PBA, is applied as the mobile reactant. **b-c.** The $I_{p}$ measurements with 14TAK **(b)** or 14PBA **(c)**. A +160 mV potential was applied. An obvious difference of $I_{p}$ was observed when a 14TAK **(b)** or a 14PBA **(c)** was captured by the pore, providing a single molecule evidence for the success of PBA conjugation. This difference is also summarized in the event histogram of $I_{p}$ when 14TAK **(d)** or 14PBA **(f)** was measured during static pore blockage measurements. The mean blockage amplitude ($\bar{I_{p}}$) values are summarized in **Supplementary Table 7**. **e, g.** PNRSS was carried out with either 14TAK **(e)** or 14PBA **(g)**. Catechol was applied as the mobile reactant and added to *trans* with a 500 µM final concentration. No catechol binding events were observed with 14TAK **(e)**. However, characteristic bindings were observed with 14PBA **(g)**, again confirming that a PBA has been successfully conjugated to the strand.


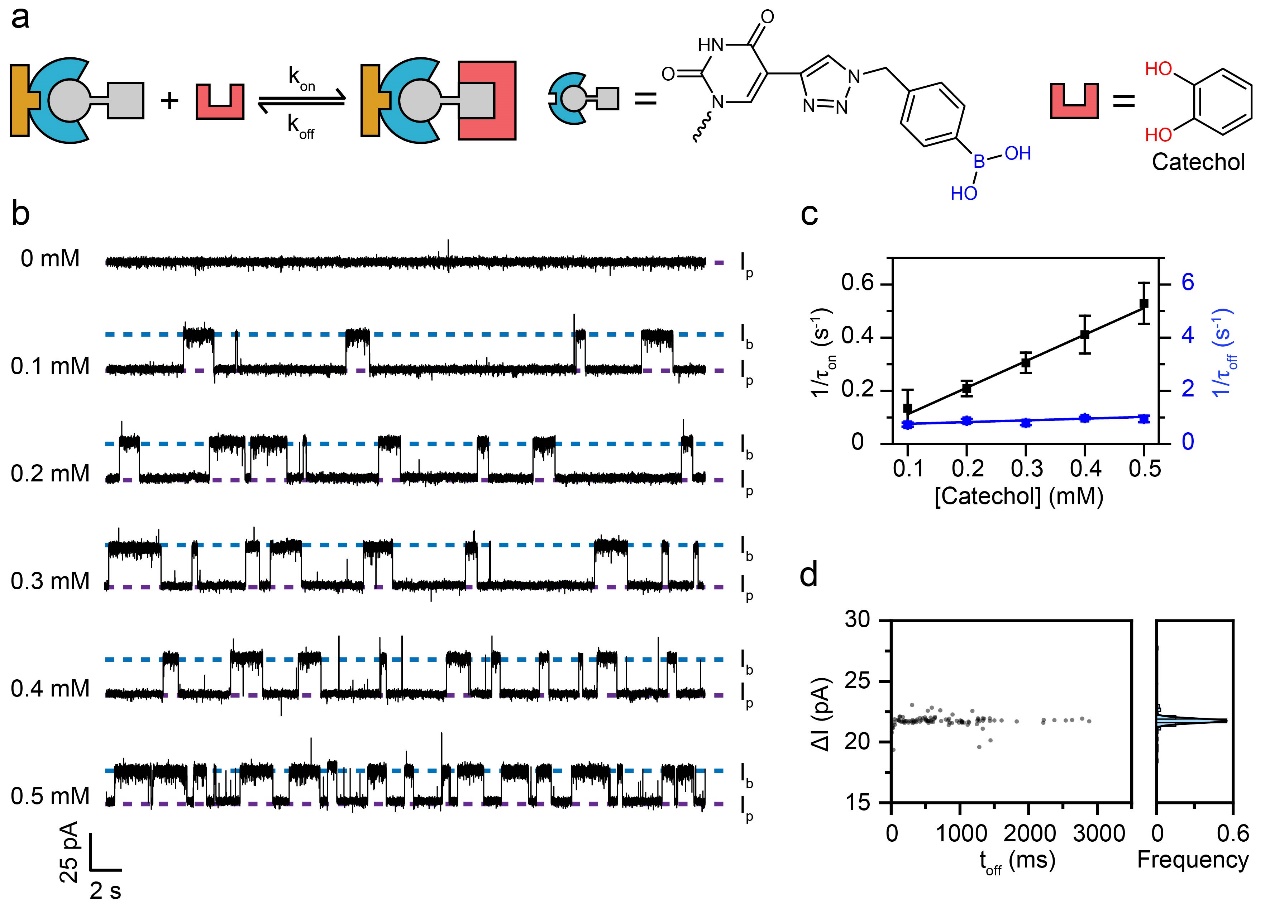
 Supplementary fig. 21| Catechol binding to a PBA. **a.** The schematic diagram. The PNRSS strand 14PBA **(Supplementary Table 1)** contains a sole PBA at site 14, capable of binding catechol [^8^](#_ENREF_8), as illustrated by the cartoon diagram. **b.** Representative traces containing catechol binding events. A buffer of 1.5 M KCl, 10 mM HEPES, pH 8.0 was used. A +160 mV potential was continuously applied. Catechol was added to *trans* with a final concentration of 0-0.5 mM, marked on the left of each corresponding trace. Binding of catechol results in positive going events ($I_{b}>I_{p}$). The rate of event appearance is increases when the catechol concentration is raised. **c.** Concentration dependence. The reciprocal of inter-event interval $1/{\tau_{on}}$ and the reciprocal of dwell time $1/{\tau_{off}}$ is plotted against the final concentration of catechol in *trans*. $1/{\tau_{on}}$ shows a linear correlation with the concentration of catechol**.** $1/{\tau_{off}}$ stays constant. Error bars=Standard Deviations (N=3). **d.** Scatter plot of $\Delta I$ vs. $t_{off}$. 100 events are included in the scatter plot. The histogram of $\Delta I$, superimposed with its Gaussian fitting result, is plotted to the right of the scatter plot. The catechol concentration was 400 µM. The events were extracted from a 15 min continuously recorded trace.


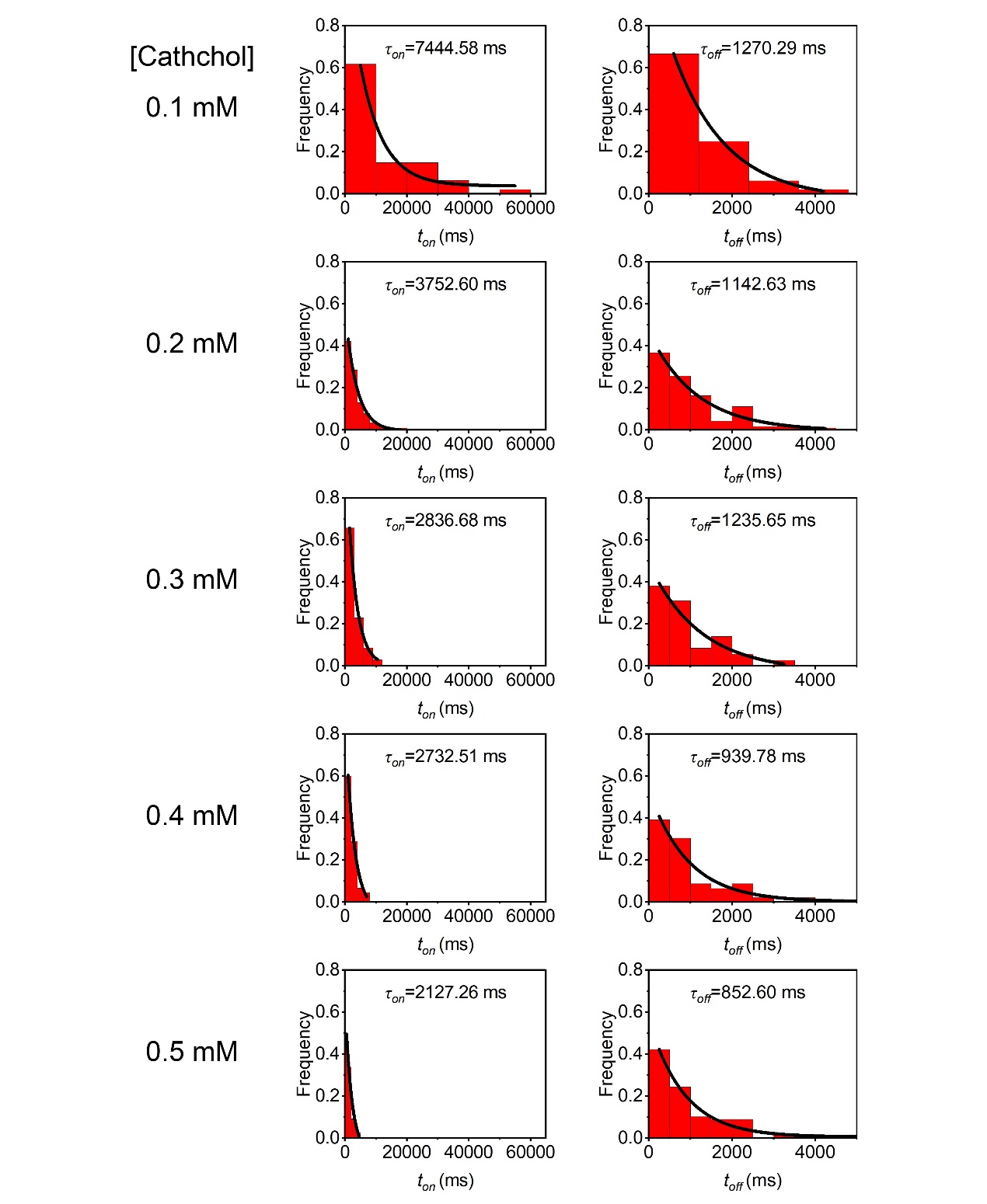


Supplementary fig. 22**|** $\boldsymbol{\tau}_{\boldsymbol{on}}$ **and** $\boldsymbol{\tau}_{\boldsymbol{off}}$ **of catechol binding to a PBA.** Histograms of the inter-event interval ($t_{on}$) and the event dwell time ($t_{off}$) with different catechol concentrations are presented. Catechol was added to *trans* with a final concentration of 0.1-0.5 mM. The applied concentration is marked on the left of each corresponding Histogram. All histograms were respectively fit with a single exponential function $y=a*exp(-x/\tau)$, from which the mean inter-event interval ($\tau_{on}$) and the mean event dwell time ($\tau_{off}$) were derived and marked on each corresponding histogram plot. The PNRSS measurements were performed as described in **Methods**. The PNRSS strand 14PBA **(Supplementary Table 1)** was applied. A buffer of 1.5 M KCl, 10 mM HEPES, pH 8.0 was used. A +160 mV potential was continuously applied.

**
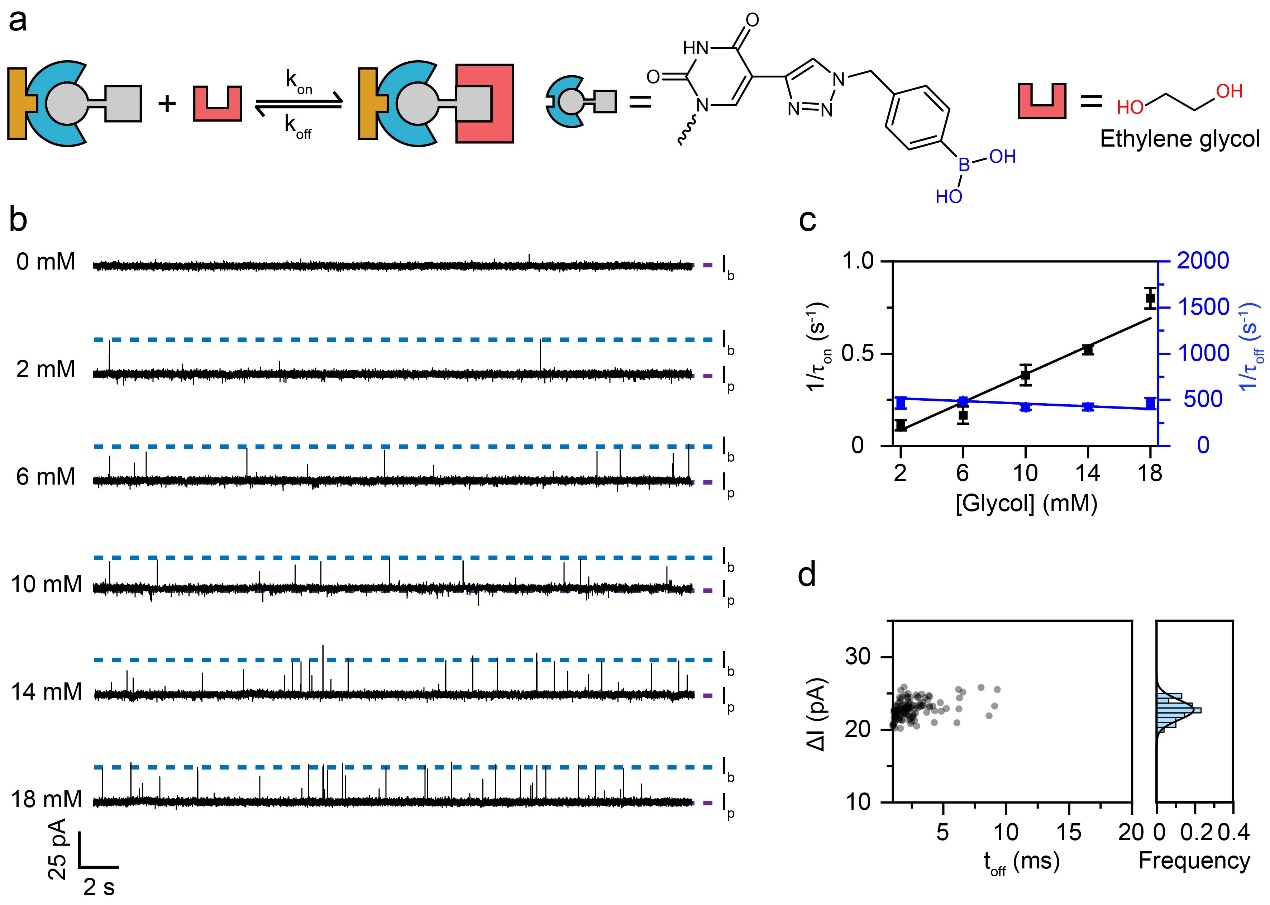
**

Supplementary fig. 23| Ethylene glycol binding to a PBA. **a.** The schematic diagram. The PNRSS strand 14PBA **(Supplementary Table 1)** contains a sole PBA at site 14, capable of binding ethylene glycol [^9^](#_ENREF_9), as illustrated by the cartoon diagram. **b.** Representative traces containing ethylene glycol binding events. A buffer of 1.5 M KCl, 10 mM HEPES, pH 8.0 was used. A +160 mV potential was continuously applied. Ethylene glycol was added to *trans* with a final concentration of 0-18 mM, marked on the left of each corresponding trace. The rate of event appearance increases when the ethylene glycol concentration is raised. **c.** Concentration dependence. The reciprocal of inter-event interval ($1/{\tau_{on}}$) and the reciprocal of dwell time ($1/{\tau_{off}}$) is plotted against the final concentration of ethylene glycol. $1/{\tau_{on}}$ demonstrates a linear correlation with the concentration of ethylene glycol**.** $1/{\tau_{off}}$ remains constant. Error bars=Standard Deviations (N=3). **d.** Scatter plot of $\Delta I$ vs. $t_{off}$. 135 events are included in the scatter plot. The histogram of $\Delta I$, superimposed with its Gaussian fitting result, is plotted to the right of the scatter plot. The ethylene glycol concentration was 14 mM. The events were extracted from a 15 min continuously recorded trace.


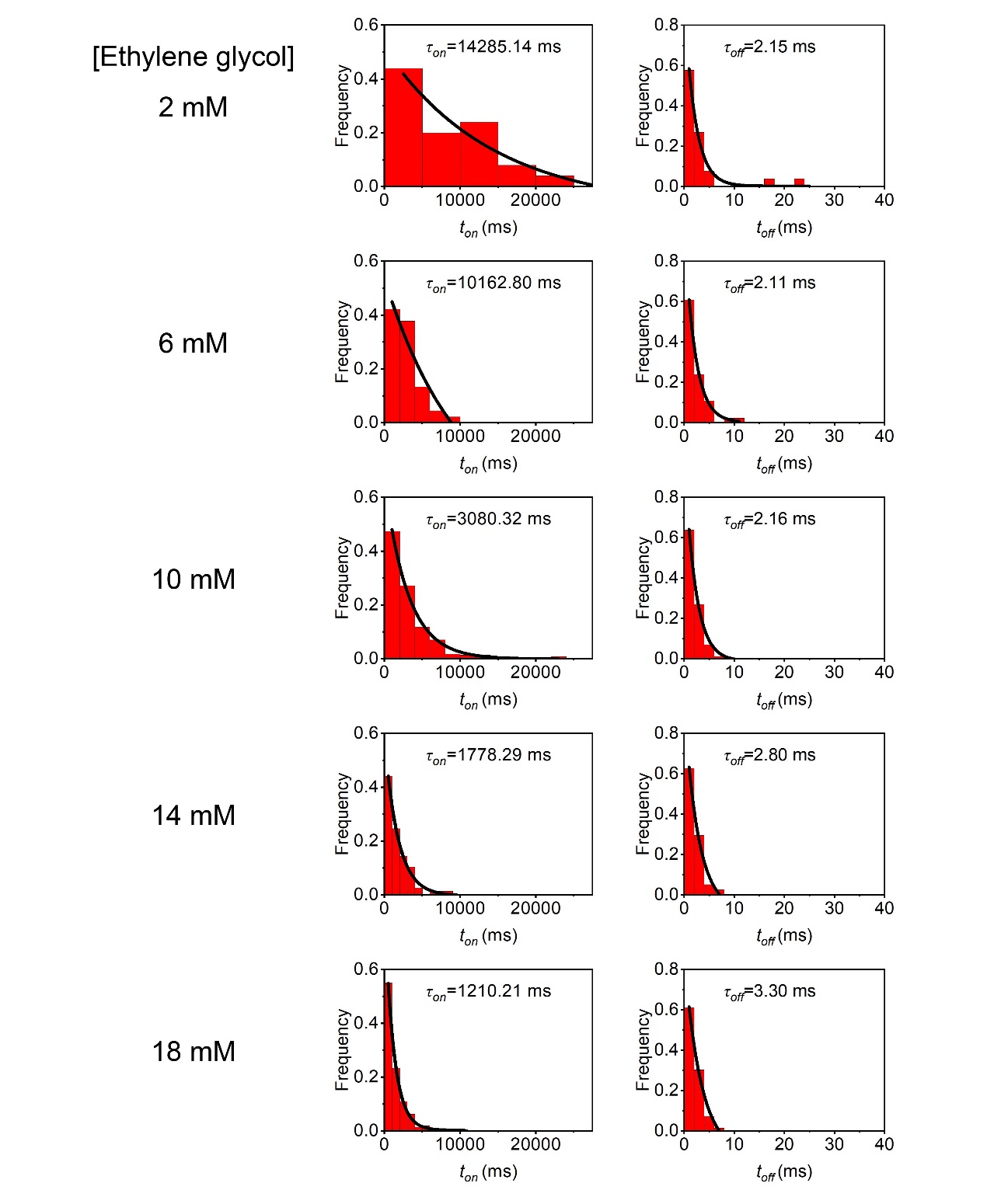


Supplementary fig. 24**|** $\boldsymbol{\tau}_{\boldsymbol{on}}$ **and** $\boldsymbol{\tau}_{\boldsymbol{off}}$ **of ethylene glycol binding to a PBA.** Histograms of the inter-event interval ($t_{on}$) and the event dwell time ($t_{off}$) with different ethylene glycol concentrations are presented. Ethylene glycol was added to *trans* with a final concentration of 2-18 mM. The applied concentration is marked on the left of each corresponding histogram plot. All histograms were respectively fit with a single exponential function $y=a*exp(-x/\tau)$, from which the mean inter-event interval ($\tau_{on}$) and the mean event dwell time ($\tau_{off}$) were derived and marked on each corresponding histogram plot. The PNRSS measurements were performed as described in **Methods**. The PNRSS strand 14PBA **(Supplementary Table 1)** was applied. A buffer of 1.5 M KCl, 10 mM HEPES, pH 8.0 was used. A +160 mV potential was continuously applied.

**
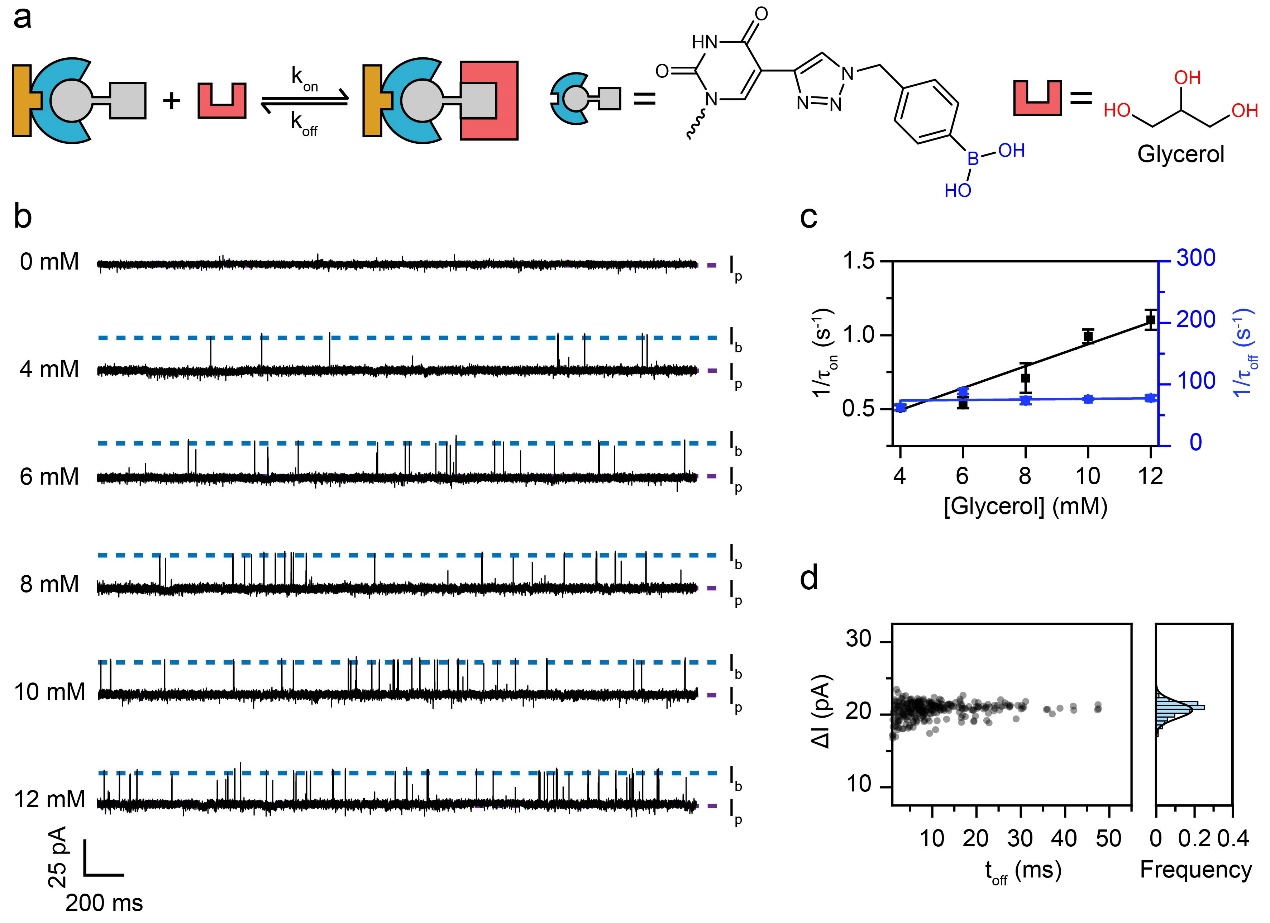
**

Supplementary fig. 25| Glycerol binding to a PBA. **a.** The schematic diagram. The PNRSS strand 14PBA **(Supplementary Table 1)** contains a sole PBA at site 14, capable of binding glycerol[^9^](#_ENREF_9), as illustrated by the cartoon diagram. **b.** Representative traces containing glycerol binding events. A buffer of 1.5 M KCl, 10 mM HEPES, pH 8.0 was used. A +160 mV potential was continuously applied. Glycerol was added to *trans* with a final concentration of 0-12 mM, marked on the left of each corresponding trace. The rate of event appearance increases when the glycerol concentration is raised. **c.** Concentration dependence. The reciprocal of inter-event interval ($1/{\tau_{on}}$) and the reciprocal of dwell time ($1/{\tau_{off}}$) is plotted against the final concentration of glycerol. $1/{\tau_{on}}$ demonstrates a linear correlation with the concentration of glycerol**.** $1/{\tau_{off}}$ remains constant. Error bars=Standard Deviations (N=3). **d.** Scatter plot of $\Delta I$ vs. $t_{off}$. 267 events are included in the scatter plot. The histogram of $\Delta I$, superimposed with its Gaussian fitting result, is plotted to the right of the scatter plot. The glycerol concentration was 10 mM. The events were extracted from a 15 min continuously recorded trace.


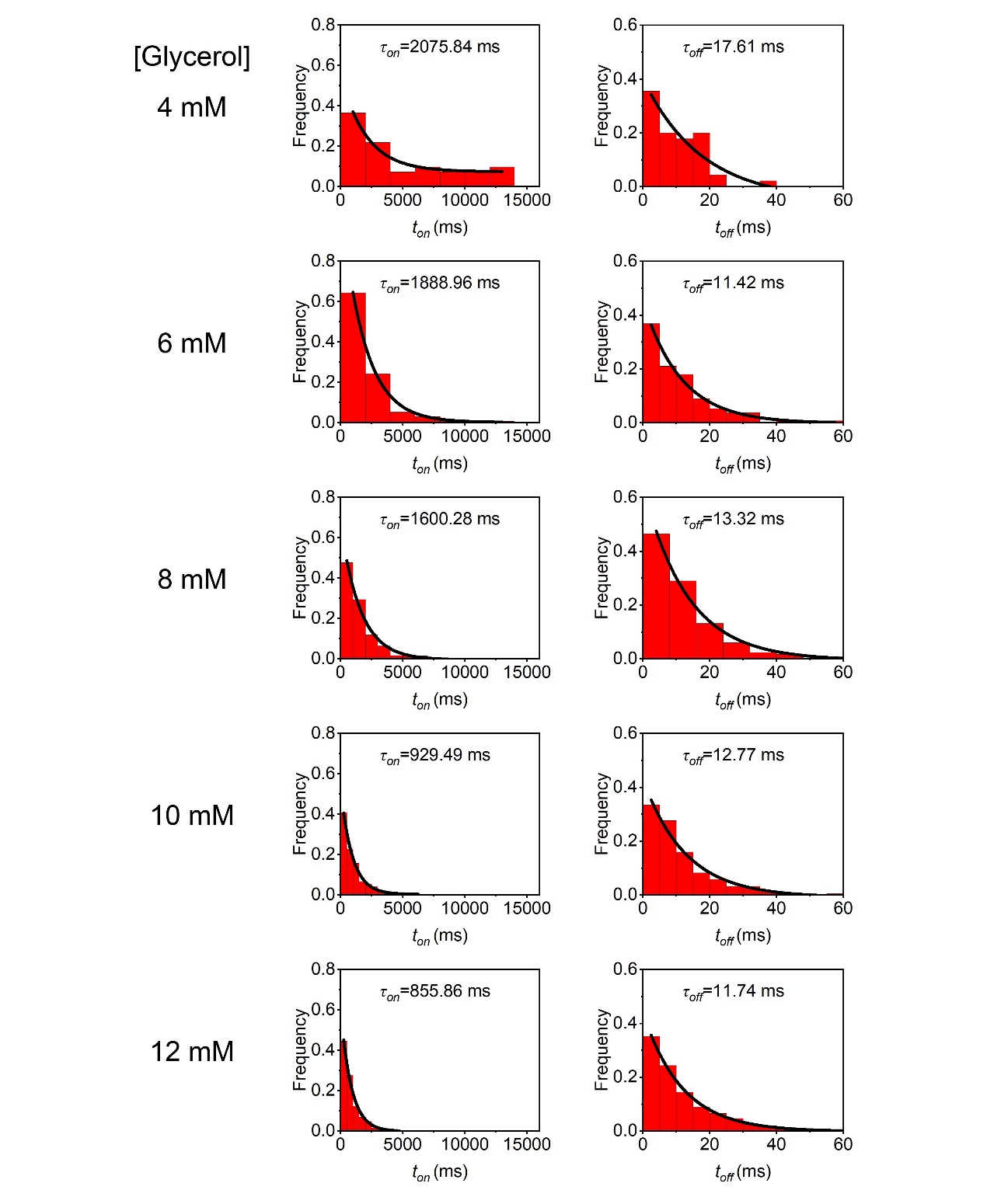


Supplementary fig. 26**|** $\boldsymbol{\tau}_{\boldsymbol{on}}$ **and** $\boldsymbol{\tau}_{\boldsymbol{off}}$ **of glycerol binding to a PBA.** Histograms of the inter-event interval ($t_{on}$) and the event dwell time ($t_{off}$) with different glycerol concentrations are presented. Glycerol was added to *trans* with a final concentration of 4-12 mM. The applied concentration is marked on the left of each corresponding histogram plot. All histograms were respectively fit with a single exponential function $y=a*exp(-x/\tau)$, from which the mean inter-event interval ($\tau_{on}$) and the mean event dwell time ($\tau_{off}$) were derived and marked on each corresponding histogram plot. The PNRSS measurements were performed as described in **Methods**. The PNRSS strand 14PBA **(Supplementary Table 1)** was applied. A buffer of 1.5 M KCl, 10 mM HEPES, pH 8.0 was used. A +160 mV potential was continuously applied.

**
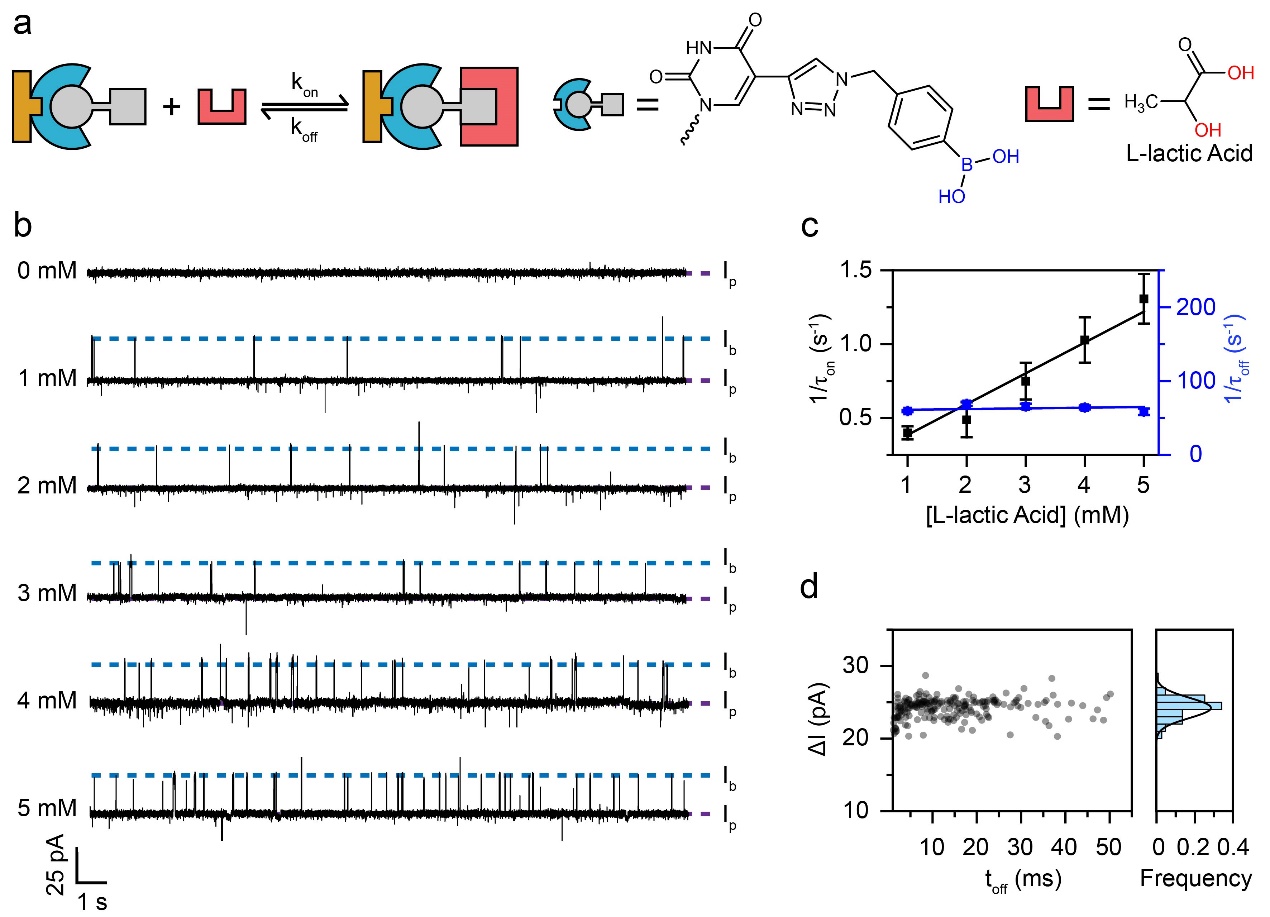
**

Supplementary fig. 27| L-lactic acid binding to a phenylboronic acid (PBA) reactant. **a.** The schematic diagram. The PNRSS strand 14PBA **(Supplementary Table 1)** contains a sole PBA at site 14, capable of binding L-lactic acid [^8^](#_ENREF_8), as illustrated by the cartoon diagram. **b.** Representative traces containing L-lactic acid binding events. A buffer of 1.5 M KCl, 10 mM HEPES, pH 8.0 was used. A +160 mV potential was continuously applied. L-lactic acid was added to *trans* with a final concentration of 0-5 mM, marked on the left of each corresponding trace. The rate of event appearance increases when the L-lactic concentration is raised. **c.** Concentration dependence. The reciprocal of inter-event interval ($1/{\tau_{on}}$) and the reciprocal of dwell time ($1/{\tau_{off}}$) is plotted against the final concentration of L-lactic acid. $1/{\tau_{on}}$ demonstrates a linear correlation with the concentration of L-lactic acid**.** $1/{\tau_{off}}$ stays constant. Error bars=Standard Deviations (N=3). **d.** Scatter plot of $\Delta I$ vs. $t_{off}$. 213 events are included in the scatter plot. The histogram of $\Delta I$, superimposed with its Gaussian fitting result, is plotted to the right of the scatter plot. The lactic acid concentration was 4 mM. The events were extracted from a 15 min continuously recorded trace.


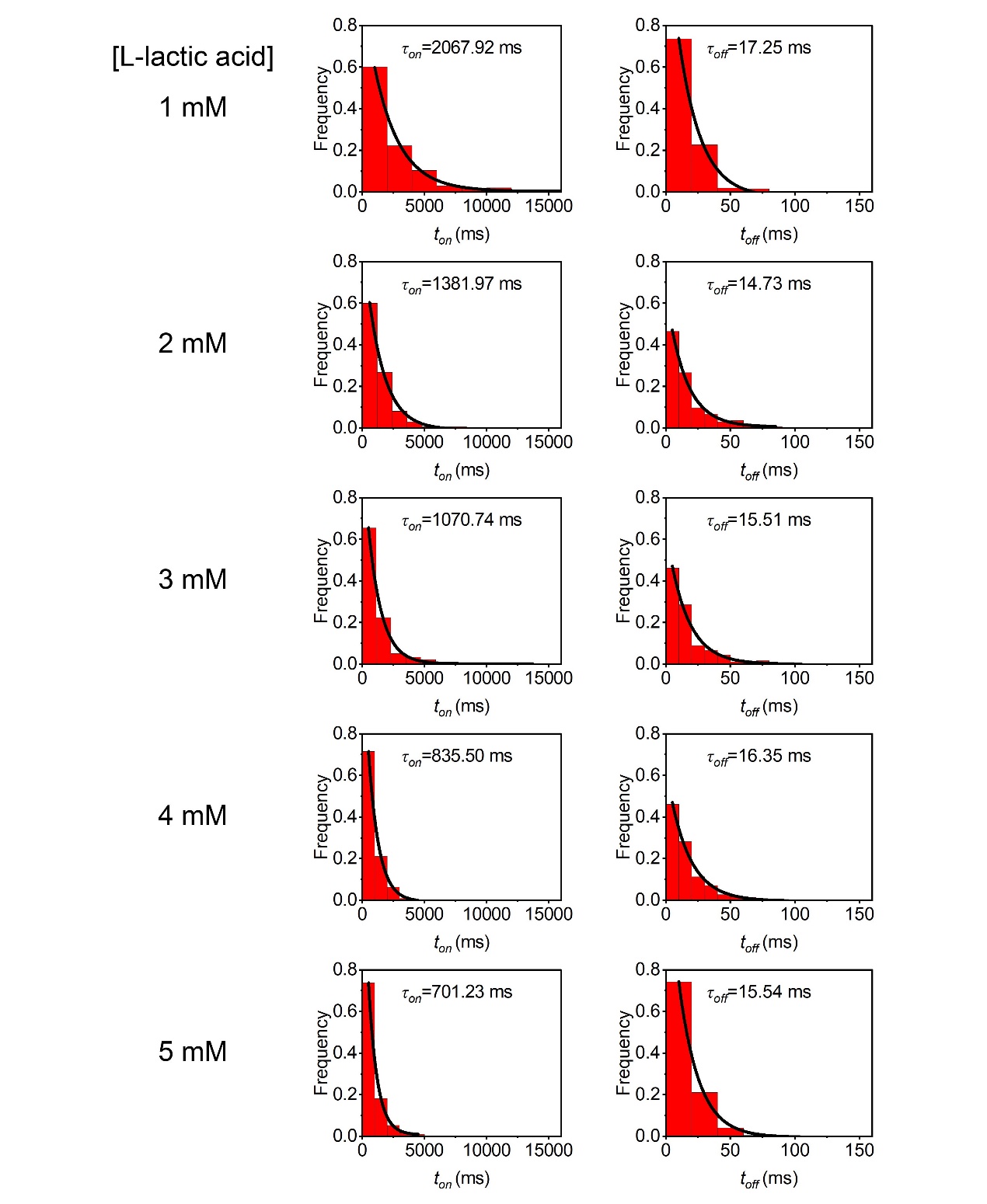


Supplementary fig. 28**|** $\boldsymbol{\tau}_{\boldsymbol{on}}$ **and** $\boldsymbol{\tau}_{\boldsymbol{off}}$ **of L-lactic acid binding to a PBA.** Histograms of the inter-event interval ($t_{on}$) and the event dwell time ($t_{off}$) with different L-lactic acid concentrations are presented. L-lactic acid was added to *trans* with a final concentration of 1-5 mM. The applied concentration is marked on the left of each corresponding histogram plot. All histograms were respectively fit with a single exponential function $y=a*exp(-x/\tau)$, from which the mean inter-event interval ($\tau_{on}$) and the mean event dwell time ($\tau_{off}$) were derived and marked on each corresponding histogram plot. The PNRSS measurements were performed as described in **Methods**. The PNRSS strand 14PBA **(Supplementary Table 1)** was applied. A buffer of 1.5 M KCl, 10 mM HEPES, pH 8.0 was used. A +160 mV potential was continuously applied.

**
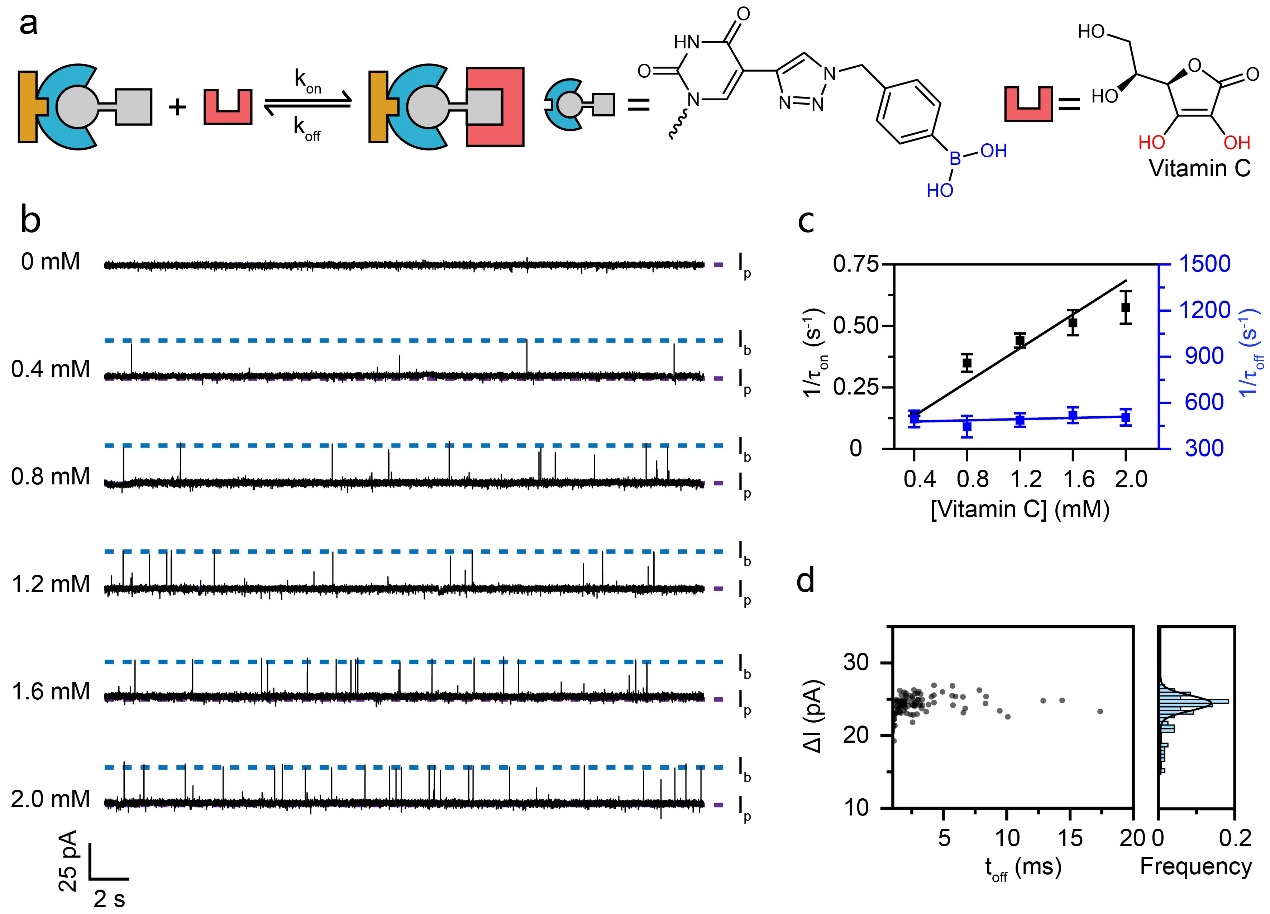
**

Supplementary fig. 29| Vitamin C binding to a PBA. **a.** The schematic diagram. The PNRSS strand 14PBA **(Supplementary Table 1)** contains a sole PBA at site 14, capable of binding vitamin C [^10^](#_ENREF_10), as illustrated by the cartoon diagram. **b.** Representative traces containing vitamin C binding events. A buffer of 1.5 M KCl, 10 mM HEPES, pH 8.0 was used. A +160 mV potential was continuously applied. vitamin C was added to *trans* with a final concentration of 0-2 mM, marked on the left of each corresponding trace. The rate of event appearance increases when the vitamin C concentration is raised. **c.** Concentration dependence. The reciprocal of inter-event interval ($1/{\tau_{on}}$) and the reciprocal of dwell time ($1/{\tau_{off}}$) is plotted against the final concentration of vitamin C. $1/{\tau_{on}}$ demonstrates a linear correlation with the concentration of vitamin C. $1/{\tau_{off}}$ stays constant. Error bars=Standard Deviations (N=3). **d.** Scatter plot of $\Delta I$ vs. $t_{off}$. 120 events are included in the scatter plot. The histogram of $\Delta I$, superimposed with its Gaussian fitting result, is plotted to the right of the scatter plot. The vitamin C concentration was 1.6 mM. The events were extracted from a 15 min continuously recorded trace.


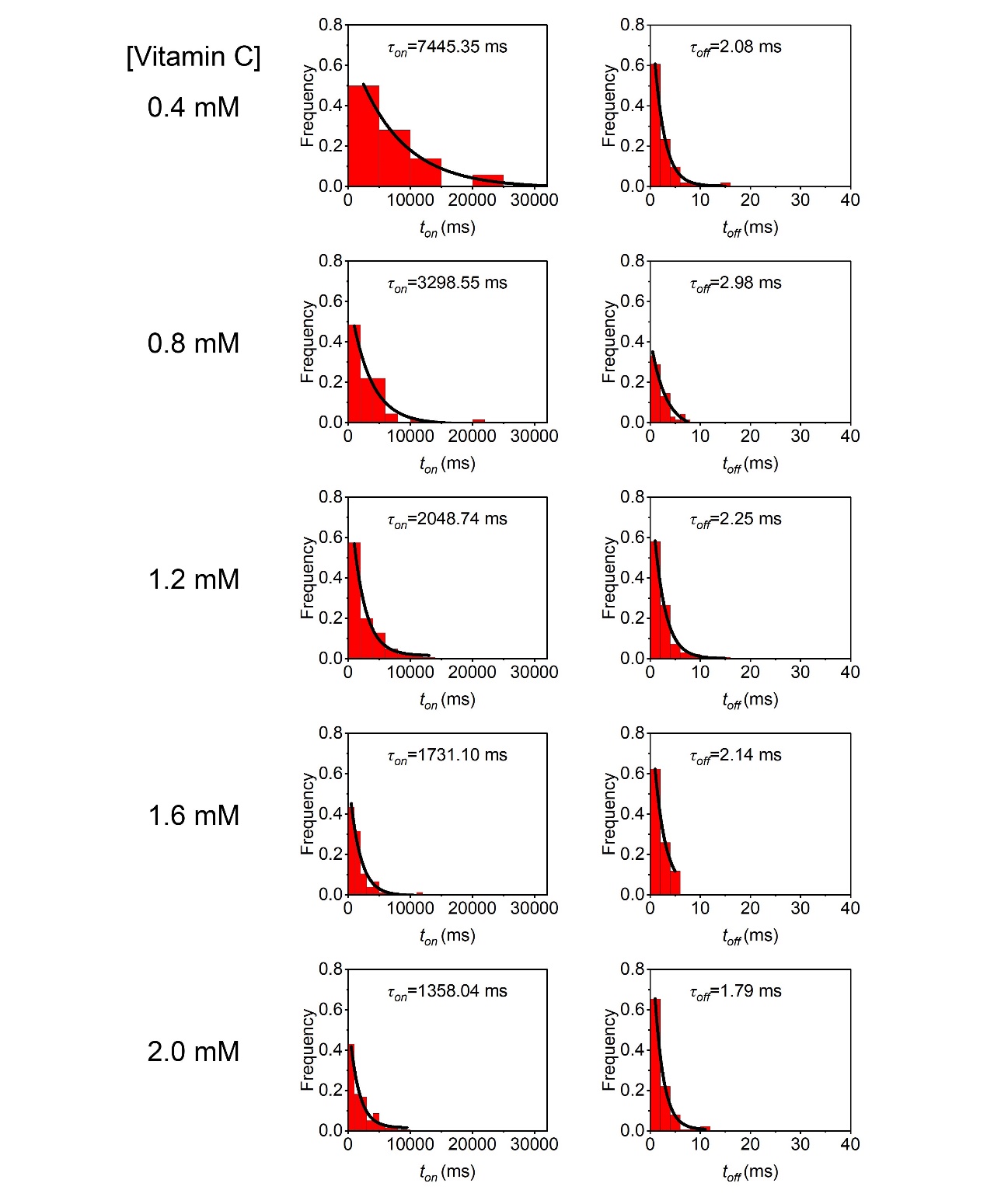


Supplementary fig. 30**|** $\boldsymbol{\tau}_{\boldsymbol{on}}$ **and** $\boldsymbol{\tau}_{\boldsymbol{off}}$ **of vitamin C binding to a PBA.** Histograms of the inter-event interval ($t_{on}$) and the event dwell time ($t_{off}$) with different vitamin C concentrations are presented. Vitamin C was added to *trans* with a final concentration of 0.4-2.0 mM. The applied concentration is marked on the left of each corresponding histogram. All histograms were respectively fit with a single exponential function $y=a*exp(-x/\tau)$, from which the mean inter-event interval ($\tau_{on}$) and the mean event dwell time ($\tau_{off}$) were derived and marked in each corresponding histogram plot. The PNRSS measurements were performed as described in **Methods**. The PNRSS strand 14PBA **(Supplementary Table 1)** was applied. A buffer of 1.5 M KCl, 10 mM HEPES, pH 8.0 was used. A +160 mV potential was continuously applied.

**
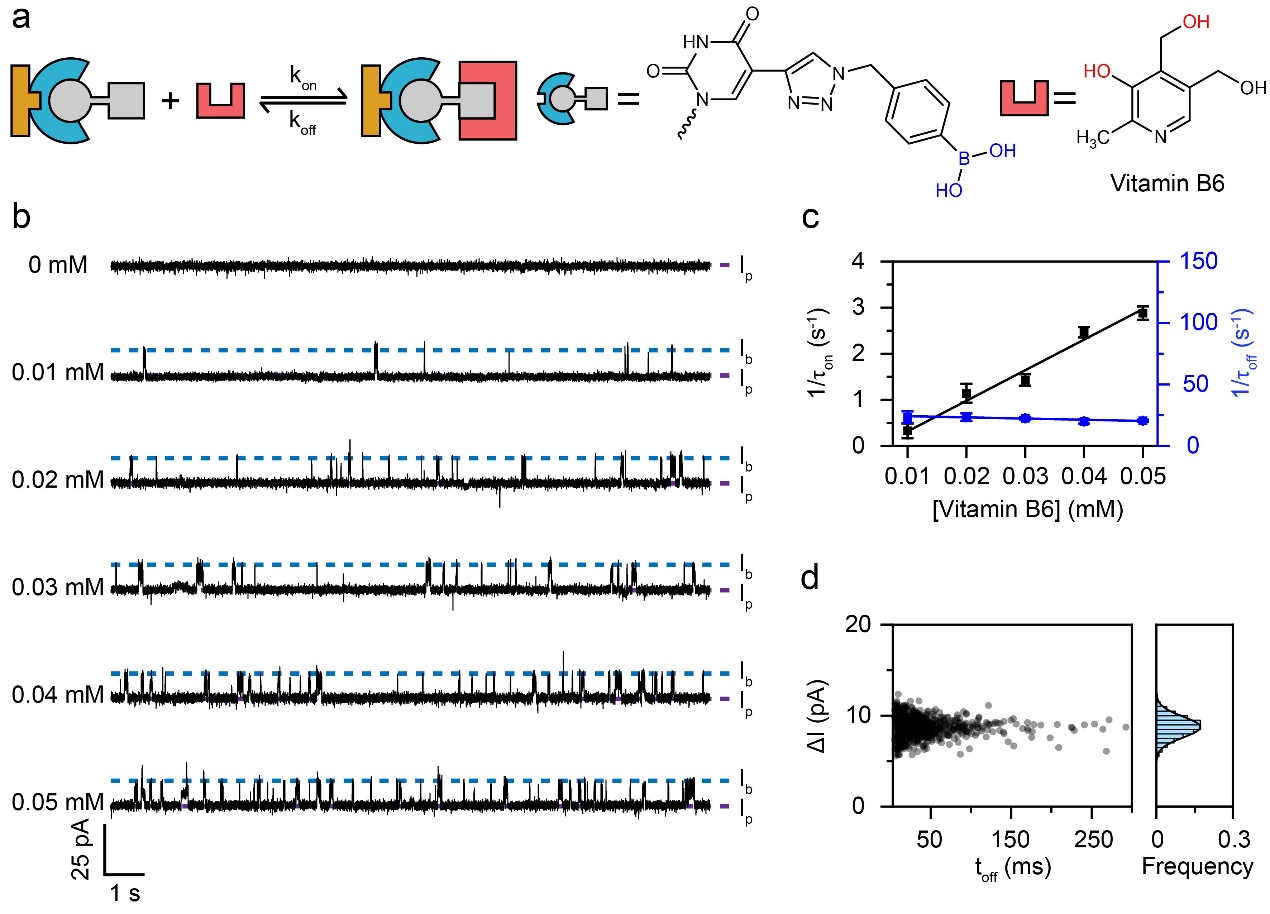
**

Supplementary fig. 31| Vitamin B6 binding to a PBA. **a.** The schematic diagram. The PNRSS strand 14PBA **(Supplementary Table 1)** contains a sole PBA at site 14, capable of binding vitamin B6[^11^](#_ENREF_11), as illustrated by the cartoon diagram. **b.** Representative traces containing vitamin B6 binding events. A buffer of 1.5 M KCl, 10 mM HEPES, pH 8.0 was used. A +160 mV potential was continuously applied. Vitamin B6 was added to *trans* with a final concentration of 0-0.05 mM, marked on the left of each corresponding trace. The rate of event appearance increases when the vitamin B6 concentration is raised. **c.** Concentration dependence. The reciprocal of inter-event interval ($1/{\tau_{on}}$) and the reciprocal of dwell time ($1/{\tau_{off}}$) is plotted against the final concentration of vitamin B6. ($1/{\tau_{on}}$) demonstrates a linear correlation with the concentration of vitamin B6**.** ($1/{\tau_{off}}$) stays constant. Error bars=Standard Deviations (N=3). **d.** Scatter plot of $\Delta I$ vs. $t_{off}$. 763 events are included in the scatter plot. The histogram of $\Delta I$, superimposed with its Gaussian fitting result, is plotted to the right of the scatter plot. The vitamin B6 concentration was 40 µM. The events were extracted from a 15 min continuously recorded trace.


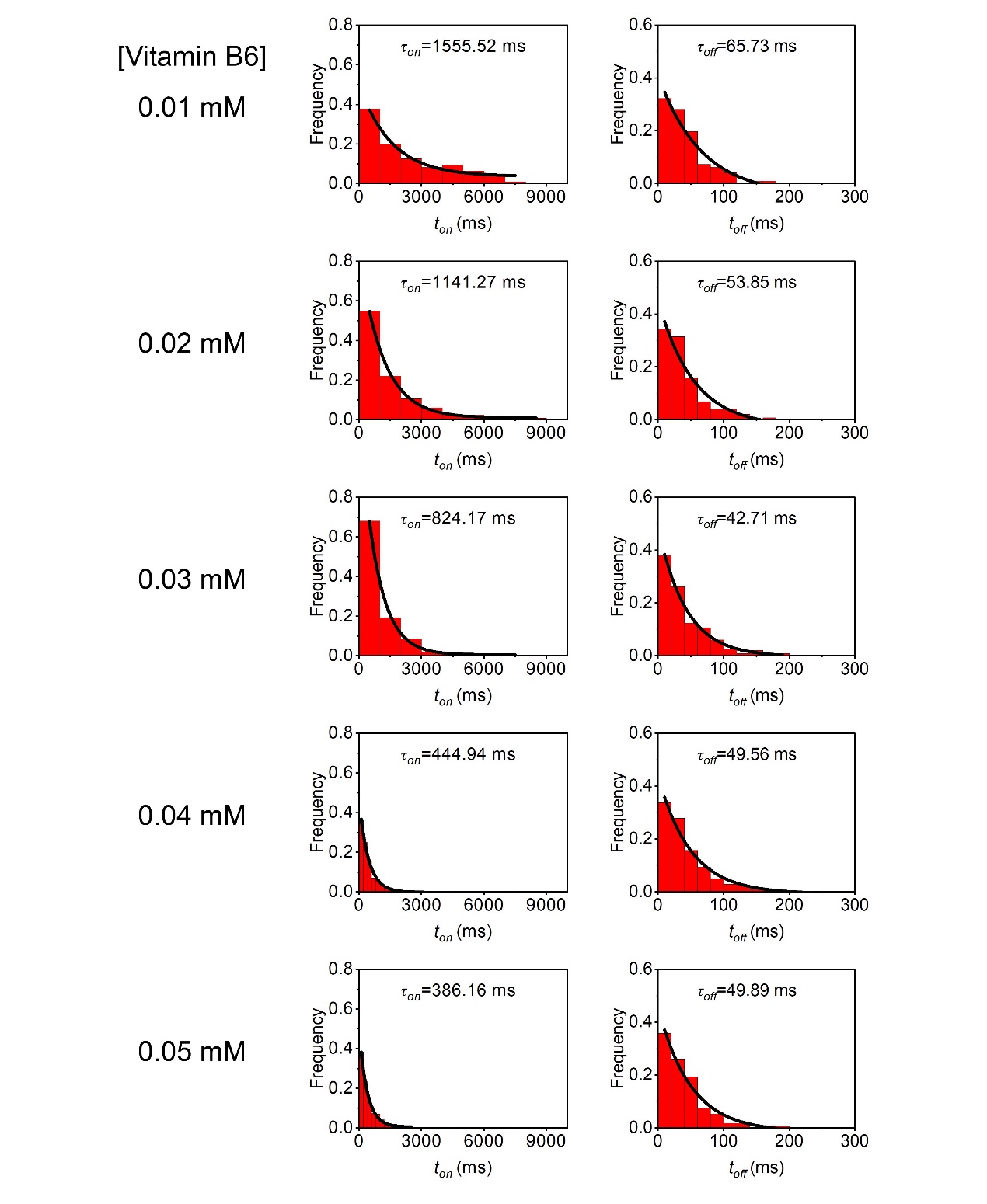


Supplementary fig. 32**|** $\boldsymbol{\tau}_{\boldsymbol{on}}$ **and** $\boldsymbol{\tau}_{\boldsymbol{off}}$ **of vitamin B6 binding to a PBA.** Histograms of the inter-event interval ($t_{on}$) and the event dwell time ($t_{off}$) with different vitamin B6 concentrations are presented. Vitamin B6 was added to *trans* with a final concentration of 0.01-0.05 mM. The applied concentration is marked on the left of corresponding histogram plot. All histograms were respectively fit with a single exponential function $y=a*exp(-x/\tau)$, from which the mean inter-event interval ($\tau_{on}$) and the mean event dwell time ($\tau_{off}$) were derived and marked on each corresponding histogram plot. The PNRSS measurements were performed as described in **Methods**. The PNRSS strand 14PBA **(Supplementary Table 1)** was applied. A buffer of 1.5 M KCl, 10 mM HEPES, pH 8.0 was used. A +160 mV potential was continuously applied.

**
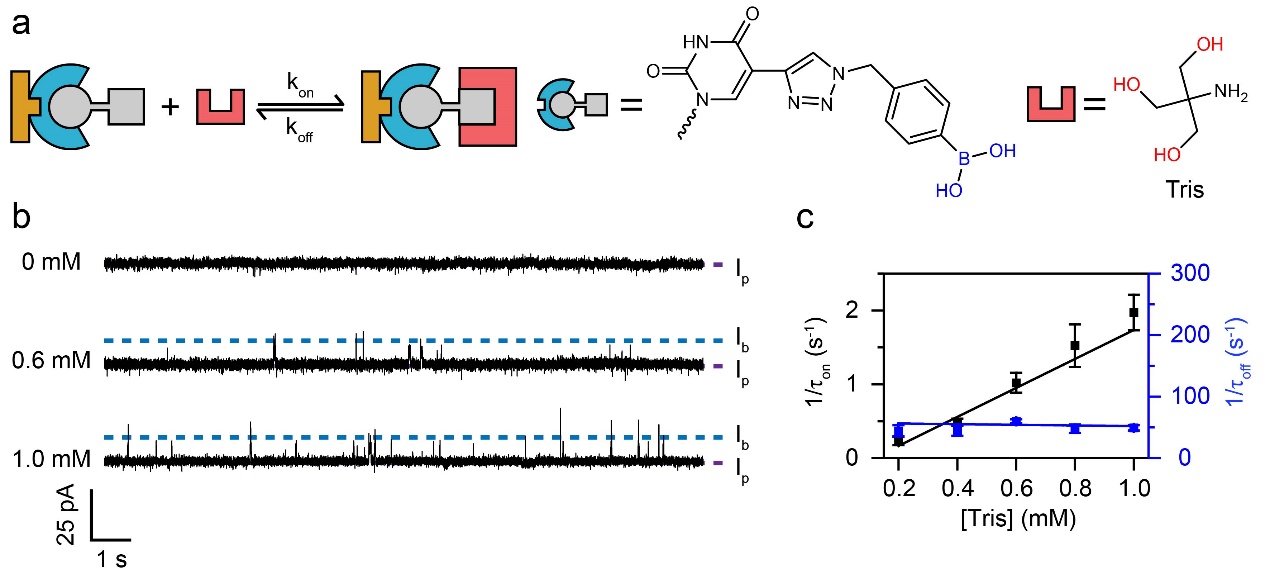
**

Supplementary fig. 33| Tris binding to a PBA. **a.** The schematic diagram. The PNRSS strand 14PBA **(Supplementary Table 1)** contains a sole PBA at site 14, capable of binding tris, as illustrated by the cartoon diagram. **b.** Representative traces containing tris binding events. A buffer of 1.5 M KCl, 10 mM HEPES, pH 8.0 was used. A +160 mV potential was continuously applied. Tris was added to *trans* with a final concentration of 0-1.0 mM, marked on the left of each corresponding trace. The rate of event appearance is increased when the tris concentration is raised. **c.** Concentration dependence. The reciprocal of inter-event interval ($1/{\tau_{on}}$) and the reciprocal of dwell time ($1/{\tau_{off}}$) is plotted against the final concentration of tris. ($1/{\tau_{on}}$) demonstrates a linear correlation with the concentration of tris**.** ($1/{\tau_{off}}$) remains constant. The events were extracted from a 15 min continuously recorded trace for each condition. Error bars=Standard Deviations (N=3).


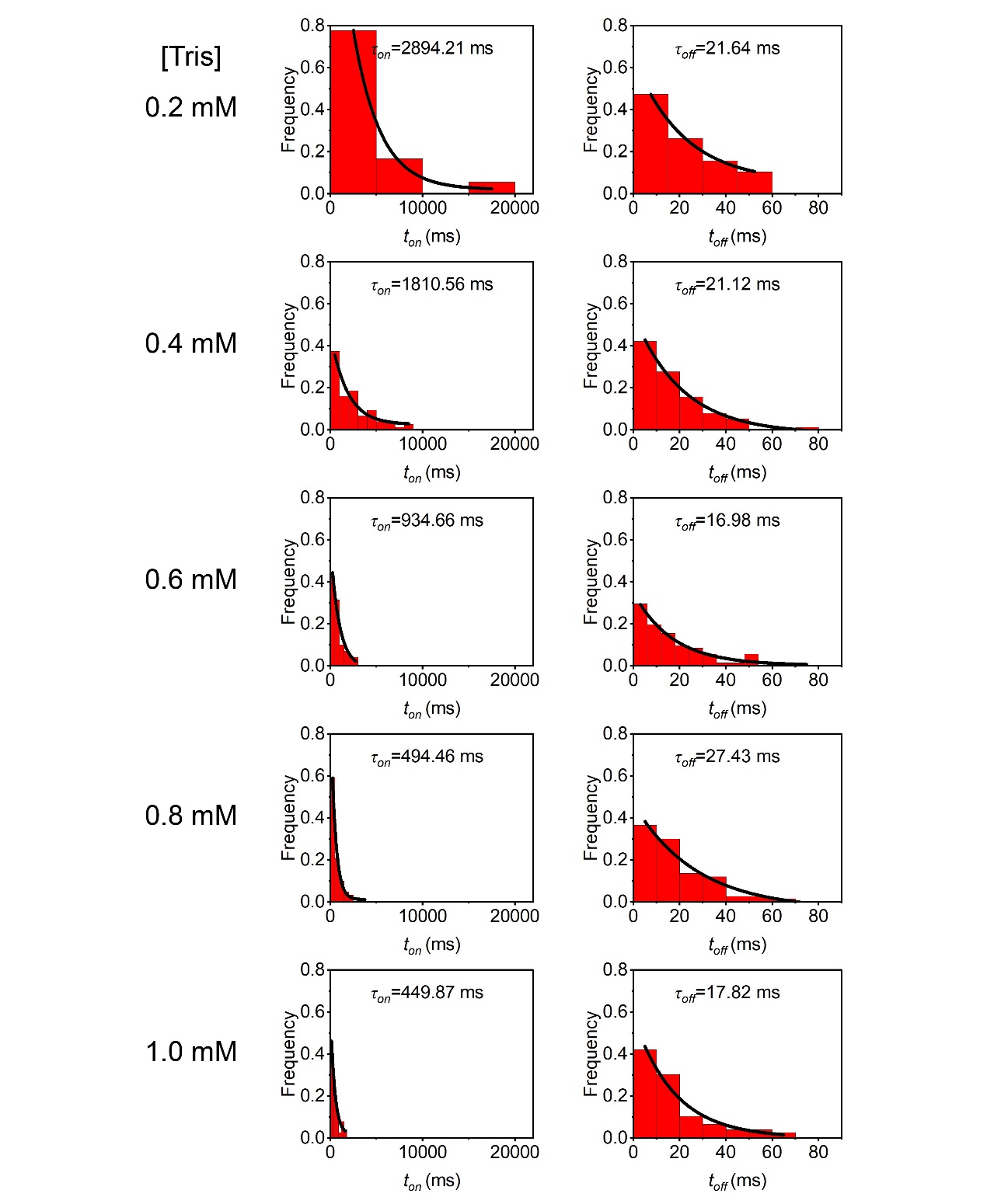


Supplementary fig. 34**|** $\boldsymbol{\tau}_{\boldsymbol{on}}$ **and** $\boldsymbol{\tau}_{\boldsymbol{off}}$ **of tris binding to a PBA.** Histograms of the inter-event interval ($t_{on}$) and the event dwell time ($t_{off}$) with different tris concentrations are presented. Tris was added to *trans* with a final concentration of 0.2-1.0 mM. The applied concentration was marked on the left of corresponding histogram plot. All histograms were respectively fit with a single exponential function $y=a*exp(-x/\tau)$, from which the mean inter-event interval ($\tau_{on}$) and the mean event dwell time ($\tau_{off}$) were derived and marked on each corresponding histogram plot. The PNRSS measurements were performed as described in **Methods**. The PNRSS strand 14PBA **(Supplementary Table 1)** was applied. A buffer of 1.5 M KCl, 10 mM HEPES, pH 8.0 was used. A +160 mV potential was continuously applied.

**
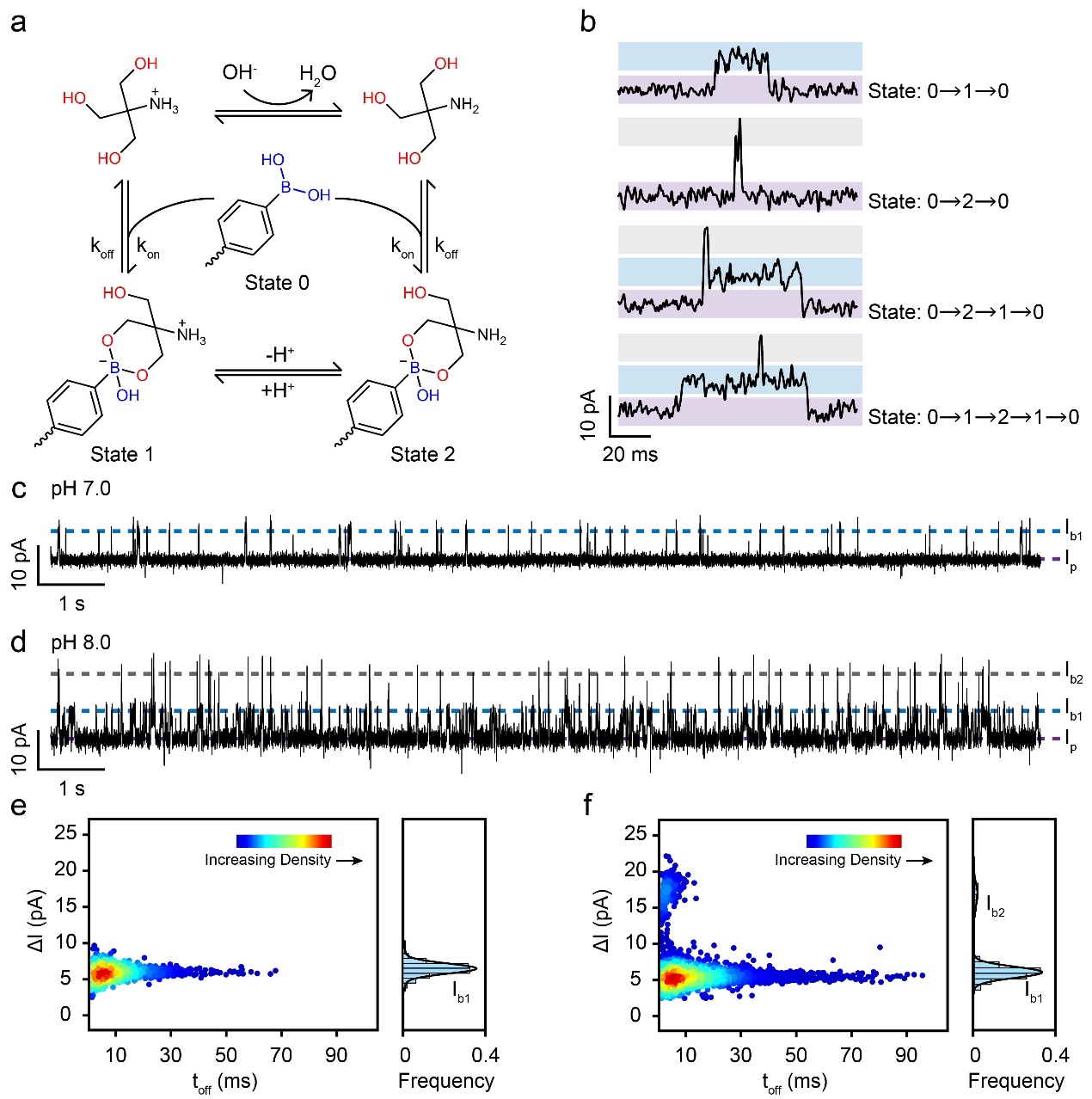
**

Supplementary fig. 35| Observing chemical intermediates with PNRSS. **a.** A suggested reaction model of tris, when bound with a PBA. Tris could be protonated or deprotonated depending on the environment pH. **b.** Representative PNRSS events containing chemical intermediates, acquired at pH 8.0. Transition between state 0, 1 or 2 were observed. Transition to other states was however never observed. **c-d.** Representative traces acquired at pH 7.0 **(c)** or at pH 8.0 **(d)**. At pH 7.0, tris binding to a PBA results in only one type of blockage level $I_{b1}$. However, at pH 8.0, tris binding results in a new blockage level $I_{b2}$, on top of $I_{b1}$. **e-f.** Event scatter plots of $\Delta I$ vs. $t_{off}$ formed from events acquired at pH 7.0 **(e)** or pH 8.0 **(f)**. In the scatter plot, a new event population of $I_{b2}$ in $\Delta I$ is observed at pH 8.0. All above measurements were carried out as described in **Methods**. 1577 events are included in **e**. 8261 events are included in **f**. Measurement at a higher pH results in a much higher rate of event appearance. 14PBA was applied as the PNRSS strand. All above measurements were performed with a buffer of 1.5 M KCl, 10 mM tris. A +160 mV potential was continuously applied. The scatter plots **(e, f)** were formed from continuous 15 min recordings for each condition. The colour scale in **e** and **f** represents the local density around each point. The density scatter plot was generated using the ggplot2 package of R.

**
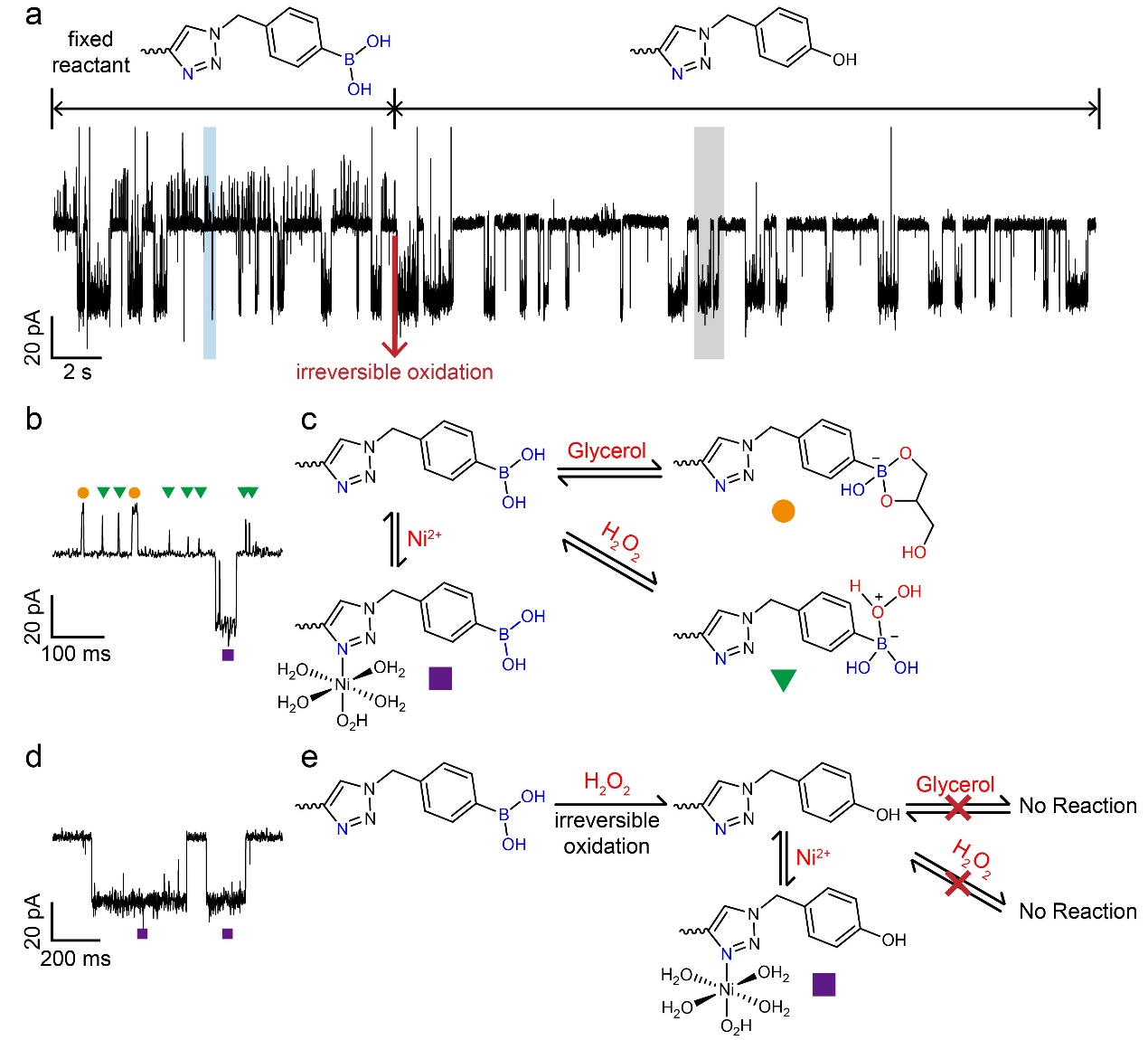
**

Supplementary fig. 36| Investigation of the chemical nature of irreversible oxidation of a PBA. The PNRSS measurement was carried out similarly to that described in **Fig. 4**. The electrolyte buffer was 1.5 M KCl, 10 mM HEPES, pH 8.0. The PNRSS strand 14 PBA was added to *cis* with a 10 nM final concentration. H_2_O_2_, Ni^2+^ and glycerol were simultaneously added to *trans* with a 5.4 mM, a 0.2 mM and a 8 mM final concentration respectively. A +160 mV potential was continuously applied. **a.** A representative trace acquired during PNRSS. Binding of H_2_O_2_ or glycerol to a PBA results in positive going events, whereas binding of Ni^2+^ results in negative going events. The PBA may as well be irreversibly oxidized by H_2_O_2_ to generate a phenol (red arrow marked). Afterwards, binding of H_2_O_2_ or glycerol are no longer observed from the trace. **b.** A zoomed-in view of a trace segment from **a** (blue marked). Binding of H_2_O_2_ (green triangle), glycerol (orange circle) and Ni^2+^ (Purple square) are labelled respectively on the trace. **c.** The proposed mechanism of the binding. **d.** A zoomed-in view of a trace segment from **a** (grey marked). Only Ni^2+^ bindings (purple square) are still observable. **e.** The proposed mechanism. The disappearance of H_2_O_2_ and glycerol binding event and the retaining of Ni^2+^ binding have confirmed the hypothesis that the PBA has been irreversibly oxidized to a phenol.

**
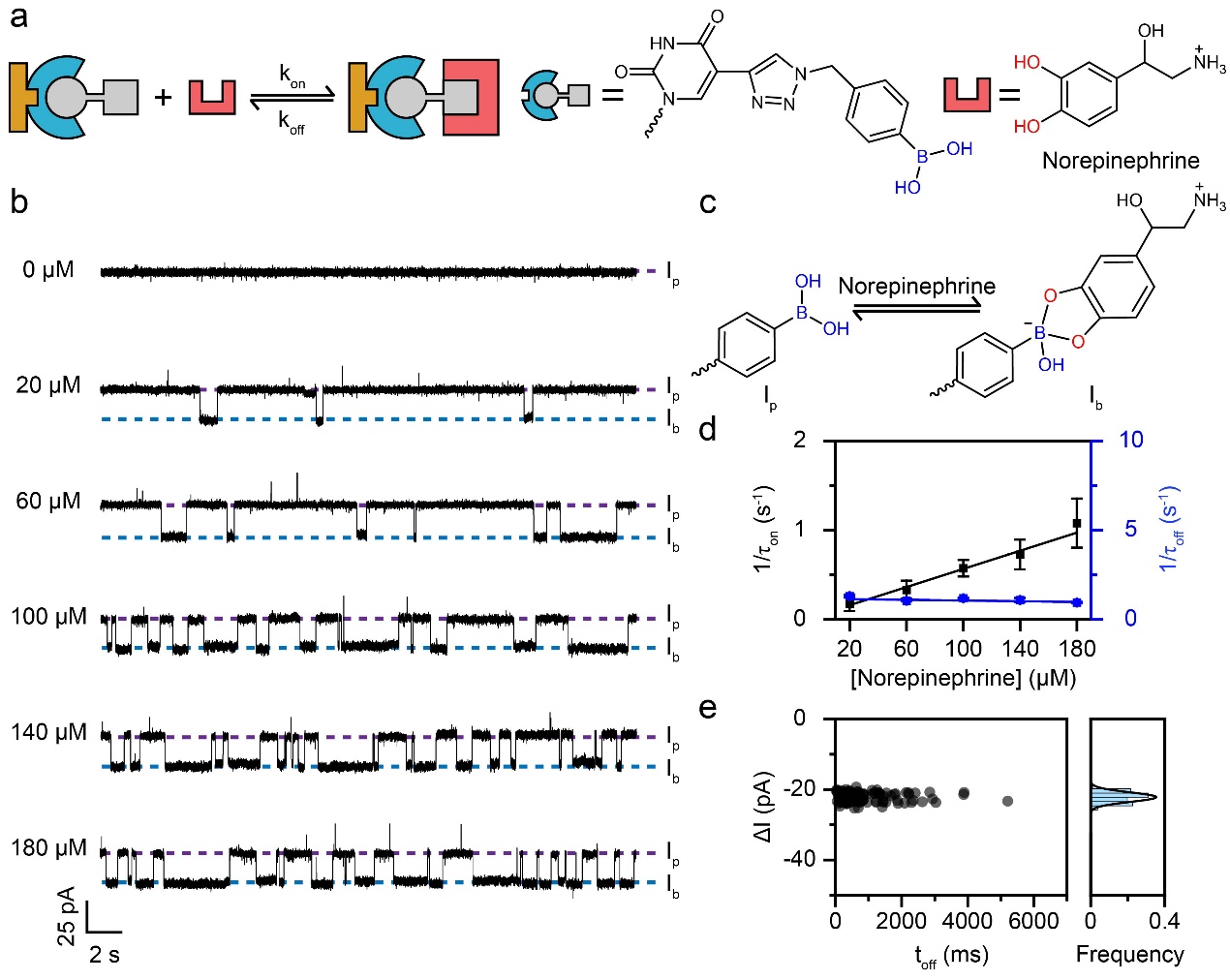
**

Supplementary fig. 37| Norepinephrine binding to a PBA. **a.** The schematic diagram. The PNRSS strand 14PBA **(Supplementary Table 1)** contains a sole PBA at site 14, capable of binding norepinephrine, as illustrated by the cartoon diagram. **b.** Representative traces containing norepinephrine binding events. PNRSS measurements were carried out as described in **Fig. 5**. The electrolyte buffer was 1.5 M KCl, 10 mM HEPES, pH 8.0. A +160 mV potential was continuously applied. Norepinephrine was added to *trans* with a final concentration of 0-180 µM, marked on the left of each corresponding trace. The rate of event appearance increases when the norepinephrine concentration is raised. **c.** The reactive mechanism [^12^](#_ENREF_12). **d.** Concentration dependence. The norepinephrine concentration was modulated between 20-180 µM. 15 min continuous recording was performed for each condition. $\tau_{on}$ and $\tau_{off}$ values were derived as described in **Supplementary fig. 4**. The reciprocal of inter-event interval ($1/{\tau_{on}}$) and the reciprocal of dwell time ($1/{\tau_{off}}$) is plotted against the final concentration of norepinephrine. ($1/{\tau_{on}}$) demonstrates a linear correlation with the concentration of norepinephrine. ($1/{\tau_{off}}$) stays constant. Error bars=Standard Deviations (N=3). **e.** Scatter plot of $\Delta I$ vs. $t_{off}$. 106 events are included in the scatter plot. The histogram of $\Delta I$, superimposed with its Gaussian fitting result, is plotted to the right of the scatter plot. The norepinephrine concentration was 140 µM. The events were extracted from a 15 min continuously recorded trace.


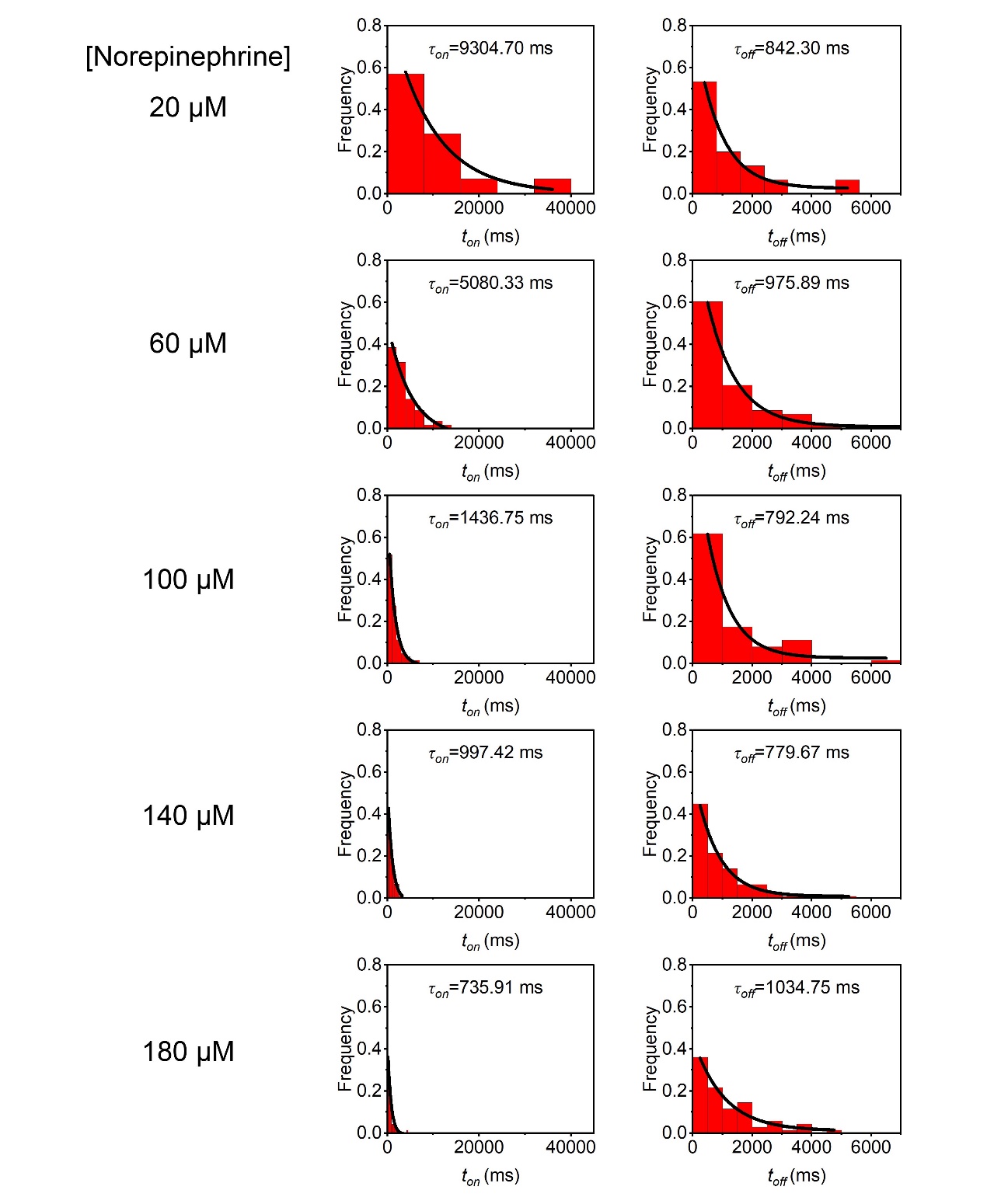


Supplementary fig. 38**|** $\boldsymbol{\tau}_{\boldsymbol{on}}$ **and** $\boldsymbol{\tau}_{\boldsymbol{off}}$ **of norepinephrine binding to a PBA.** Histograms of the inter-event interval ($t_{on}$) and the event dwell time ($t_{off}$) with different norepinephrine concentrations are presented. Norepinephrine was added to *trans* with a final concentration of 20-180 µM. The applied concentration is marked on the left of each corresponding histogram plot. All histograms were respectively fit with a single exponential function $y=a*exp(-x/\tau)$, from which the mean inter-event interval ($\tau_{on}$) and the mean event dwell time ($\tau_{off}$) were derived and marked on each corresponding histogram plot. The PNRSS measurements were performed as described in **Methods**. The PNRSS strand 14PBA **(Supplementary Table 1)** was applied. A buffer of 1.5 M KCl, 10 mM HEPES, pH 8.0 was used. A +160 mV potential was continuously applied.

**
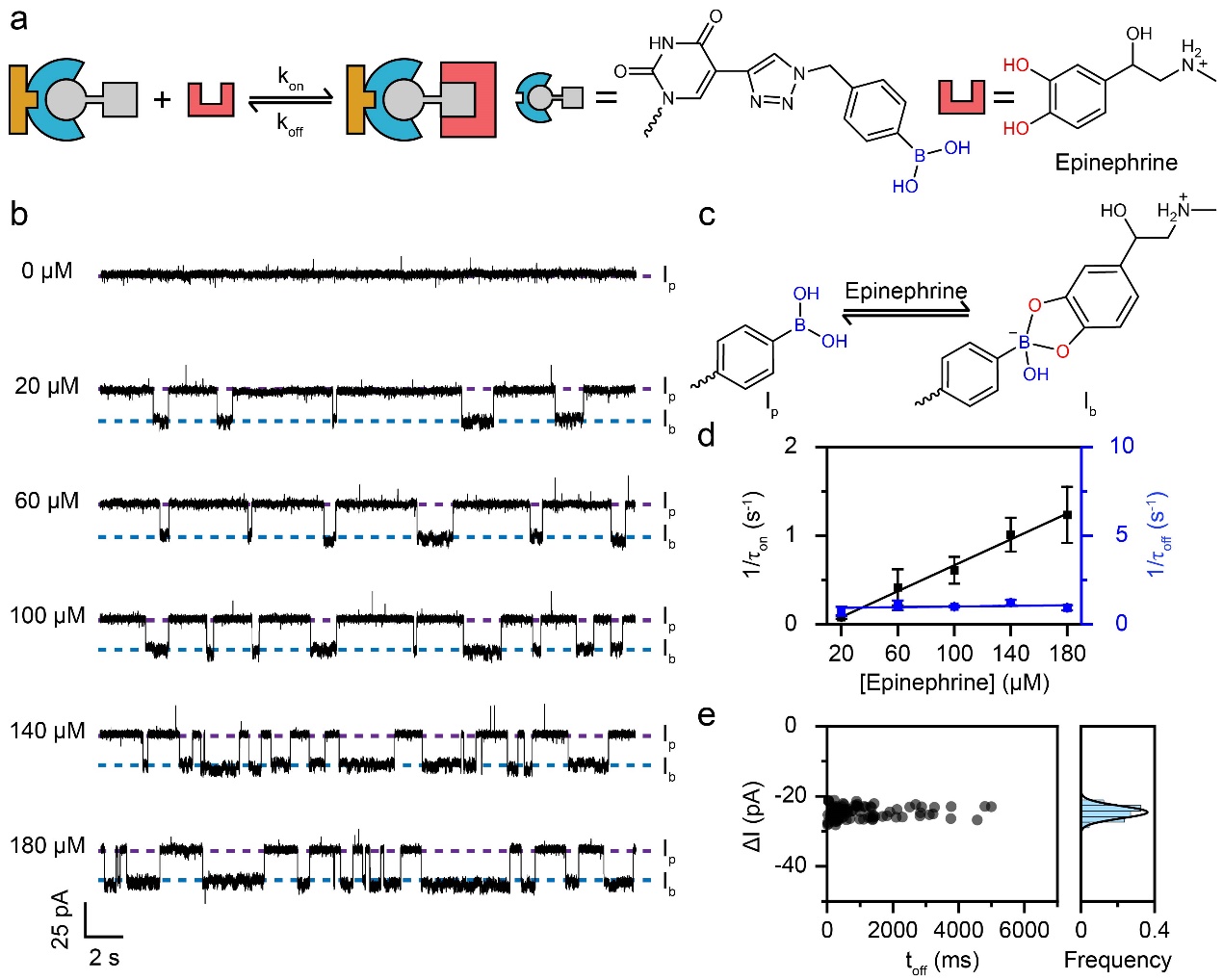
**

Supplementary fig. 39| Epinephrine binding to a PBA. **a.** The schematic diagram. The PNRSS strand 14PBA **(Supplementary Table 1)** contains a sole PBA at site 14, capable of binding epinephrine, as illustrated by the cartoon diagram. **b.** Representative traces containing epinephrine binding events. PNRSS measurements were carried out as described in **Fig. 5**. The electrolyte buffer was 1.5 M KCl, 10 mM HEPES, pH 8.0. A +160 mV potential was continuously applied. Epinephrine was added to *trans* with a final concentration of 0-180 µM, marked on the left of each corresponding trace. The rate of event appearance increases when the epinephrine concentration is raised. **c.** The reactive mechanism[^12^](#_ENREF_12). **d.** Concentration dependence. The epinephrine concentration was modulated between 20-180 µM. 15 min continuously recording was performed for each condition. $\tau_{on}$ and $\tau_{off}$ values were derived as described in **Supplementary fig. 4**. The reciprocal of inter-event interval ($1/{\tau_{on}}$) and the reciprocal of dwell time ($1/{\tau_{off}}$) are plotted against the final concentration of epinephrine. ($1/{\tau_{on}}$) demonstrates a linear correlation with the concentration of epinephrine. ($1/{\tau_{off}}$) stays constant. Error bars=Standard Deviations (N=3). **e.** Scatter plot of $\Delta I$ vs. $t_{off}$. The scatter plot was generated from a 15 min continuously recorded trace. 109 events are included in the scatter plot. The histogram of $\Delta I$, superimposed with its Gaussian fitting result, is plotted to the right of the scatter plot. The epinephrine concentration was 140 µM. The events were extracted from a 15 min continuously recorded trace.


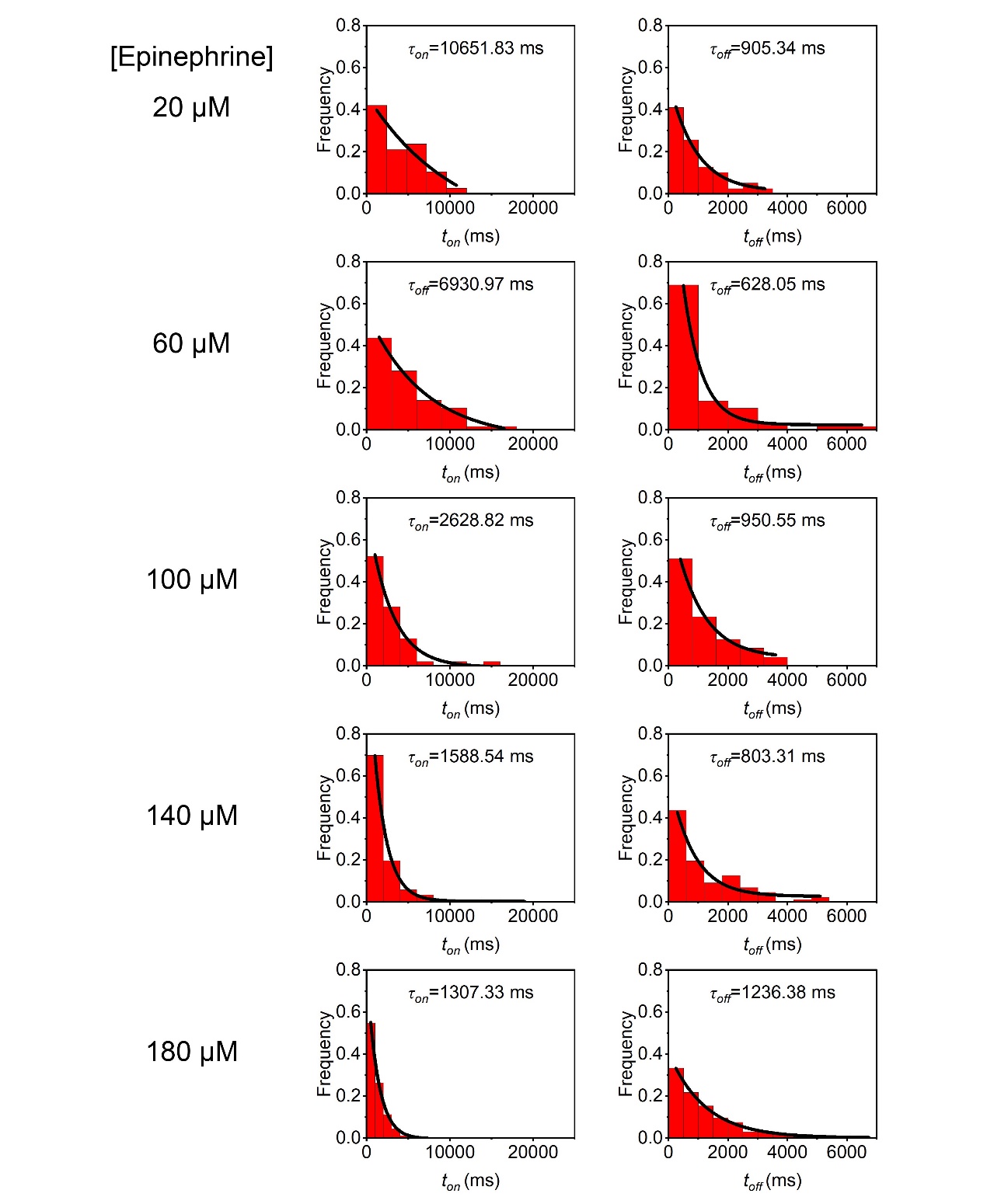


Supplementary fig. 40**|** $\boldsymbol{\tau}_{\boldsymbol{on}}$ **and** $\boldsymbol{\tau}_{\boldsymbol{off}}$ **of epinephrine binding to a PBA.** Histograms of the inter-event interval ($t_{on}$) and the event dwell time ($t_{off}$) with different epinephrine concentrations are presented. Epinephrine was added to *trans* with a final concentration of 20-180 µM. The applied concentration was marked on the left of each corresponding histogram plot. All histograms were respectively fit with a single exponential function $y=a*exp(-x/\tau)$, from which the mean inter-event interval ($\tau_{on}$) and the mean event dwell time ($\tau_{off}$) were derived and marked on each corresponding histogram plot. The PNRSS measurements were performed as described in **Methods**. The PNRSS strand 14PBA **(Supplementary Table 1)** was applied. A buffer of 1.5 M KCl, 10 mM HEPES, pH 8.0 was used. A +160 mV potential was continuously applied.

**
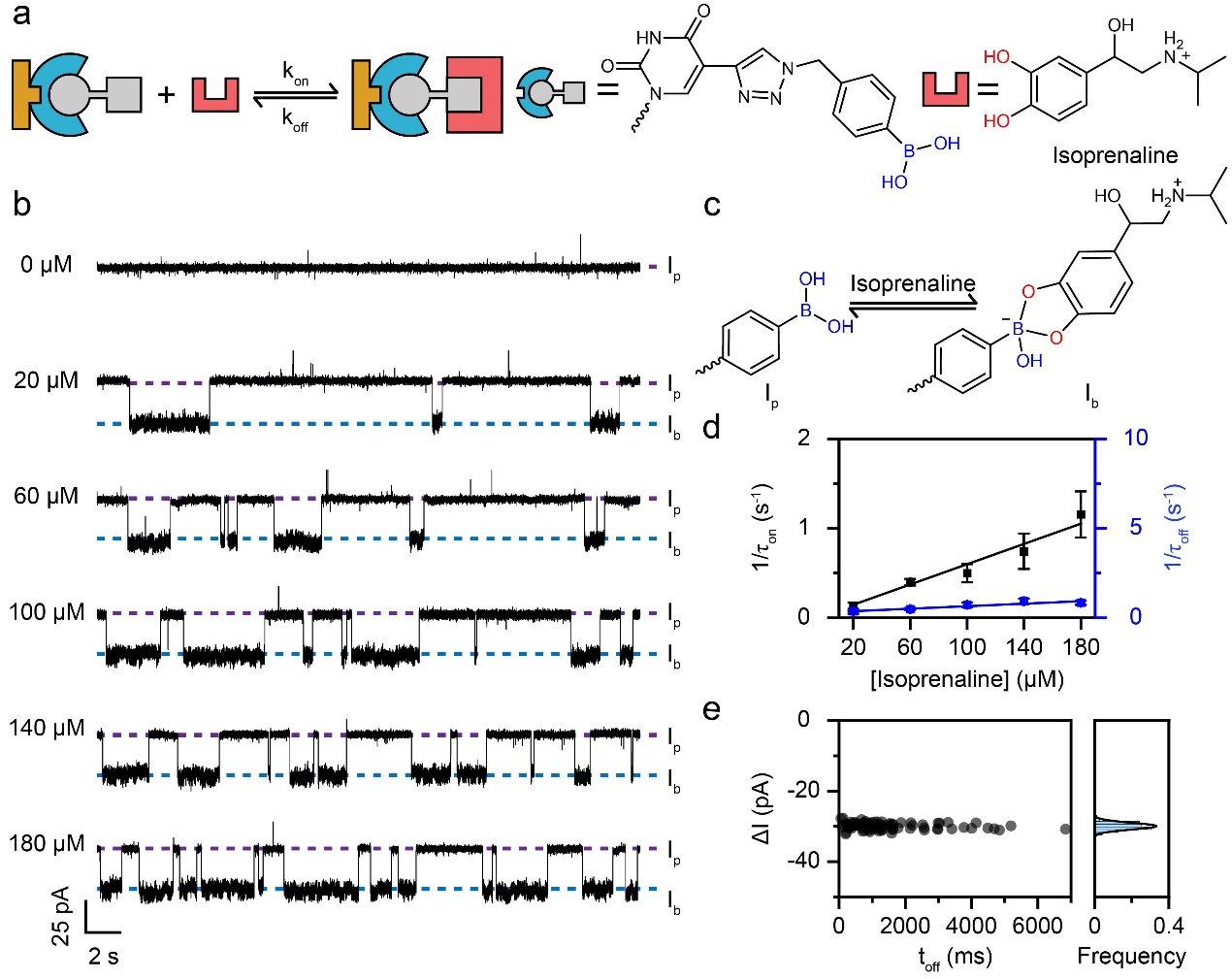
**

Supplementary fig. 41| Isoprenaline binding to a PBA. **a.** The schematic diagram. The PNRSS strand 14PBA **(Supplementary Table 1)** contains a sole PBA at site 14, capable of binding isoprenaline, as illustrated by the cartoon diagram. **b.** Representative traces containing isoprenaline binding events. PNRSS measurements were carried out as described in **Fig. 5**. The electrolyte buffer was 1.5 M KCl, 10 mM HEPES, pH 8.0. A +160 mV potential was continuously applied. Isoprenaline was added to *trans* with a final concentration of 0-180 µM, marked on the left of each corresponding trace. The rate of event appearance increases when the isoprenaline concentration is raised. **c.** The reactive mechanism [^12^](#_ENREF_12). **d.** Concentration dependence. The isoprenaline concentration was modulated between 20-180 µM. 15 min continuous recording was performed for each condition. $\tau_{on}$ and $\tau_{off}$ values were derived as described in **Supplementary fig. 4**. The reciprocal of inter-event interval ($1/{\tau_{on}}$) and the reciprocal of dwell time ($1/{\tau_{off}}$) are plotted against the final concentration of isoprenaline. ($1/{\tau_{on}}$) demonstrates a linear correlation with the concentration of isoprenaline. ($1/{\tau_{off}}$) stays constant. Error bars=Standard Deviations (N=3). **e.** Event scatter plot of $\Delta I$ vs. $t_{off}$. The scatter plot was generated from a 15 min continuously recorded trace. 101 events are included in the scatter plot. The histogram of $\Delta I$, superimposed with its Gaussian fitting result, is plotted to the right of the scatter plot. The isoprenaline concentration was 140 µM. The events were extracted from a 15 min continuously recorded trace.


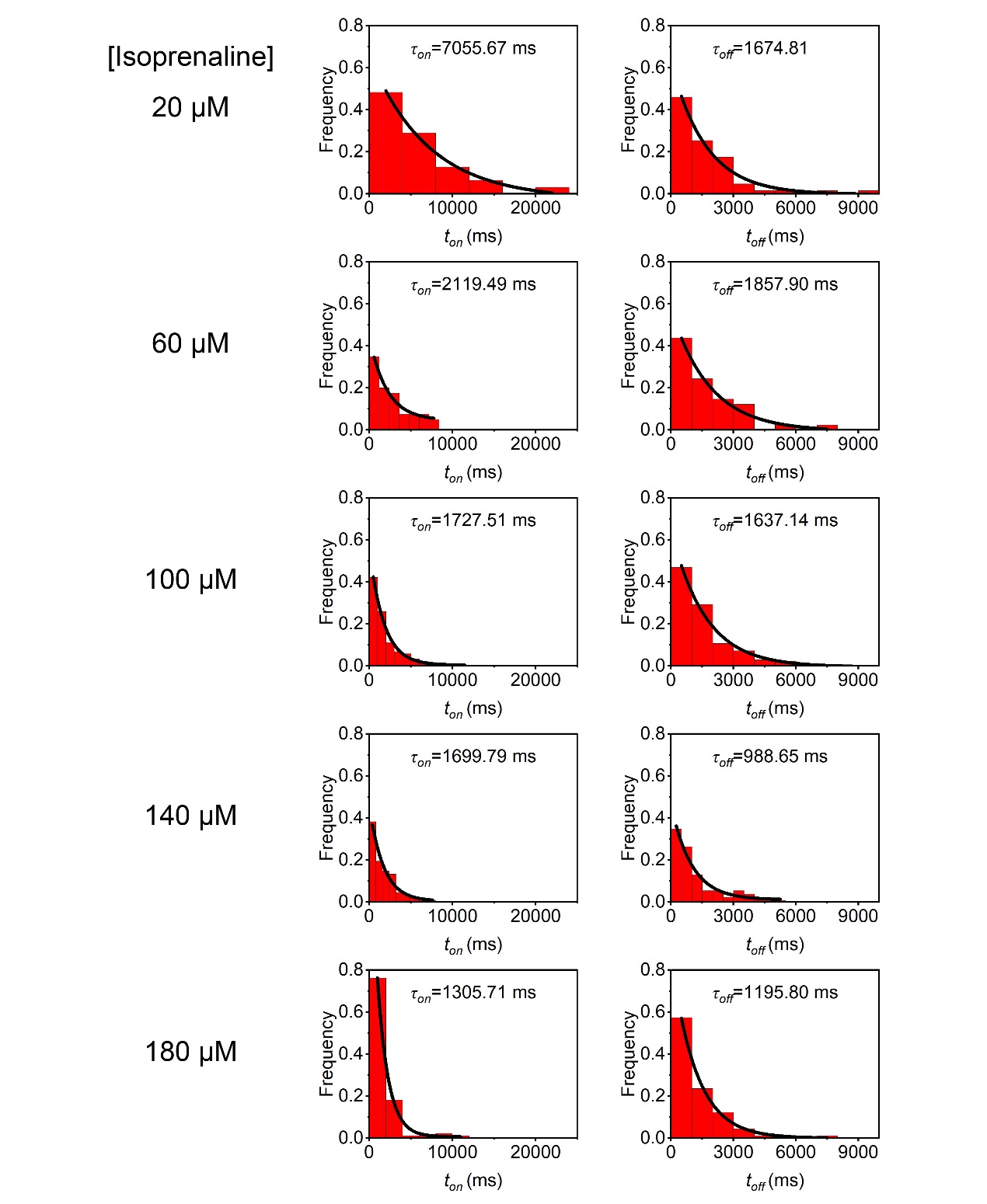


Supplementary fig. 42**|** $\boldsymbol{\tau}_{\boldsymbol{on}}$ **and** $\boldsymbol{\tau}_{\boldsymbol{off}}$ **of isoprenaline binding to a PBA.** Histograms of the inter-event interval ($t_{on}$) and the event dwell time ($t_{off}$) with different isoprenaline concentrations are presented. Isoprenaline was added to *trans* with a final concentration of 20-180 µM. The applied concentration is marked on the left of each corresponding histogram plot. All histograms were respectively fit with a single exponential function $y=a*exp(-x/\tau)$, from which the mean inter-event interval ($\tau_{on}$) and the mean event dwell time ($\tau_{off}$) were derived and marked on each corresponding histogram plot. The PNRSS measurements were performed as described in **Methods**. The PNRSS strand 14PBA **(Supplementary Table 1)** was applied. A buffer of 1.5 M KCl, 10 mM HEPES, pH 8.0 was used. A +160 mV potential was continuously applied.

**
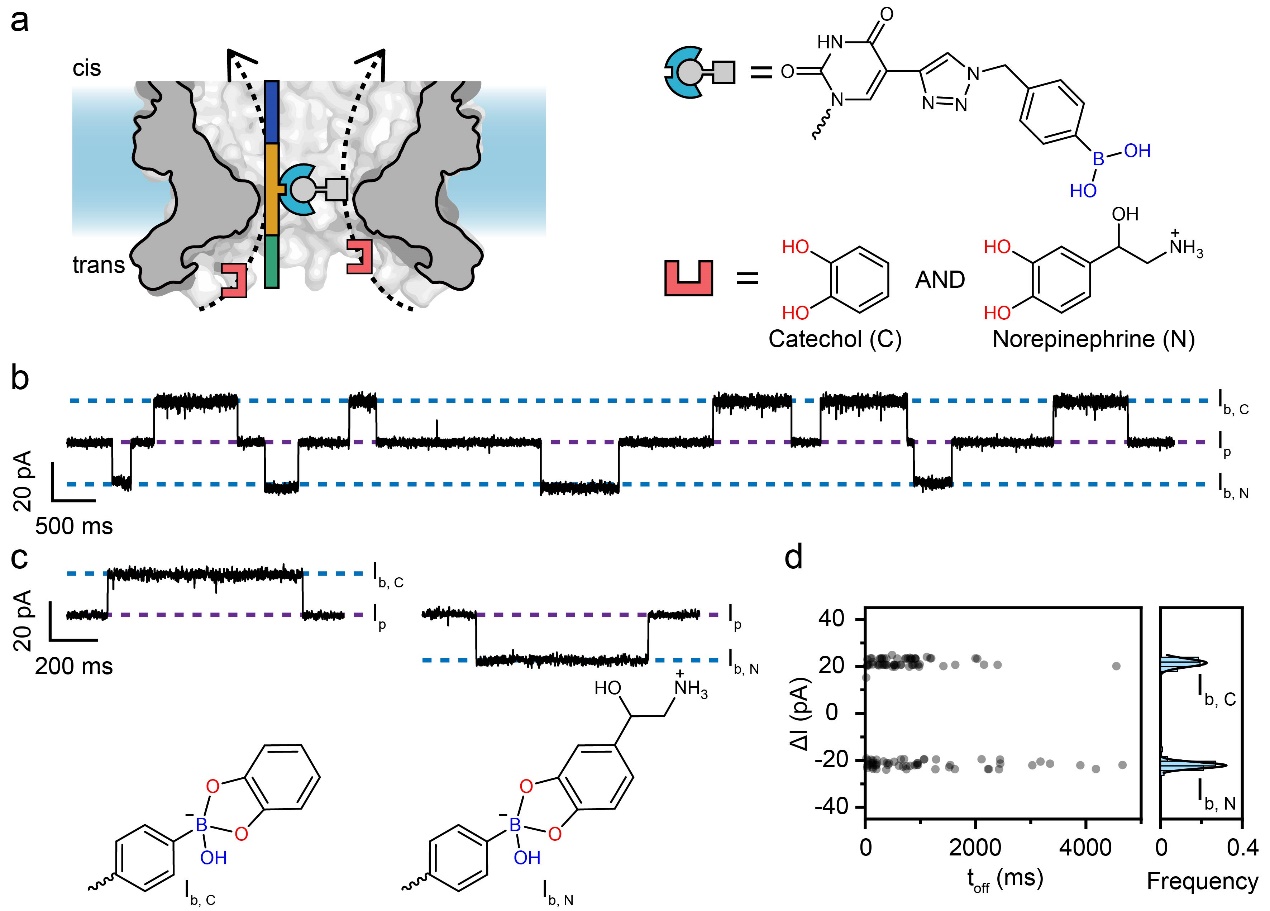
**

Supplementary fig. 43| Positive and negative going events. **a.** The schematic diagram. The PNRSS strand 14PBA **(Supplementary Table 1)** contains a sole PBA at site 14, capable of binding catechol or norepinephrine, as illustrated by the cartoon diagram. **b.** A representative trace containing catechol and norepinephrine binding events. Catechol and norepinephrine were simultaneously added to *trans*, respectively reaching a 400 µM and a 140 µM final concentration respectively. Binding of a catechol or a norepinephrine to a PBA respectively reports positive (${I_{b,C}, I}_{b,C}>I_{p}$) or negative going events ($I_{b,N}, I_{b,N}<I_{p}$). A visualized demonstration is also provided **(Supplementary Movie 4)**. **c.** A zoomed-in demonstration of different binding events. Representative events of binding from a catechol (top left) or a norepinephrine (top right) are demonstrated. The chemical structures of a catechol (bottom left) or a norepinephrine (bottom right) when bound to a PBA are also demonstrated. **d.** Scatter plot of $\Delta I$ vs. $t_{off}$. The events were extracted from a 15 min continuously recorded trace. A total of 119 events are included in the scatter plot. From the scatter plot, binding events from catechol and norepinephrine result in two clearly separated populations. The histogram of $\Delta I$ is plotted to the right of the scatter plot. Two peaks of $\Delta I$ were respective Gaussian fitted and superimposed on the histogram.

**
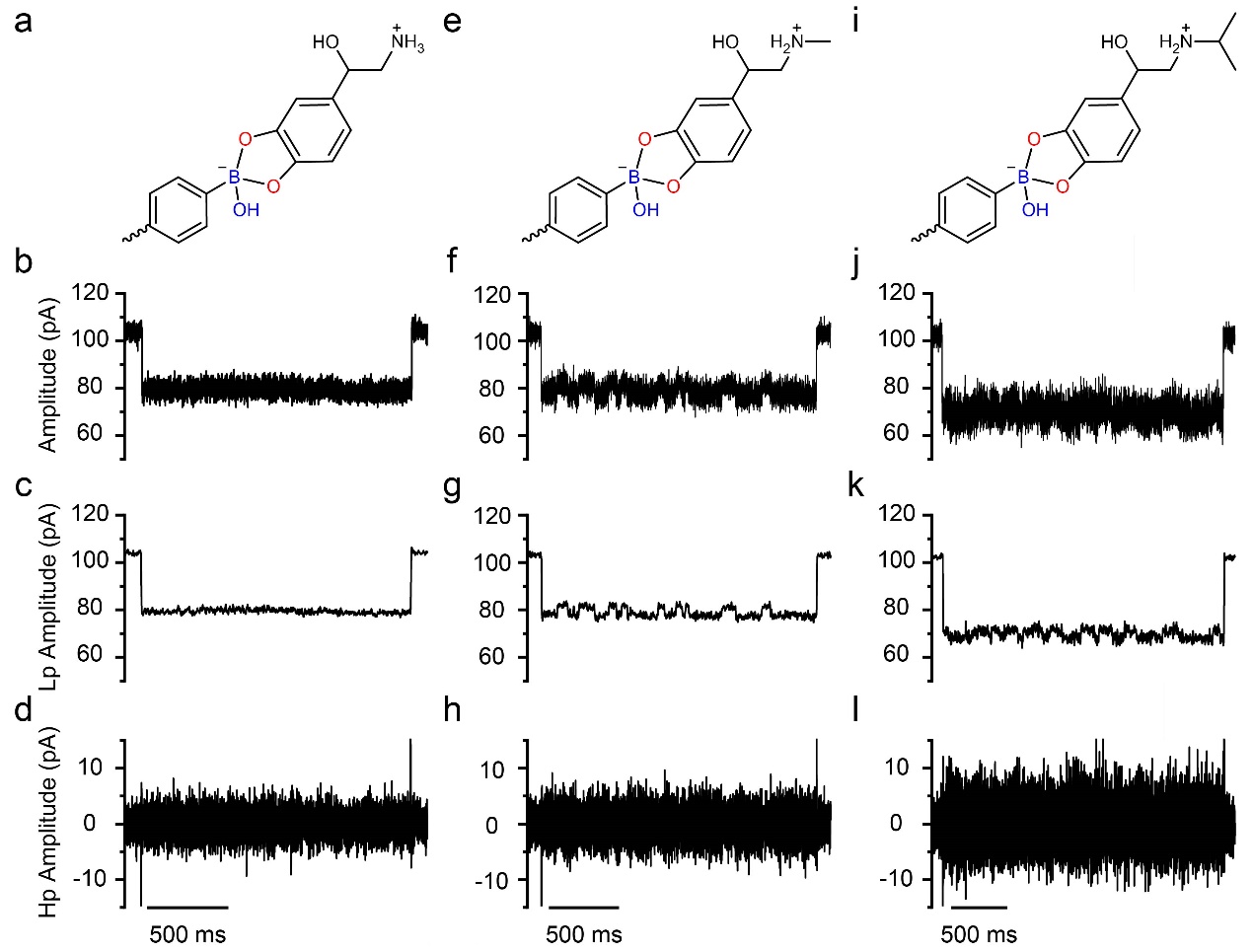
**

Supplementary fig. 44| Frequency split demonstration. When probed by PNRSS, binding of catecholamines, such as norepinephrine, epinephrine and isoprenaline, to a PBA results in rich information of chemical process, appearing as fluctuations in different frequency domains. The chemical structure of boronate ester complexes resulted from **(a)** norepinephrine, **(e)** epinephrine or **(i)** isoprenaline binding to a PBA are illustrated. During PNRSS, the raw trace was acquired with a 25 kHz sampling rate and low pass filtered at 1 kHz. The recorded traces were frequency split into low pass and the high pass portions, performed by Butterworth filtering. A cut-off frequency of 100 Hz and a filter order of 2 were selected. The raw events resulted from **(b)** norepinephrine, **(f)** epinephrine or **(j)** isoprenaline binding to a PBA were demonstrated. The low pass portion of the event resulted from **(c)** norepinephrine, **(g)** epinephrine or **(k)** isoprenaline binding to a PBA were demonstrated. The high pass portion of the event resulted from **(d)** norepinephrine, **(h)** epinephrine or **(l)** isoprenaline binding to a PBA were demonstrated. Specifically, norepinephrine shows no fluctuation in the low pass portion of the event, but epinephrine and isoprenaline demonstrate minor telegraphic switching. Isoprenaline can be distinguished from epinephrine by recognizing its unique noise characteristics in the high pass portion of the event.

**
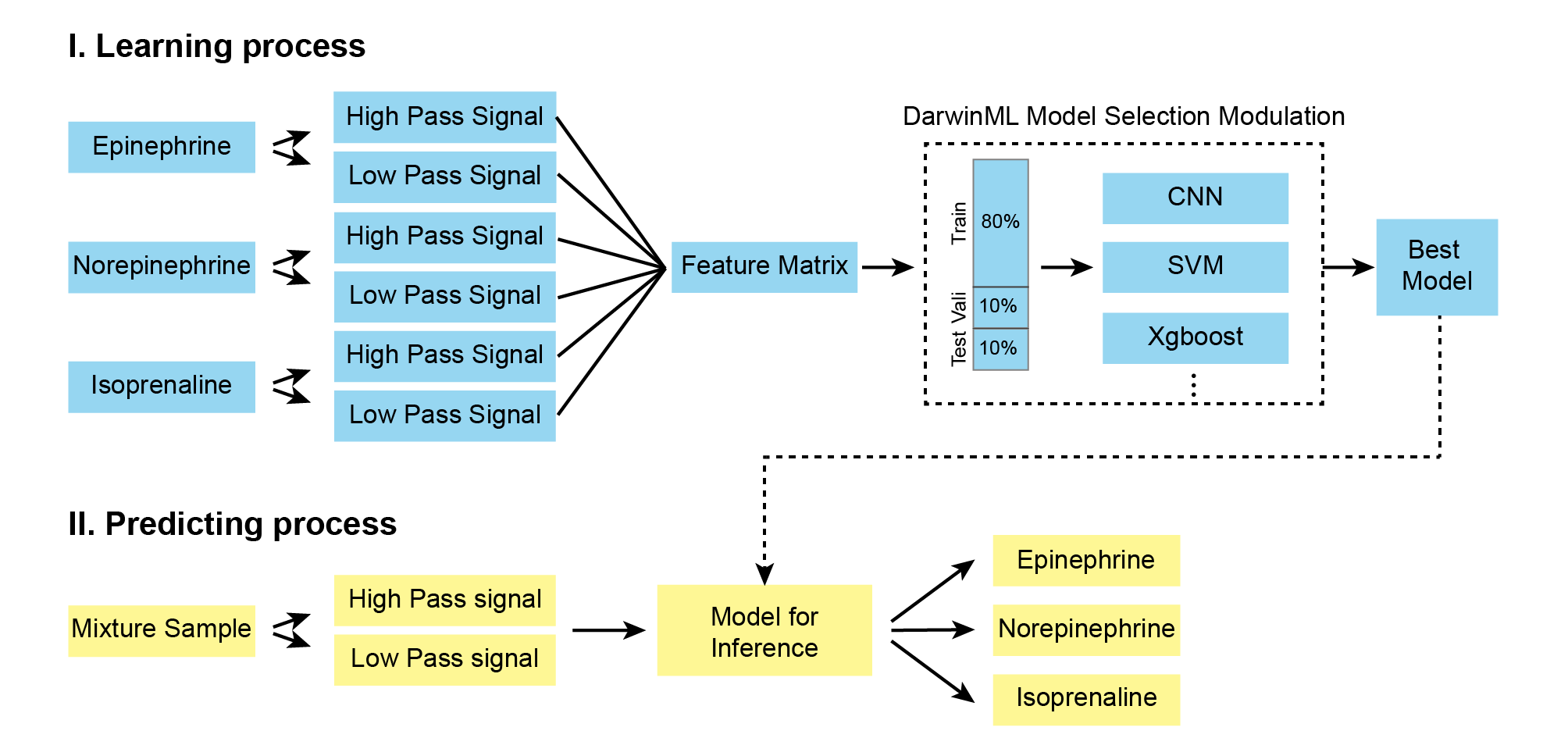
**

Supplementary fig. 45| The machine learning workflow. Machine learning was carried out with DarwinML®, a commercial AutoML platform developed based on an evolutionary algorithm for model automation design. To perform the learning process **(I)**, PNRSS measurements were respectively performed with norepinephrine, epinephrine or isoprenaline as the sole analyte **(Supplementary figs. 37-42)**. Raw time traces in abf files were extracted by the neo module (v0.8.0, https://pypi.org/project/neo-python/) in Python. Events in the traces were extracted by a custom event segmentation program, written by Python. The extracted events were then frequency split into the high pass and the low pass portion by a Butterworth filter, integrated in the SciPy module of Python. The cut off frequency was set to 100 Hz and the filter order was set to 2. Standard deviation of the high pass and the low pass portion were respectively calculated and applied to form a feature matrix. 1455 events in the feature matrix was fed into the DarwinML [^13^](#_ENREF_13) platform for model building. Briefly, 80%, 10% and 10% of the events were respectively used as the training, validation and test data sets. The training and the validation sets were used to build and validate the model. A 10-fold cross validation method was applied. More than 10 popular models, such as SVC (SVM for classification), logistic regression, Random Forest, XGboost, LightGBM, RidgeClassifier, MLPClassifier, BaggingClassifier and some others were applied. When evaluated with the test set (the remaining 10% of all 1455 events), the SVC model reported the highest accuracy score of 99.6%. The trained SVC model was further validated with all 1455 events and an overall accuracy score of 98.3% was reported. The confusion matrix result is demonstrated in **Fig. 5f**. To perform the predicting process **(II)**, PNRSS was carried out with a sample mixture. The raw current trace was frequency split into the high and the low frequency portions. SVC was applied to label the events **(Fig. 5f)**. To generate the decision boundary, a mesh grid was generated within the area of 0-3 pA in the Lp S.D. and 1-4.5 pA in the Hp S.D. with a 0.01 pA interval. Event type regions can be identified by these mesh grid parameters when inferenced from the SVC model. The boundary separating these regions were taken as the decision boundaries **(Fig. 5f)**.

**
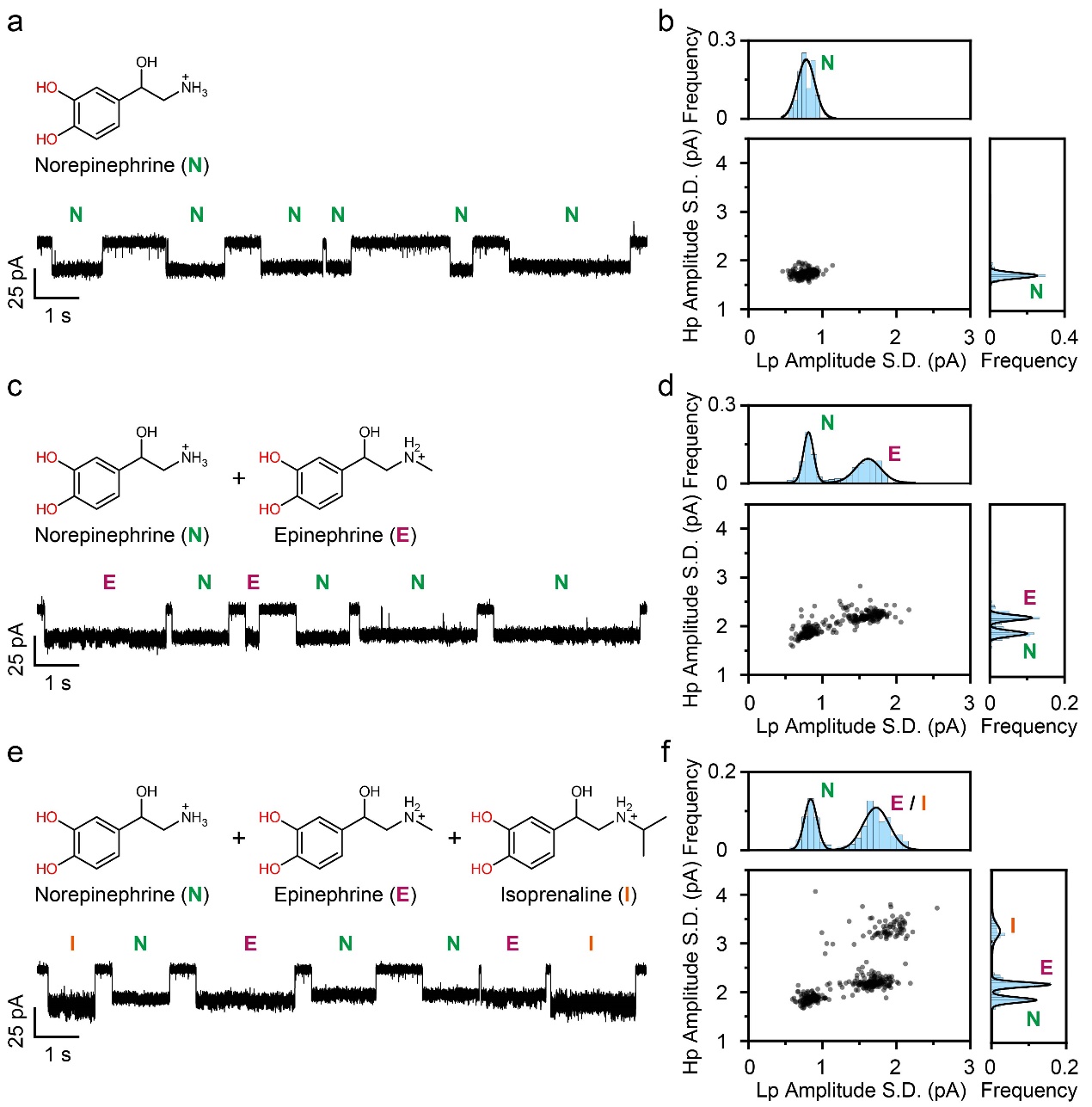
**

Supplementary fig. 46| Sequential addition of norepinephrine, epinephrine and isoprenaline. PNRSS measurements were carried out as described in **Fig. 5**. The buffer applied was 1.5 M KCl, 10 mM HEPES, pH 8.0. A +160 mV potential was continuously applied. Norepinephrine (N), epinephrine (E) and isoprenaline (I) were sequentially added to the *trans* compartment, reaching a 280 µM, a 280 µM and a 180 µM final concentration respectively. **a.** A representative trace acquired when only norepinephrine was added. b. The event scatter plot generated from a 15 min continuously recorded trace, acquired as described in a. 241 events are included in the scatter plot. **c.** A representative trace when epinephrine was further added. **d.** The corresponding event scatter plot generated from a 15 min trace acquired as described in **c**. 323 events are included in the scatter plot. e. A representative trace acquired when isoprenaline was further added. f. The corresponding event scatter plot generated from a 15 min trace acquired from the condition described in **e**. 388 events are included in the scatter plot. Event scatter plots were generated according to the low pass (Lp) and the high pass (Hp) standard deviation (S.D.) values of each event, as described in **Supplementary fig. 44**.

**
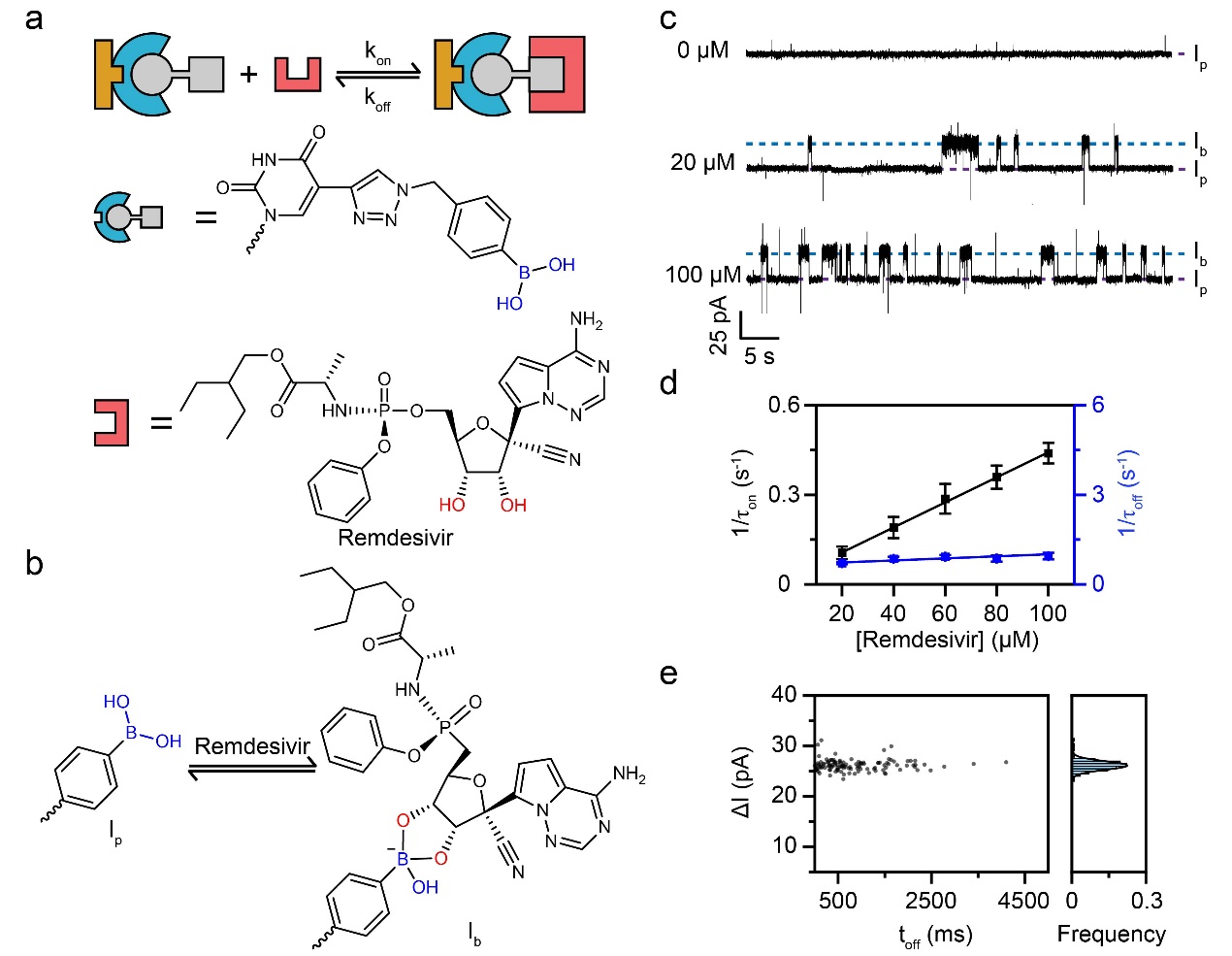
**

Supplementary fig. 47| Remdesivir binding to a PBA. **a.** The schematic diagram. The PNRSS strand 14PBA **(Supplementary Table 1)** contains a sole PBA at site 14, capable of binding remdesivir, as illustrated by the cartoon diagram. **b.** The reactive mechanism. The ribose moiety of remdesivir binds to the PBA, forming a boronate ester [^14^](#_ENREF_14). **c.** Representative traces containing remdesivir binding events. The electrolyte buffer was 1.5 M KCl, 10 mM HEPES, pH 8.0. A +160 mV potential was continuously applied. Remdesivir, dissolved in DMSO with a 10 mM concentration, was added to *trans* to reach a final concentration of 0-100 µM, marked on the left of each corresponding trace. The rate of event appearance increases when the remdesivir concentration is raised. **d.** Concentration dependence. The reciprocal of inter-event interval ($1/{\tau_{on}}$) and the reciprocal of dwell time ($1/{\tau_{off}}$) is plotted against the final concentration of remdesivir. $1/{\tau_{on}}$ demonstrates a linear correlation with the concentration of remdesivir. However, $1/{\tau_{off}}$ stays constant. Error bars=Standard Deviations (N=3). **e.** Scatter plot of $\Delta I$ vs. $t_{off}$. 118 events are included in the scatter plot. The histogram of $\Delta I$, superimposed with its Gaussian fitting result, is plotted to the right of the scatter plot. The remdesivir concentration was 80 µM. The events were extracted from a 15 min continuously recorded trace.


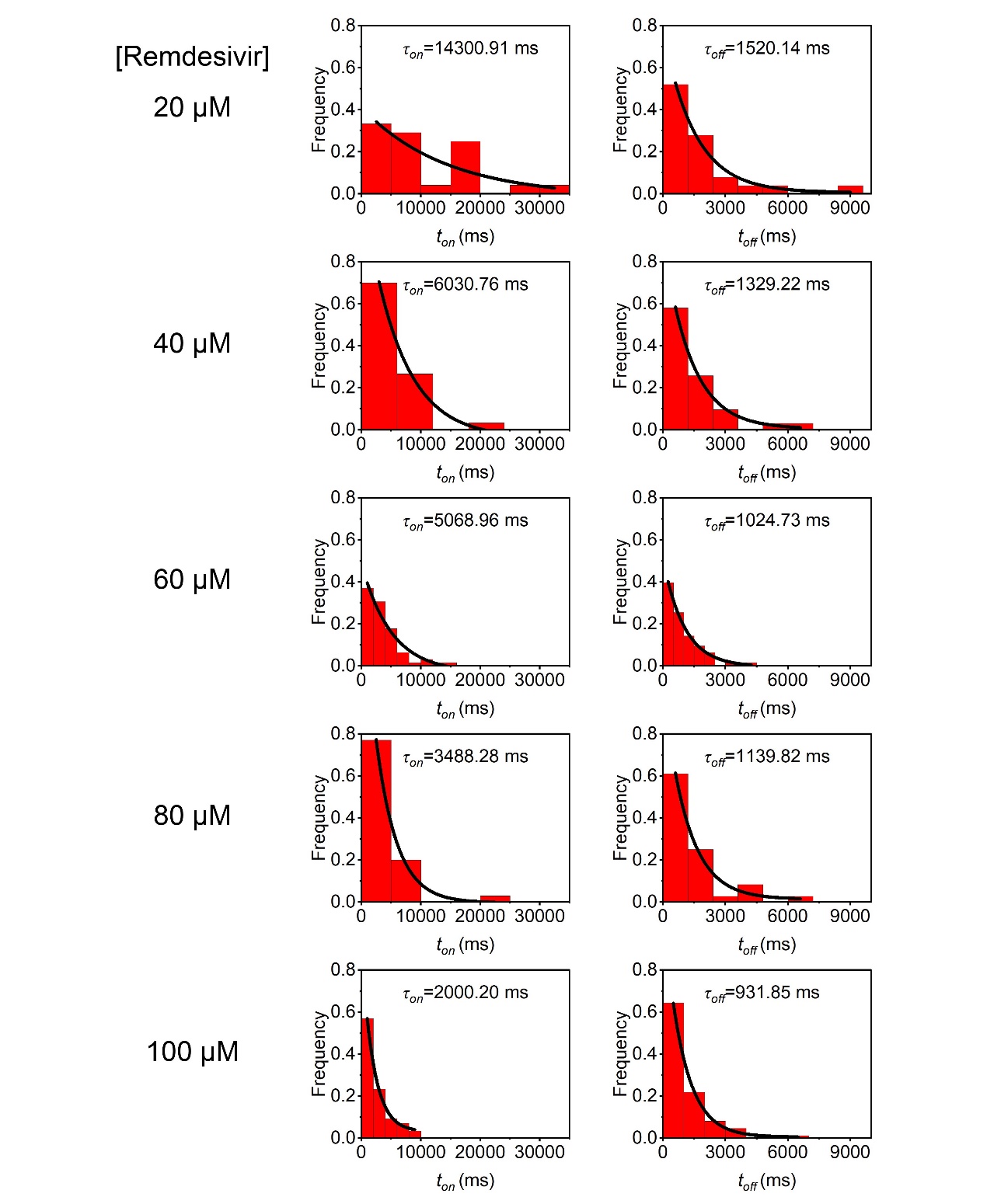


Supplementary fig. 48**|** $\boldsymbol{\tau}_{\boldsymbol{on}}$ **and** $\boldsymbol{\tau}_{\boldsymbol{off}}$ **of remdesivir binding to a PBA.** Histograms of the inter-event interval ($t_{on}$) and the event dwell time ($t_{off}$) with different remdesivir concentrations are presented. Remdesivir was added to *trans* with a final concentration of 20-100 µM. The applied concentration is marked on the left of each corresponding histogram plot. All histograms were respectively fit with a single exponential function $y=a*exp(-x/\tau)$, from which the mean inter-event interval ($\tau_{on}$) and the mean event dwell time ($\tau_{off}$) were derived and marked on each corresponding histogram plot. The PNRSS measurements were performed as described in **Methods**. The PNRSS strand 14PBA **(Supplementary Table 1)** was applied. A buffer of 1.5 M KCl, 10 mM HEPES, pH 8.0 was used. A +160 mV potential was continuously applied.

**
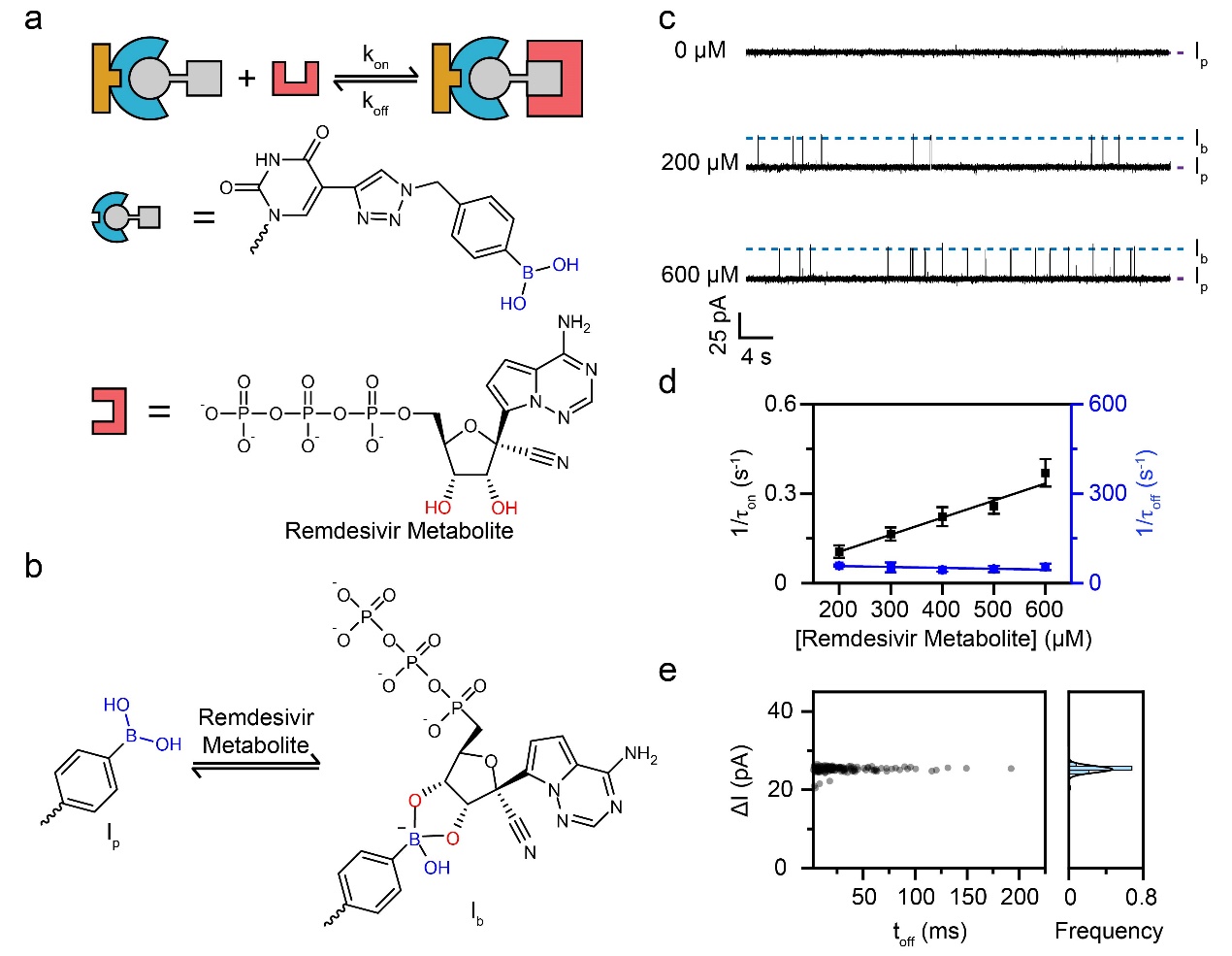
**

Supplementary fig. 49| Remdesivir metabolite binding to a PBA. **a.** The schematic diagram. The PNRSS strand 14PBA **(Supplementary Table 1)** contains a sole PBA at site 14, capable of binding the remdesivir triphosphate metabolite, as illustrated by the cartoon diagram. **b.** The reactive mechanism. The ribose moiety of remdesivir triphosphate metabolite binds to the PBA, forming a boronate ester[^14^](#_ENREF_14). **c.** Representative traces containing remdesivir triphosphate metabolite binding events. The electrolyte buffer was 1.5 M KCl, 10 mM HEPES, pH 8.0. Remdesivir triphosphate metabolite, originally dissolved in DMSO at a 10 mM concentation, was added to *trans* with a final concentration of 0-600 µM, marked on the left of each corresponding trace. A +160 mV potential was continuously applied. The rate of event appearance increases when the remdesivir triphosphate metabolite concentration is raised. **d.** Concentration dependence. The reciprocal of inter-event interval ($1/{\tau_{on}}$) and the reciprocal of dwell time ($1/{\tau_{off}}$) is plotted against the final concentration of remdesivir triphosphate metabolite. $1/{\tau_{on}}$ demonstrates a linear correlation with the concentration of remdesivir triphosphate metabolite. However, $1/{\tau_{off}}$ stays constant. Error bars=Standard Deviations (N=3). **e.** Scatter plot of $\Delta I$ vs. $t_{off}$. 130 events are included in the scatter plot. The histogram of $\Delta I$, superimposed with its Gaussian fitting result, is plotted to the right of the scatter plot. The remdesivir triphosphate metabolite concentration was 500 µM. The events were extracted from a 15 min continuously recorded trace.


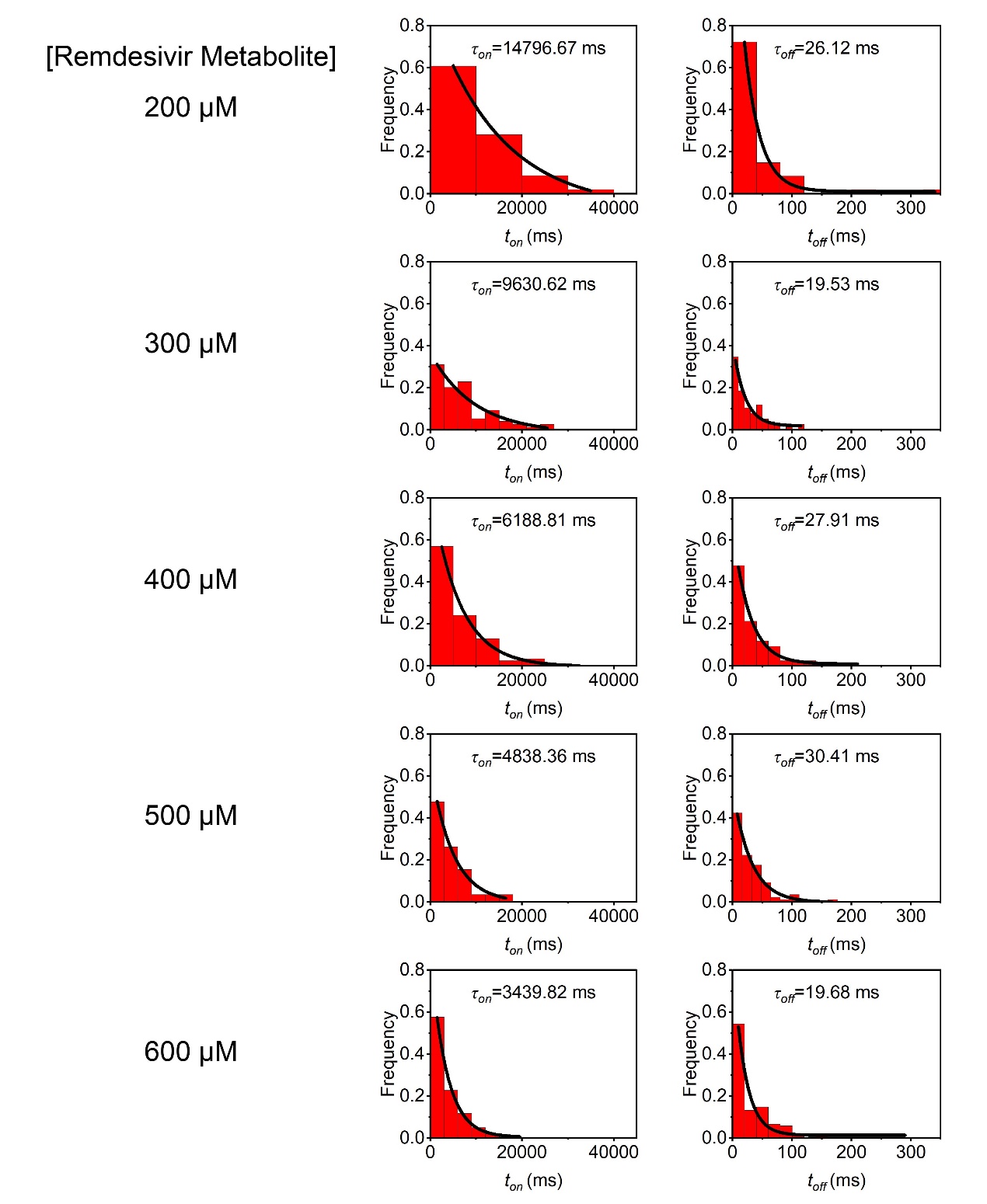


Supplementary fig. 50**|** $\boldsymbol{\tau}_{\boldsymbol{on}}$ **and** $\boldsymbol{\tau}_{\boldsymbol{off}}$ **of remdesivir metabolite binding to a PBA.** Histograms of the inter-event interval ($t_{on}$) and the event dwell time ($t_{off}$) with different remdesivir metabolite concentrations are presented. Remdesivir metabolite was added to *trans* with a final concentration of 200-600 µM. The applied concentration is marked on the left of each corresponding histogram plot. All histograms were respectively fit with a single exponential function $y=a*exp(-x/\tau)$, from which the mean inter-event interval ($\tau_{on}$) and the mean event dwell time ($\tau_{off}$) were derived and marked on each corresponding histogram plot. The PNRSS measurements were performed as described in **Methods**. The PNRSS strand 14PBA **(Supplementary Table 1)** was applied. A buffer of 1.5 M KCl, 10 mM HEPES, pH 8.0 was used. A +160 mV potential was continuously applied.

**
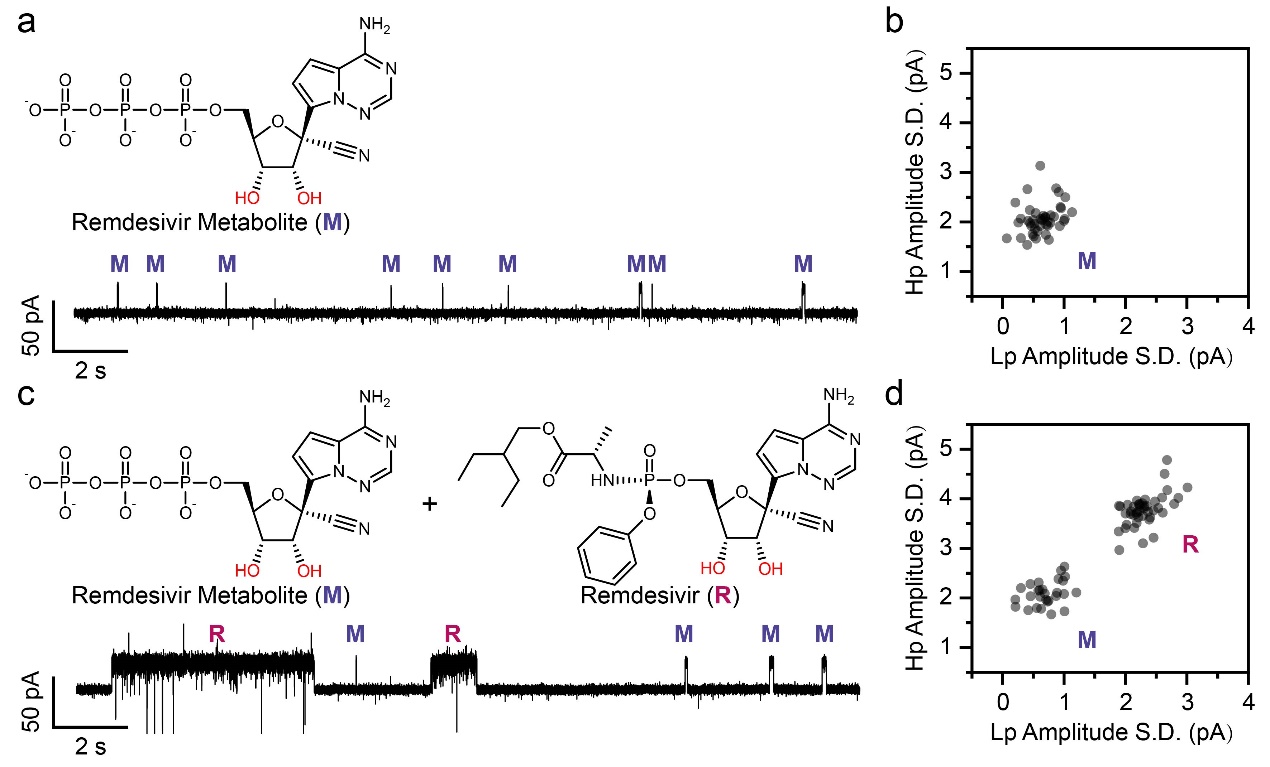
**

Supplementary fig. 51| Sequential addition of remdesivir metabolite and remdesivir. The PNRSS measurement was carried out as described in **Fig. 6**. The electrolyte buffer was 1.5 M KCl, 10 mM HEPES, pH 8.0. A +160 mV potential was continuously applied. Remdesivir metabolite (M) and remdesivir (R) were sequentially added to the *trans* compartment, reaching a 500 µM and a 20 µM final concentration respectively. **a**. The representative trace acquired when only remdesivir metabolite was added. Events of remdesivir metabolite binding are marked with purple M characters. b. The event scatter plot of low pass (Lp) standard deviation vs. the high pass (Hp) standard deviation from a 15 min continuously recorded trace, as described in a. 100 events are included in the scatter plot. Only remdesivir metabolite binding events (M) were observed in the scatter plot. c. The representative trace acquired when remdesivir was further added. The newly emerged events of remdesivir binding are marked with magenta R characters. d. The event scatter plot of low pass (Lp) standard deviation vs. the high pass (Hp) standard deviation from a 15 min continuously recorded trace, as described in c. 126 events are included in the scatter plot. Two clearly separated event populations are observed from the plot, demonstrating binding of remdesivir metabolite (M) and remdesivir (R) respectively.

**
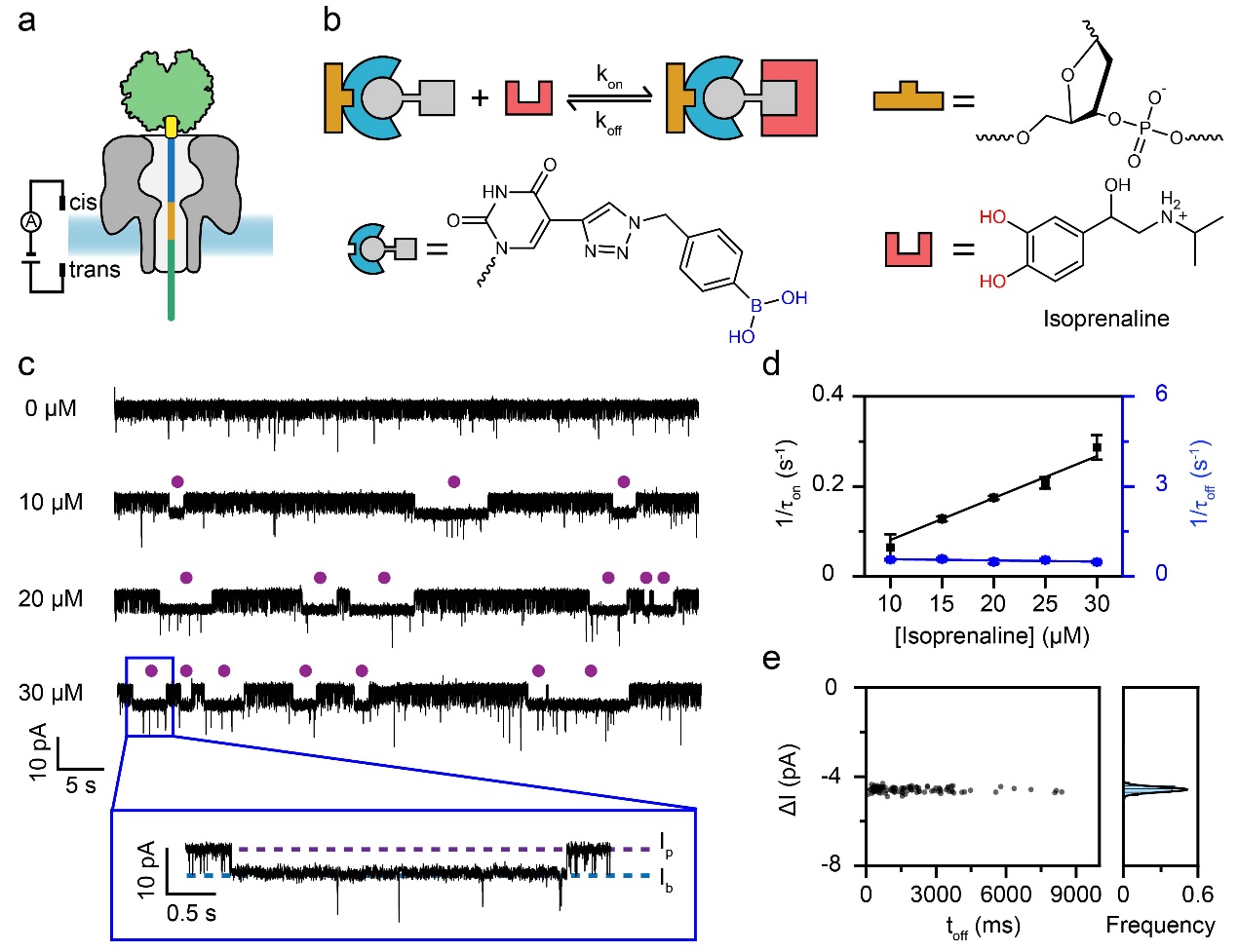
**

Supplementary fig. 52**| Demonstration of PNRSS with α-HL. a.** The measurement configuration. During PNRSS, an α-hemolysin (α-HL) nanopore serves to dock a streptavidin tethered PNRSS strand. **b.** The schematic diagram. The PNRSS strand 14PBA **(Supplementary Table 1)** contains a sole PBA at site 14, capable of binding an isoprenaline, as illustrated by the cartoon diagram. **c.** Representative traces containing isoprenaline binding events (purple circle). Even without the addition of isoprenaline, some negative going spiky noises were observed, indicating that the phenylboronic acid has detectable interactions with amino acid residues within the pore lumen. This is however not observed when measured with MspA. Binding of isoprenaline to a PBA results in negative going events. PNRSS measurements were carried out as described in **Methods**. The electrolyte buffer was 1.5 M KCl, 10 mM HEPES, pH 8.0. A +160 mV potential was continuously applied. Isoprenaline was added to *trans* with a final concentration of 0-30 µM. The applied concentration is marked on the left of each corresponding trace. The rate of event appearance increases when the isoprenaline concentration is raised. Inset: expanded view of the binding event. **d.** Concentration dependence. The isoprenaline concentration was modulated between 10-30 µM. 15 min continuous recording was performed for each condition. $\tau_{on}$ and $\tau_{off}$ values were derived as described in **Supplementary fig. 4**. Error bars=Standard Deviations (N=3). The reciprocal of inter-event interval ($1/{\tau_{on}}$) and the reciprocal of dwell time ($1/{\tau_{off}}$) are plotted against the final concentration of isoprenaline. $1/{\tau_{on}}$ demonstrates a linear correlation with the concentration of isoprenaline. However, $1/{\tau_{off}}$ stays constant. **e.** Event scatter plot of $\Delta I$ vs. $t_{off}$. The histogram of $\Delta I$, superimposed with its Gaussian fitting result, is plotted to the right of the scatter plot. A single population of events, measuring ~-4.6 pA in $\Delta I$ was identified. The isoprenaline concentration was 30 µM. The events were extracted from a 10 min continuously recorded trace. The number of binding events is 100.

**
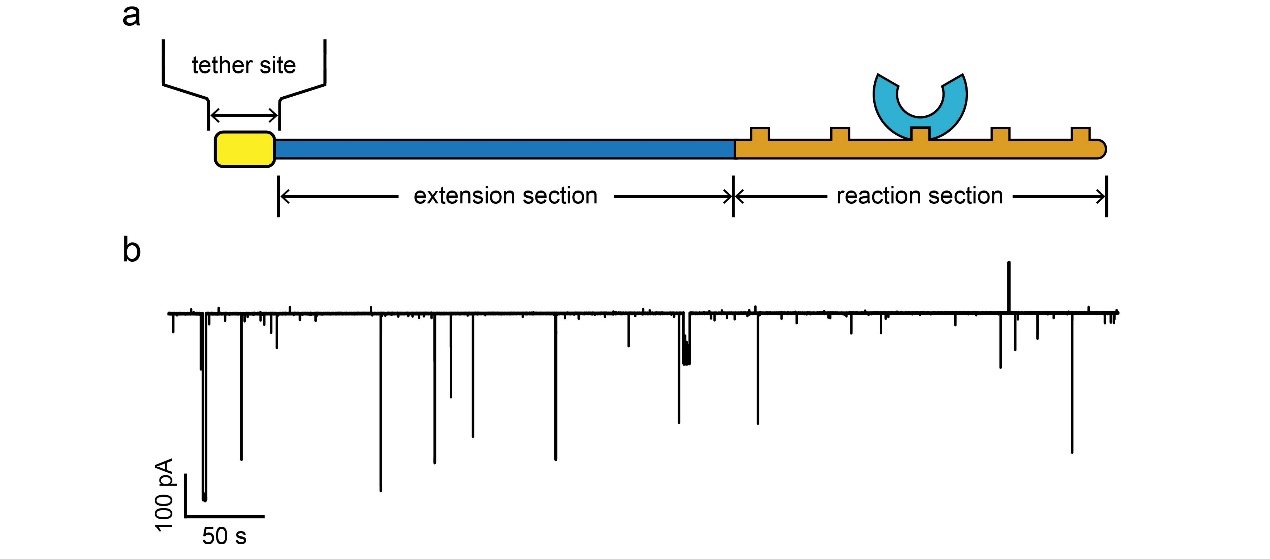
**

Supplementary fig. 53**| A PNRSS strand with no traction section. a.** The schematic diagram of a PNRSS strand 14TAK-NTS **(Supplementary Table 1)**. 14TAK-NTS has no traction section. **b.** A representative trace of PNRSS measurement with streptavidin tethered 14TAK-NTS. The measurement was carried out as described in **Methods**. A buffer of 1.5 M KCl, 10 mM HEPES, pH 8.0 was used. A +160 mV potential was continuously applied. Without the traction section, the streptavidin-tethered 14TAK-NTS can’t be efficiently trapped by MspA. Only transient pore blockages were observed. This confirms that the traction section of a PNRSS strand is critical to electrophoretically lead the strand into the pore and to maintain the strand in the pore lumen for a continuous measurement.

**
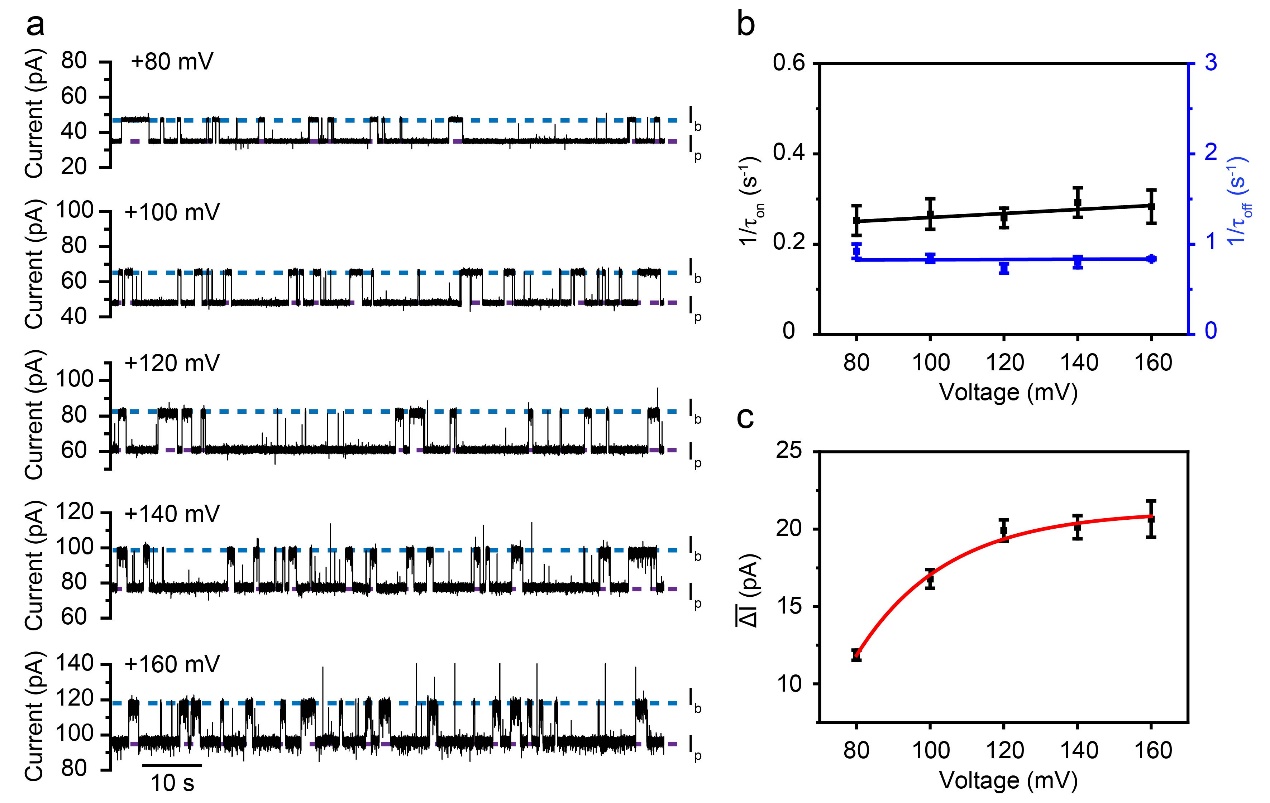
**

Supplementary fig. 54**| Catechol binding to a PBA measured at different voltages. a.** Representative traces for catechol binding to a PBA when a +80 mV, +100 mV, +120 mV, +140 mV or +160 mV voltage was applied. Catechol in *trans* was kept at a 200 µM concentration. Binding of catechol to a PBA results in positive going events. The rate of event appearance is generally unchanged when the applied voltage is modulated. The event amplitude ($\Delta I=I_{b}-I_{p}$) increases when the voltage is raised. **b.** A plot of $1/{\tau_{on}}$ or $1/{\tau_{off}}$ vs. the applied voltage. Both $1/{\tau_{on}}$ and $1/{\tau_{off}}$ generally stay constant when different voltages were applied. **c.** Plot of the mean event amplitude ($\bar{\Delta I}$) vs. the applied voltage. The mean event amplitude ($\bar{\Delta I}$) is larger when a larger voltage is applied. $\bar{\Delta I}$ is exponentially related to the applied voltage. The events were extracted from a 15 min continuously recorded trace. Error bars=Standard Deviations (N=3).

**
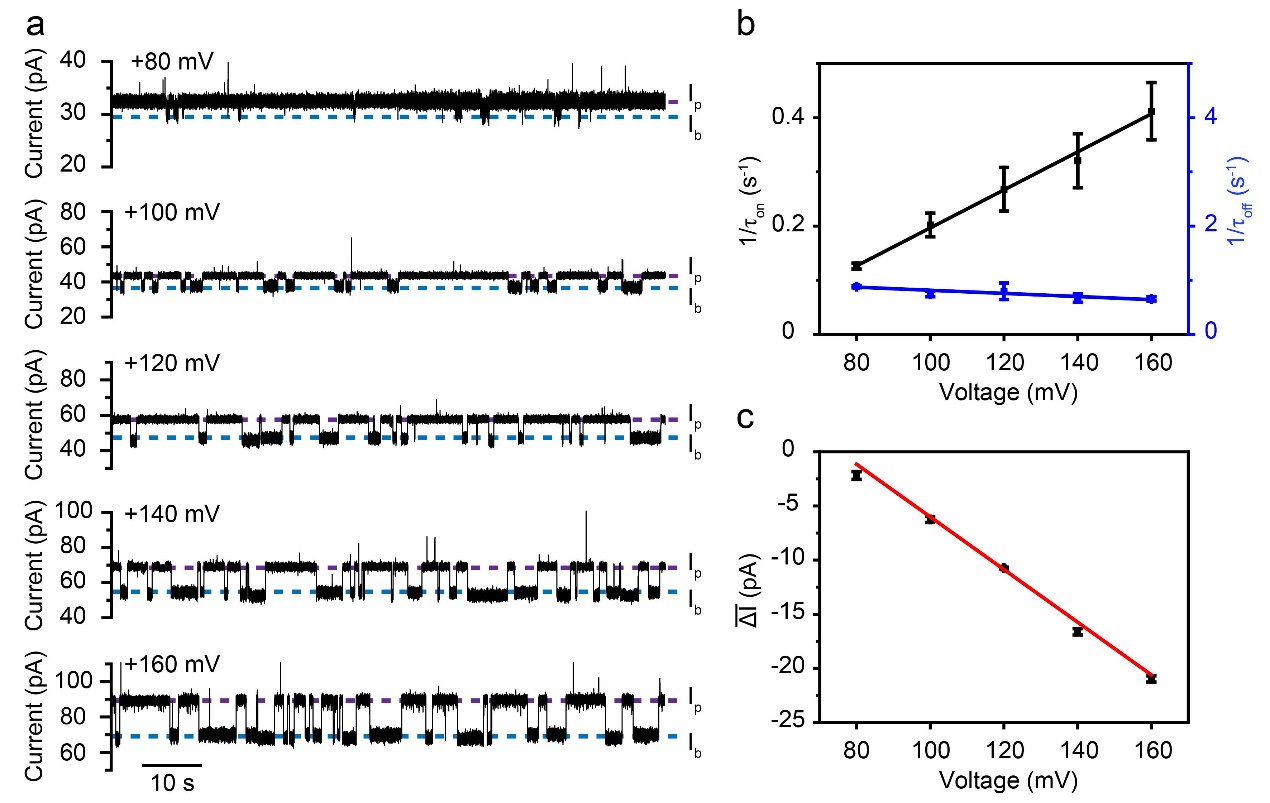
**

Supplementary fig. 55**| Norepinephrine binding to a PBA at different voltages. a.** Representative traces for norepinephrine binding to a PBA when a +80 mV, +100 mV, +120 mV, +140 mV or +160 mV voltage was applied. Norepinephrine in *trans* was kept at a 60 µM concentration. Binding of norepinephrine to a PBA results in negative going events. The rate of event appearance increases when the voltage is raised. The amplitude of event increases when the voltage is raised. **b.** A plot of $1/{\tau_{on}}$ or $1/{\tau_{off}}$ vs. the applied voltages. $1/{\tau_{on}}$ is linearly correlated to the voltage. However, $1/{\tau_{off}}$ stays constant. **c.** Plot of the mean event amplitude ($\bar{\Delta I}$) vs. the applied voltages. The absolute value of $\bar{\Delta I}$ increases when the voltage is raised and is linearly correlated with the voltage. The events were extracted from a 15 min continuously recorded trace. Error bars=Standard Deviations (N=3).


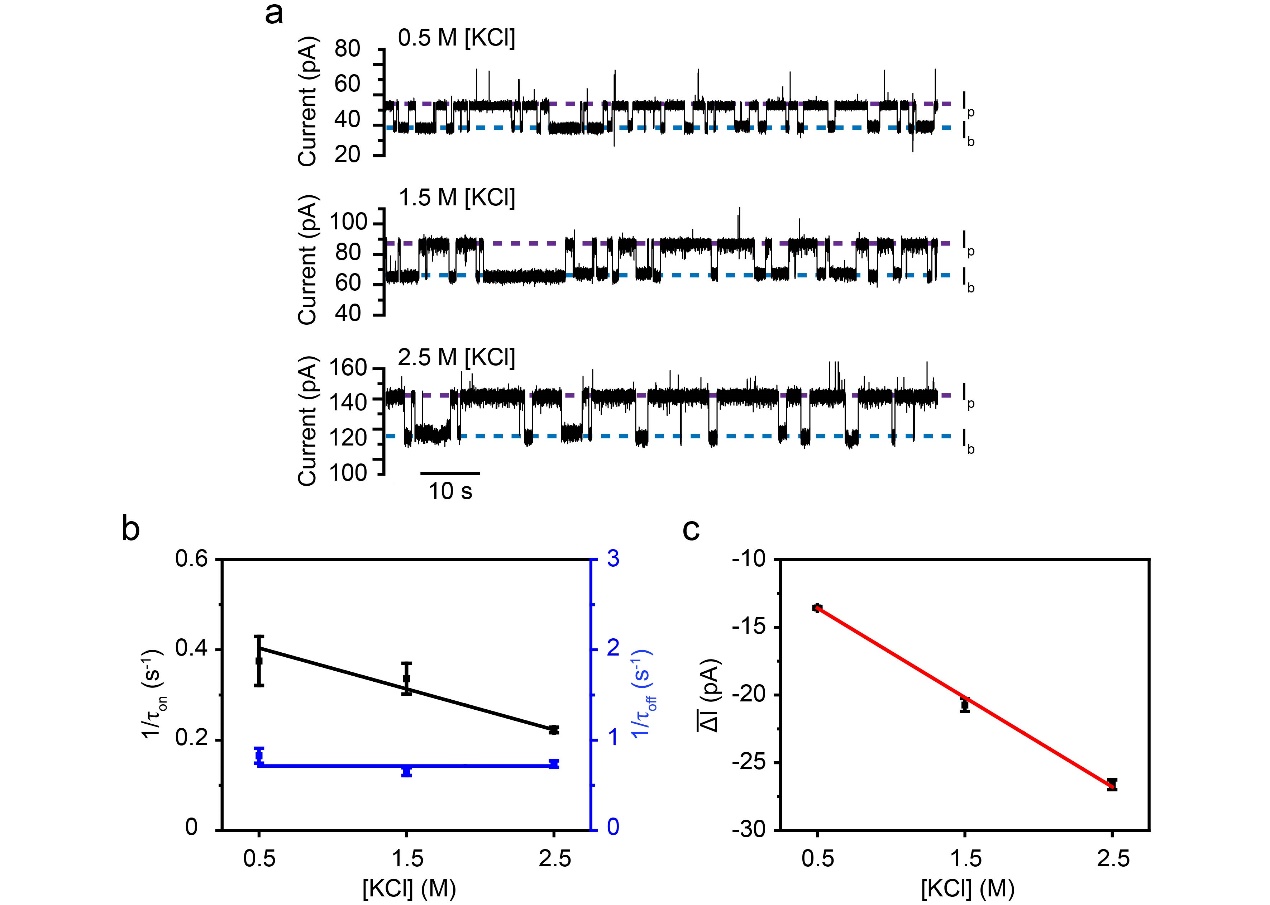


Supplementary fig. 56**| Norepinephrine binding to a PBA at different** **salt concentrations. a.** Representative traces containing events of norepinephrine binding to a PBA when a 0.5 M, 1.5 M or 2.5 M KCl electrolyte buffer (other components: 10 mM HEPES, pH 8.0) was applied. Norepinephrine in *trans* was kept at a 60 µM concentration. A +160 mV potential was continuously applied. Binding of norepinephrine to a PBA results in negative going events. The rate of event appearance decreases when the concentration of KCl is raised. However, the amplitude of event increases. **b.** A plot of $1/{\tau_{on}}$ or $1/{\tau_{off}}$ vs. the concentration of KCl in electrolyte buffer. $1/{\tau_{on}}$ is linearly negatively correlated to the [KCl], whereas $1/{\tau_{off}}$ stays constant. **c.** Plot of the mean event amplitude ($\bar{\Delta I}$) vs. the [KCl]. The absolute value of $\bar{\Delta I}$ increases when the [KCl] is raised. The events were extracted from a 15 min continuously recorded trace. Error bars=Standard Deviations (N=3).

**
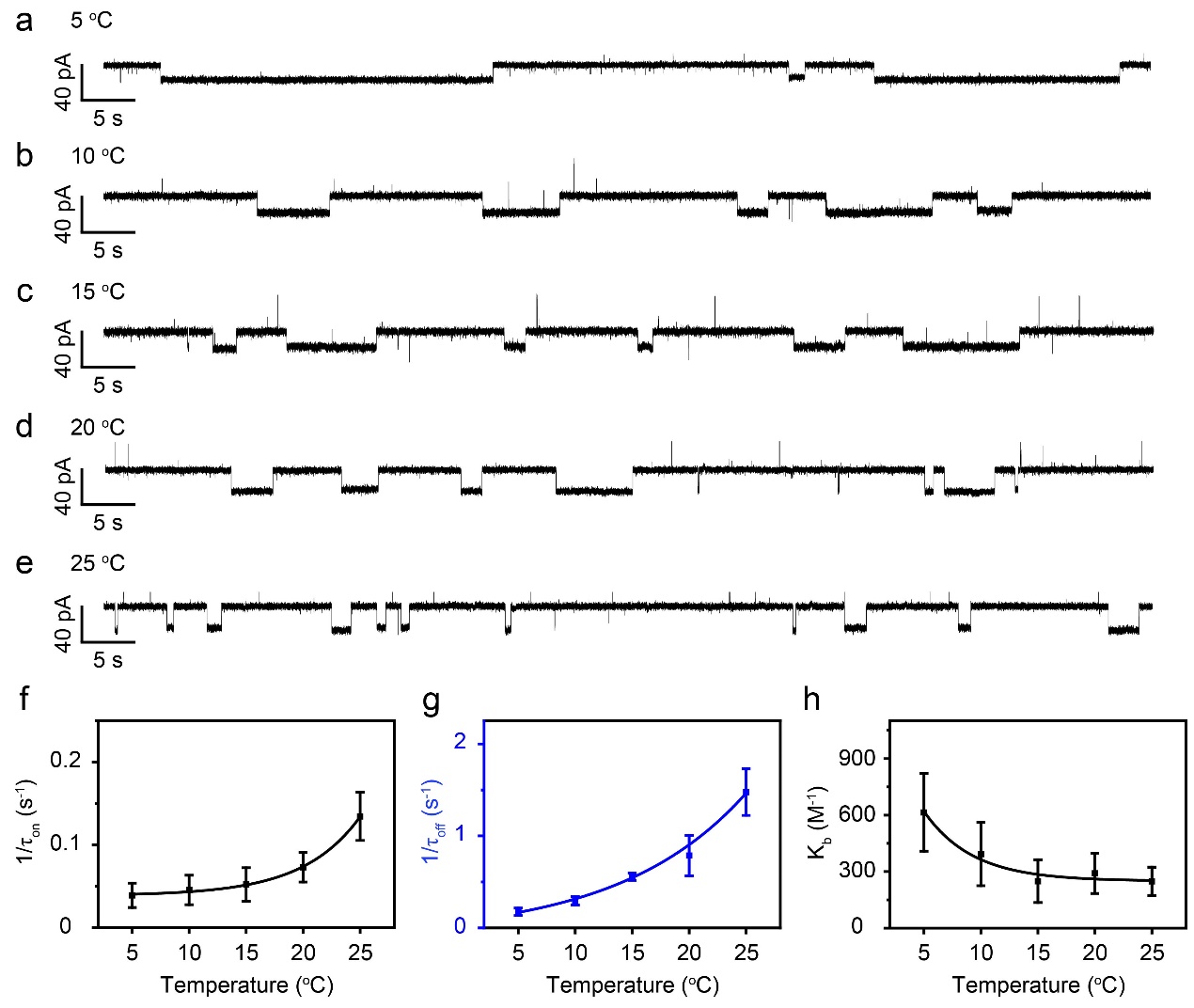
**

Supplementary fig. 57**| Norepinephrine binding to a PBA at different temperature. a-e.** Representative traces containing events of norepinephrine binding to a PBA when the temperature was set at 5 °C, 10 °C, 15 °C, 20 or 25°C. Norepinephrine in *cis* was kept at a 400 µM concentration. Binding of norepinephrine to a PBA results in negative going events. The rate of event appearance and the event dwell time increase when the temperature is raised. **f.** A plot of $1/{\tau_{on}}$ vs. the temperature. **g.** A plot of $1/{\tau_{off}}$ vs. the temperature. Both $1/{\tau_{on}}$ and $1/{\tau_{off}}$ are exponentially related to the temperature and could be described by the Arrhenius relation^[15](#_ENREF_15)^. **h.** A plot of $K_{b}$ vs. the temperature. $K_{b}$ decreases with the increase of the temperature. All measurements involved temperature variation were carried out with an Orbit Mini miniaturized bilayer workstation (Nanion Technologies GmbH, Germany) with a 1.25 kHz sampling rate and no further digital filtration. The events were extracted from a 15 min continuously recorded trace. Error bars=Standard Deviations (N=3).

**
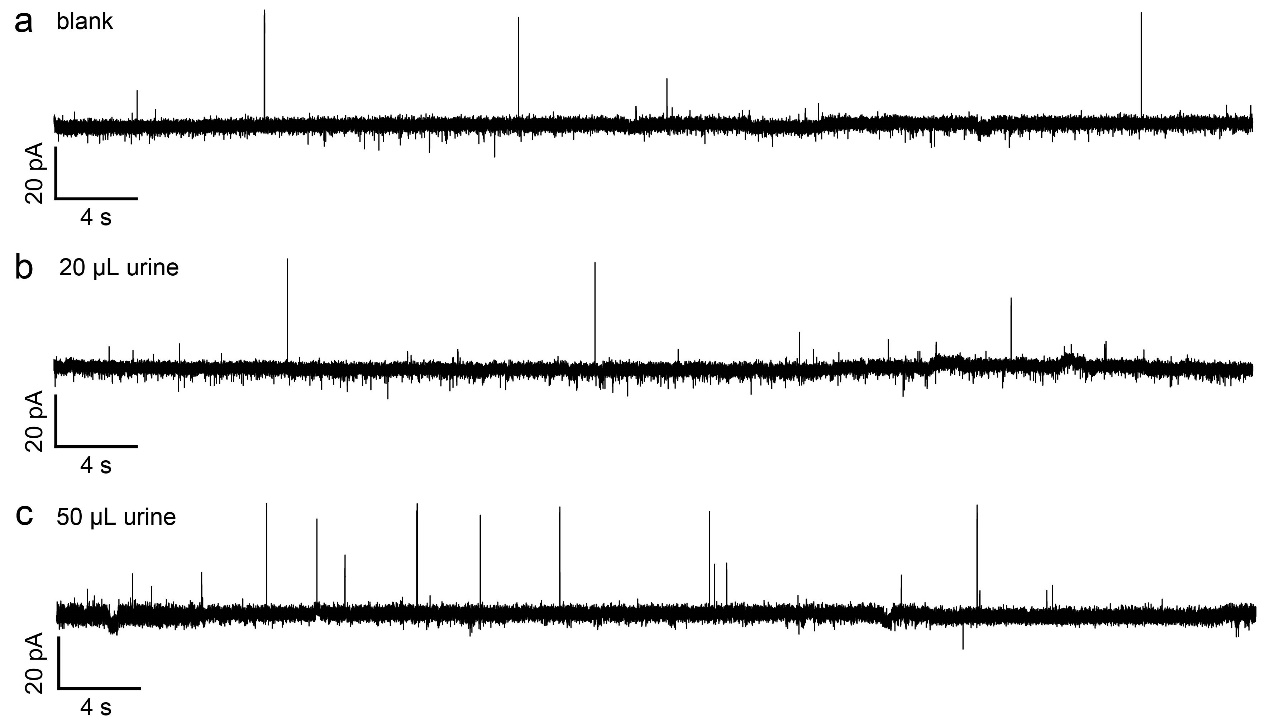
**

Supplementary fig. 58**| PNRSS detection of human urine samples.** The measurements were carried out as described in **Methods**. A buffer of 1.5 M KCl, 10 mM HEPES, pH 8.0. was used. A +160 mV potential was continuously applied. A PNRSS strand 14PBA **(Supplementary Table 1)** was applied. **a.** A representative PNRSS trace when no urine sample was added. Positive spiky events were stochastically prior to the addition of any urine sample. **b.** A representative trace when 20 µL human urine sample was added to *trans*. **c.** A representative trace when 50 µL urine sample was added to *trans*. No extra events were observed from human urine samples, confirming that urine sample is not generating any interfering events for this measurement.


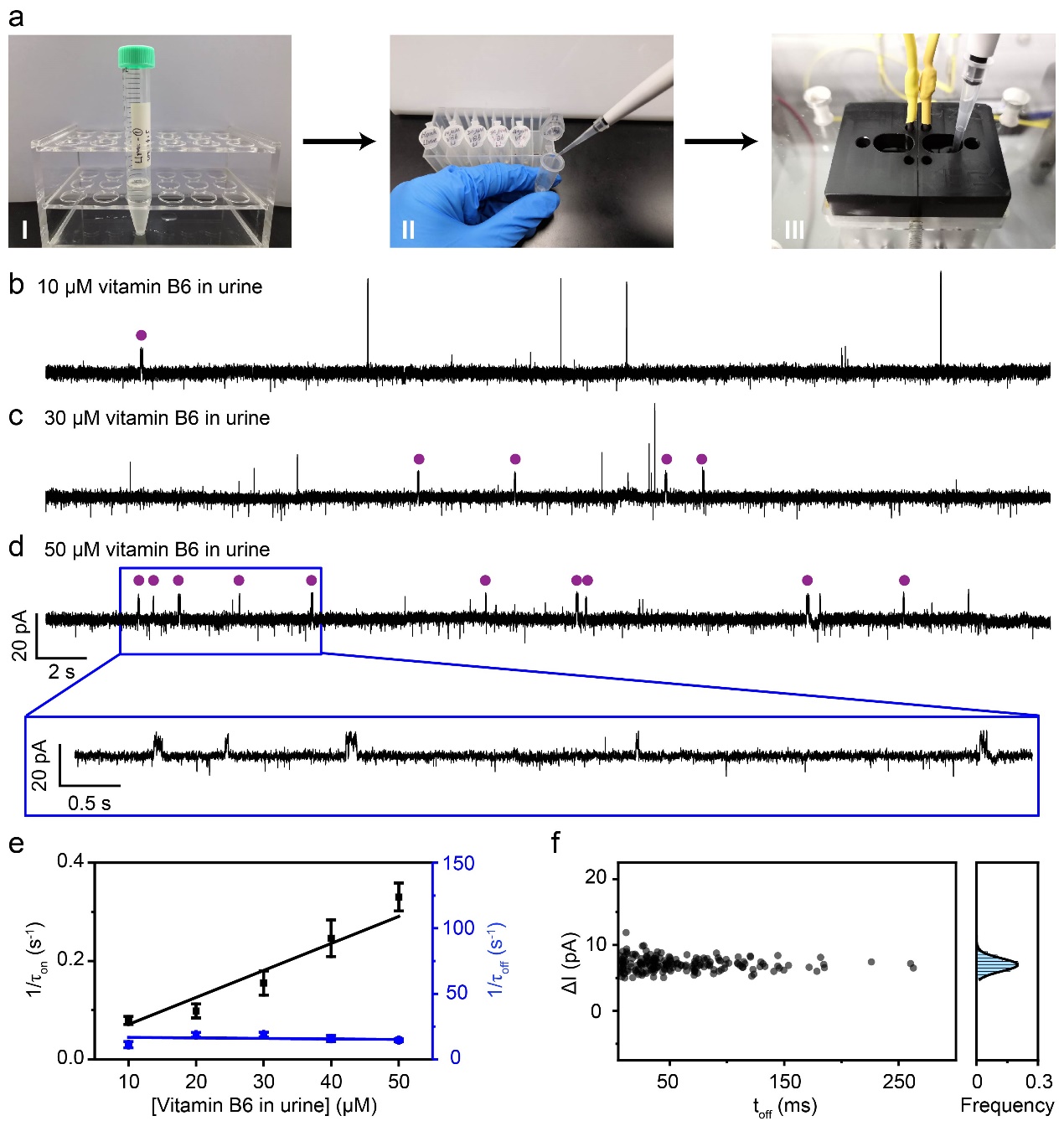


Supplementary fig. 59**| PNRSS detection of vitamin B6 in urine. a.** The workflow. The detection is performed by urine sample collection (I), premixing different concentrations of vitamin B6 in urine (II) and PNRSS detection (III). The urine sample was collected from a healthy volunteer (Asian, male, age 27). For the purpose of calibration and test of feasibility, vitamin B6 was added to urine samples to reach a final concentration of 10, 15, 20, 25 or 30 µM. 50 µL urine samples containing vitamin B6 were added to *trans* prior to each PNRSS measurement. **b-d.** Representative traces acquired with different urine samples. The events of vitamin B6 were marked with purple circles. Inset: an expanded view of the binding events. Generally, the rate of event appearance is increased when a higher concentration of vitamin B6 was added. **e.** A plot of $1/{\tau_{on}}$ or $1/{\tau_{off}}$ vs. the concentration of vitamin B6 in urine. ($1/{\tau_{on}}$) demonstrates a linear correlation with the concentration of vitamin B6 in urine. ($1/{\tau_{off}}$) stays constant. Error bars=Standard Deviations (N=3). **f.** Scatter plot of $\Delta I$ vs. $t_{off}$. 189 events are included in the scatter plot. The histogram of $\Delta I$, superimposed with its Gaussian fitting result, is plotted to the right of the scatter plot. A single population of events, measuring ~7.4 pA in $\Delta I$ was identified. The concentration vitamin B6 in urine was 40 µM. The events were extracted from a 10 min continuously recorded trace.

**References**

1. Masoud, M.S., Soayed, A.A. & Ali, A.E. Complexing properties of nucleic-acid constituents adenine and guanine complexes. *Spectrochimica Acta Part A: Molecular and Biomolecular Spectroscopy* **60**, 1907-1915 (2004).

2. Huq, F. & Peter, M.C.R. Interaction between NiCl2, and nucleobases, nucleosides and nucleotides. *Journal of Inorganic Biochemistry* **78**, 217-226 (2000).

3. Mai, K., Lin, J., Zhuang, B., Li, X. & Zhang, L.-M. Cationic dendronization of amylose via click chemistry for complexation and transfection of plasmid DNA. *International Journal of Biological Macromolecules* **79**, 209-216 (2015).

4. Struthers, H., Spingler, B., Mindt, T.L. & Schibli, R. “Click-to-Chelate”: Design and Incorporation of Triazole-Containing Metal-Chelating Systems into Biomolecules of Diagnostic and Therapeutic Interest. *Chemistry – A European Journal* **14**, 6173-6183 (2008).

5. Zhou, J., Stapleton, P., Haider, S. & Healy, J. Boronic acid inhibitors of the class A β-lactamase KPC-2. *Bioorganic & Medicinal Chemistry* **26**, 2921-2927 (2018).

6. Steinmeyer, J. & Wagenknecht, H.-A. Synthesis of DNA Modified with Boronic Acid: Compatibility to Copper(I)-Catalyzed Azide–Alkyne Cycloaddition. *Bioconjugate Chemistry* **29**, 431-436 (2018).

7. Dai, C. et al. The first chemical synthesis of boronic acid-modified DNA through a copper-free click reaction. *Chemical Communications* **47**, 3598-3600 (2011).

8. Pizer, R. & Babcock, L. Mechanism of the complexation of boron acids with catechol and substituted catechols. *Inorganic Chemistry* **16**, 1677-1681 (1977).

9. Pizer, R. & Tihal, C. Equilibria and reaction mechanism of the complexation of methylboronic acid with polyols. *Inorganic Chemistry* **31**, 3243-3247 (1992).

10. Köse, D.A. & Zümreoglu-Karan, B. Complexation of boric acid with vitamin C. *New Journal of Chemistry* **33**, 1874-1881 (2009).

11. Köse, D.A., Zumreoglu-Karan, B., Sahin, O. & Büyükgüngör, O. Boric acid complexes with thiamine (vitamin B1) and pyridoxine (vitamin B6). *Inorganica Chimica Acta* **413**, 77-83 (2014).

12. Zhang, S., Tang, Y., Chen, Y., Zhang, J. & Wei, Y. Boronic acid-modified polyhedral oligomeric silsesquioxanes on polydopamine-coated magnetized graphene oxide for selective and high-capacity extraction of the catecholamines epinephrine, dopamine and isoprenaline. *Microchimica Acta* **187**, 77 (2020).

13. Qi, F. et al. arXiv:1901.08013 (2018).

14. Martin, A.R., Vasseur, J.-J. & Smietana, M. Boron and nucleic acid chemistries: merging the best of both worlds. *Chemical Society Reviews* **42**, 5684-5713 (2013).

15. Peleg, M., Normand, M.D. & Corradini, M.G. The Arrhenius Equation Revisited. *Critical Reviews in Food Science and Nutrition* **52**, 830-851 (2012).
