# Supplementary material for: Programmable nano-reactors for stochastic sensing
Source: Nat Commun. 2021 Oct 4;12:5811. doi: 10.1038/s41467-021-26054-9 (PMC8490433; doi:10.1038/s41467-021-26054-9)
Supplement: Supplementary file 3 — Description of Additional Supplementary Files [file 41467_2021_26054_MOESM3_ESM.docx]

# Description of Additional Supplementary Files

**Supplementary Movie** 1| Ni^2+^ binding to a dual guanine reactant. The PNRSS measurement was carried out as described in **Fig. 1**. The electrolyte buffer applied was 1.5 M KCl, 10 mM HEPES, pH 7.0. The PNRSS strand 13G/14G **(Table S1)** was added to *cis* with a 10 nM final concentration. The dual guanine on 13G/14G serves as the fixed reactant. Ni^2+^, serving as the mobile reactant, was added to *trans* with a 1 mM final concentration. A +180 mV potential was continuously applied. Initially (the first ~0.1 s), the pore was unoccupied and an open pore current was reported (~575 pA). Afterwards, a PNRSS strand was captured and an immediate drop of current to ~170 pA was observed. Further binding events measuring around -60 pA in $\Delta I$ successively appeared (0.2-5 s). These events result from reversible binding of Ni^2+^ to a dual guanine reactant, as explained by **Figs. 1c-1d**.

**Supplementary Movie** 2| Ni^2+^ binding to a TAZ. The PNRSS measurement was carried out as described in **Fig. 2**. The electrolyte buffer applied was 1.5 M KCl, 10 mM HEPES, pH 7.0. The PNRSS strand 14TAZ **(Table S1)** was added to *cis* with a 10 nM final concentration. The TAZ serves as the fixed reactant. Ni^2+^, serving as the mobile reactant, was added to *trans* with a 1 mM final concentration. In this video, a PNRSS strand 14TAZ has already been captured by the pore. Events resulted from reversible binding of Ni^2+^ to a TAZ were continuously observed as further blockages. All events report characteristic noisy fluctuations when a Ni^2+^ was bound.

**Supplementary Movie** 3| Repetitive measurements of irreversible reactions. The PNRSS measurement was carried out as described in **Fig. 4**. The electrolyte buffer applied was 1.5 M KCl, 10 mM HEPES, pH 8.0. The PNRSS strand 14PBA **(Table S1)** was added to *cis* with a 10 nM final concentration. The phenylboronic acid (PBA) on a 14PBA serves as the fixed reactant. Hydrogen peroxide, serving as the mobile reactant, was added to *trans* with a 5.4 mM final concentration. Hydrogen peroxide could either reversibly bind to a PBA or irreversibly oxidize it to a phenol **(Figs. 4a, 4b)**. Initially, reversible binding of hydrogen peroxide to a PBA was continuously observed, reporting spiky, positive going events (2-80 s). Irreversible oxidation of PBA, which has inactivated the chemical reactivity of the fixed reactant, results in an event-free segment of the trace. By sequentially switching the applied potential to -100 mV (strand ejection) and +160 mV (strand reloading), the inactivated PNRSS strand was ejected and another reactive strand was loaded. By this special measurement mode of PNRSS, repetitive measurements of irreversible reactions can be carried out.

**Supplementary Movie** 4| Positive and negative going events acquired with a PBA. The PNRSS measurement was carried out as described in **fig. S4**. The electrolyte buffer applied was 1.5 M KCl, 10 mM HEPES, pH 8.0. The PNRSS strand 14PBA **(Table S1)** was added to *cis* with a 10 nM final concentration. The phenylboronic acid (PBA) on a 14PBA serves as the fixed reactant. Catechol or norepinephrine, acting as the mobile reactant, was added to *trans* with a 280 µM final concentration for each analyte. In this video, a PNRSS strand 14PBA was captured by the pore, reporting an $I_{p}$ value of ~100 pA. Binding of catechol or norepinephrine respectively report positive ($I_{b}>I_{p}$) or negative ($I_{b}<I_{p}$) going events. Binding of catechol or norepinephrine was respectively labelled with C or N on the trace.

**Supplementary Movie** 5| PNRSS sensing of epinephrine, norepinephrine and isoprenaline. The PNRSS measurement was carried out as described in **Fig. 5**. The electrolyte buffer applied was 1.5 M KCl, 10 mM HEPES, pH 8.0. The PNRSS strand 14PBA **(Table S1)** was added to *cis* with a 10 nM final concentration. The phenylboronic acid (PBA) on a 14PBA serves as the fixed reactant. Norepinephrine, epinephrine and isoprenaline, acting as the mobile reactant, were added to *trans* with a 280 µM, a 280 µM and a 180 µM final concentration respectively. A +160 mV potential was continuously applied. In this video, a PNRSS strand 14PBA was captured by the pore. Successive binding of norepinephrine, epinephrine or isoprenaline to a PBA were observed. The raw trace was frequency split into the low pass (lp, the top trace) and the high pass (hp, the bottom trace) portion. A Butterworth filter was applied to perform the frequency split. The cut off frequency was set to 100 Hz **(fig. S44)**. Event identification was carried out by a machine learning algorithm **(fig. S45)** and the identified events were labelled as N (norepinephrine), E (epinephrine) or I (isoprenaline) respectively.

**Supplementary Movie** 6| PNRSS sensing of remdesivir and its metabolite. The PNRSS measurement was carried out as described in **Fig. 6**. The electrolyte buffer applied was 1.5 M KCl, 10 mM HEPES, pH 8.0. The PNRSS strand 14PBA **(Table S1)** was added to *cis* with a 10 nM final concentration. The phenylboronic acid (PBA) on a 14PBA serves as the fixed reactant. Remdesivir and its metabolite, serving as the mobile reactant, were added to *trans* with a 20 µM or a 500 µM final concentration respectively. A +160 mV potential was continuously applied. Since the beginning of this video, a PNRSS strand 14PBA has already been captured by the pore. Binding of remdesivir or its metabolite to a PBA both results in positive going events **(Fig. 6b)**. According to their different binding characteristics **(Fig. 6b-d)**, remdesivir or its metabolite was identified and respectively labelled with R or M on the trace.

**Supplementary Movie** 7| An artistic demonstration of PNRSS. The core concept of PNRSS is to lower the technical hurdle of protein engineering to prepare a heterooligomeric nanopore nanoreactor. With PNRSS, the reactive component is used as separate modules in a toolbox. New applications are enabled by countless combinations of these modules. Repetitive engineering of the protein nanopore is however not required.
